# New 2,4-bis[(substituted-aminomethyl)phenyl]phenylquinazoline and 2,4-bis[(substituted-aminomethyl)phenyl]phenylquinoline Derivatives: Synthesis and Biological Evaluation as Novel Anticancer Agents by Targeting G-Quadruplex

**DOI:** 10.3390/ph17010030

**Published:** 2023-12-25

**Authors:** Jean Guillon, Marc Le Borgne, Vittoria Milano, Aurore Guédin-Beaurepaire, Stéphane Moreau, Noël Pinaud, Luisa Ronga, Solène Savrimoutou, Sandra Albenque-Rubio, Mathieu Marchivie, Haouraa Kalout, Charley Walker, Louise Chevallier, Corinne Buré, Eric Largy, Valérie Gabelica, Jean-Louis Mergny, Virginie Baylot, Jacky Ferrer, Yamina Idrissi, Edith Chevret, David Cappellen, Vanessa Desplat, Zsuzsanna Schelz, István Zupkó

**Affiliations:** 1 INSERM, CNRS, ARNA, U1212, UMR 5320, UFR des Sciences Pharmaceutiques, Univ. Bordeaux, F-33076 Bordeaux, France; jean.guillon@u-bordeaux.fr (J.G.); vickymilano88@gmail.com (V.M.); aurore.guedin-beaurepaire@u-bordeaux.fr (A.G.-B.); stephane.moreau@u-bordeaux.fr (S.M.); solene.savrimoutou@u-bordeaux.fr (S.S.); sandra.rubio@u-bordeaux.fr (S.A.-R.); hkalout171996@gmail.com (H.K.); charley.walker@etu.u-bordeaux.fr (C.W.); chevallier.louise@yahoo.fr (L.C.); 2 Small Molecules for Biological Targets Team, Centre de Recherche en Cancérologie de Lyon, Centre Léon Bérard, CNRS 5286, INSERM 1052, Université Claude Bernard Lyon 1, Univ. Lyon, F-69373 Lyon, France; 3 ISM—CNRS UMR 5255, Univ. Bordeaux, F-33405 Talence, France; noel.pinaud@u-bordeaux.fr; 4 E2S UPPA, CNRS, IPREM, Université de Pau et des Pays de l’Adour, F-64053 Pau, France; luisa.ronga@univ-pau.fr; 5 ICMCB—UMR 5026, Univ. Bordeaux, F-33608 Pessac, France; mathieu.marchivie@u-bordeaux.fr; 6 CNRS, INSERM, IECB, US1, UAR 3033, Univ. Bordeaux, F-33600 Pessac, France; corinne.bure@u-bordeaux.fr; 7 CNRS, INSERM, ARNA, UMR 5320, U1212, IECB, Univ. Bordeaux, F-33600 Pessac, France; eric.largy@u-bordeaux.fr (E.L.); valerie.gabelica@inserm.fr (V.G.); 8 Ecole Polytechnique, Laboratoire d’Optique et Biosciences, CNRS, INSERM, Institut Polytechnique de Paris, F-91120 Palaiseau, France; jean-louis.mergny@inserm.fr; 9 Centre de Recherche en Cancérologie de Marseille (CRCM), Institut Paoli-Calmettes, CNRS UMR7258, Inserm U1068, Univ. Aix Marseille, F-13009 Marseille, France; virginie.baylot@inserm.fr; 10 INSERM UMR1312, BRIC, Bordeaux Institute of Oncology, Univ. Bordeaux, F-33076 Bordeaux, France; jacky_ferrer@yahoo.fr (J.F.); yamina.idrissi@u-bordeaux.fr (Y.I.); edith.chevret@u-bordeaux.fr (E.C.); david.cappellen@u-bordeaux.fr (D.C.); vanessa.desplat@u-bordeaux.fr (V.D.); 11 Service Tumor Biology and Tumor Bank Laboratory, Groupe Hospitalier Bordeaux, CHU Bordeaux, F-33000 Bordeaux, France; 12 Institute of Pharmacodynamics and Biopharmacy, Faculty of Pharmacy, University of Szeged, 6720 Szeged, Hungary; schelz.zsuzsanna@szte.hu

**Keywords:** antiproliferative activities, 2,4-bis[(substituted-aminomethyl)phenyl]phenylquinazoline, 2,4-bis[(substituted-aminomethyl)phenyl]phenylquinoline, G-quadruplex, G4 ligands, FRET-melting, native electrospray mass spectrometry, telomerase activity

## Abstract

The syntheses of novel 2,4-bis[(substituted-aminomethyl)phenyl]phenylquinazolines **12** and 2,4-bis[(substituted-aminomethyl)phenyl]phenylquinolines **13** are reported here in six steps starting from various halogeno-quinazoline-2,4-(1*H*,3*H*)-diones or substituted anilines. The antiproliferative activities of the products were determined in vitro against a panel of breast (MCF-7 and MDA-MB-231), human adherent cervical (HeLa and SiHa), and ovarian (A2780) cell lines. Disubstituted 6- and 7-phenyl-bis(3-dimethylaminopropyl)aminomethylphenyl-quinazolines **12b**, **12f**, and **12i** displayed the most interesting antiproliferative activities against six human cancer cell lines. In the series of quinoline derivatives, 6-phenyl-bis(3-dimethylaminopropyl)aminomethylphenylquinoline **13a** proved to be the most active. G-quadruplexes (G4) stacked non-canonical nucleic acid structures found in specific G-rich DNA, or RNA sequences in the human genome are considered as potential targets for the development of anticancer agents. Then, as small aza-organic heterocyclic derivatives are well known to target and stabilize G4 structures, their ability to bind G4 structures have been determined through FRET melting, circular dichroism, and native mass spectrometry assays. Finally, telomerase inhibition ability has been also assessed using the MCF-7 cell line.

## 1. Introduction

Cancer is one of the most devastating diseases and the second leading cause of death around the world. In 2020, over 18.1 million cancer cases were estimated around the world; 9.3 million in men and 8.8 million in women [1]. Among them, the most common and frequently encountered types of cancers were breast, lung, colorectal, prostate, stomach, liver, cervix uteri, esophagus, and thyroid, and the most common causes of cancer-related mortality included lung, colon and rectum, liver, stomach, and breast cancers [1,2]. Different cancer treatments including chemotherapy, radiation therapy, hormone therapy, immunotherapy, photodynamic therapy, hyperthermia, stem cell therapy, targeted therapy, and surgery have been used to treat them [3]. In the case of chemotherapy, cancer cells increasingly display multidrug resistance, and patients develop severe side effects to these drugs, hindering their efficacy. Moreover, despite the recent and promising development of targeted therapies, all patients are not responsive to current drugs, fostering the need for new cytotoxic molecules.

Telomerase has emerged as an interesting primary target in cancer therapy, as 85–90% of cancer cells display telomerase activity, enhancing their indefinite proliferation and immortality. Indeed, this ribonucleoprotein reverse transcriptase enzyme, which adds telomere repeats to telomere DNA to maintain chromosome stability and integrity, acts as a key regulator of long-term proliferation. A working hypothesis in the targeting of telomeric DNA with drugs is that, if the telomere overhang DNA forms higher order structures, such as G-quadruplexes, the telomere capping machinery, including the telomerase, will be perturbed and proliferation arrested [4].

G-quadruplexes (G4) are single-stranded guanine-rich nucleic acid sequences which may fold into non-canonical four-stranded secondary structures. These structures are formed through π–π stacking of G-quartets which involve four guanines organized in a plane via eight Hoogsteen type H-bonding. G4-structures are stabilized in the presence of monovalent cations such as K^+^ or Na^+^. In addition, G4-forming motifs have been identified in oncogene promoter regions, highlighting the therapeutic potential for targeted gene regulation at the transcriptional level [5,6]. The most studied oncogene promoters of G4s include c-MYC, BCL-2, h-RAS, K-RAS, and c-KIT. Thus, both the inhibition of telomerase activity through G4 structure stabilization and the induction of the DNA damage response through telomere uncapping can prompt a proliferation arrest. Hence, small molecules which target and stabilize G4-structures in human genome could act as potential and interesting cancer therapeutic agents [7,8,9].

**Figure 1:**
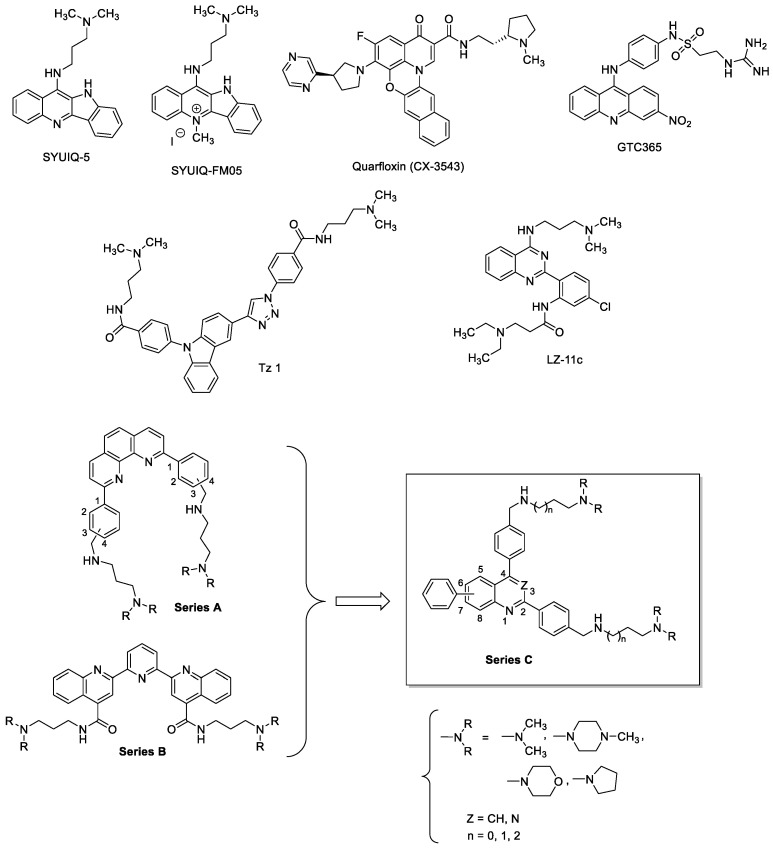
Structures of ligands SYUIQ-5, SYUIQ-5-FM05, quarfloxin, GTC365, Tz1, and LZ-11c compounds from series **A** and **B**, and new derivatives from series **C**.

The large planar aromatic surface of a terminal G-quartet provided a rationale for the design and development of planar G4 ligands such as polyaromatic fused molecules, for instance, acridines, phenanthroline, quinolone, and quinone [10,11,12,13,14,15]. Based on these chemical pharmacophores, a number of G4 binding molecules have been designed and developed over the last two decades, such as SYUIQ-5, SYUIQ-FM05, Quarfloxin, GTC365, Tz 1, or LZ-11c (Figure 1) [11,12,13,14,15,16,17]. Many of these heterocyclic ligands are selective for G4 structures over duplex DNA, but the design of a ligand specific for a given G4 structure remains challenging. In addition, quinoline and quinazoline have emerged as valuable scaffolds in medicinal chemistry possessing a diversity of biological activities, such as anticancer activities [18,19,20,21].

In the course of our research devoted to the design and the discovery of novel heterocycles for cancer chemotherapies [22,23,24,25,26,27,28,29], we previously designed and prepared two series of new substituted 2,9-bis[(substituted-aminomethyl)phenyl]-1,10-phenanthrolines A and diquinolinyl-pyridines B (Figure 1) designed to bind to DNA G-quadruplexes [25,28], and endowed with interesting and promising activity towards human leukemia cells. In this context, and through considering the pharmacological activities of these previous series A and B on human leukemia cells [25,28], we undertook the design and the synthesis of a new series of 2,4-bis[(substituted-aminomethyl)phenyl]phenylquinazoline and 2,4-bis[(substituted-aminomethyl)phenyl]phenylquinoline derivatives **12**,**13** (series C), novel structural analogues of these latter series A and B. The antiproliferative activities of these new derivatives were herein determined in vitro against a panel of breast (MCF-7 and MDA-MB-231), human adherent cervical (HeLa and SiHa), and ovarian (A2780) cell lines. In addition, the antiproliferative activity of the obtained derivatives **12**,**13** was then evaluated in vitro against the hematologic malignant K562 cell line.

We also evaluated ligand-induced stabilization, specificity, and selectivity of the newly synthesized 2,4-bis[(substituted-aminomethyl)phenyl]phenylquinazoline and 2,4-bis[(substituted-aminomethyl)phenyl]phenylquinoline derivatives **12**,**13** for various oncogene promoter G4 topologies, including c-MYC, BCL-2, and K-RAS through FRET-melting experiments.

## 2. Results & Discussion

### 2.1. Chemistry

**Scheme 1:**
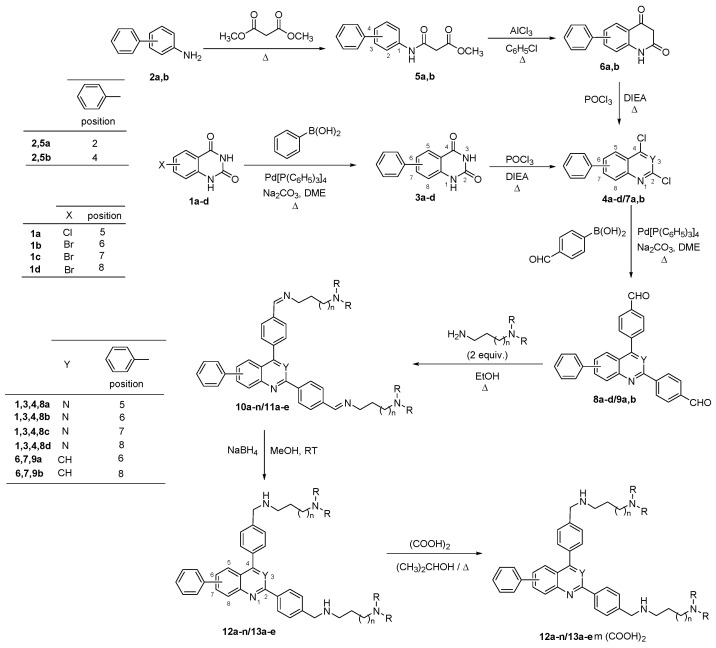

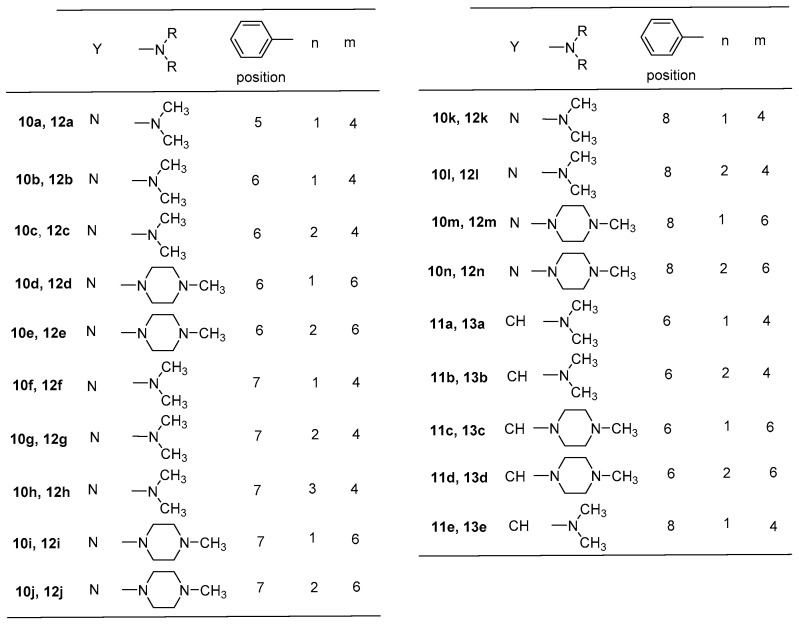
Synthesis of the 2,4-bis[(substituted-aminomethyl)phenyl]phenylquinazolines **12a–n** and 2,4-bis[(substituted-aminomethyl)phenyl]quinolines **13a–e**.

These new 2,4-bis[(substituted-aminomethyl)phenyl]phenylquinazoline and 2,4-bis[(substituted-aminomethyl)phenyl]phenylquinoline derivatives **12**,**13** were each prepared in six steps starting from various halogeno-quinazoline-2,4-(1H,3H)-diones **1** or substituted anilines **2** (Scheme 1). 

**Figure 2:**
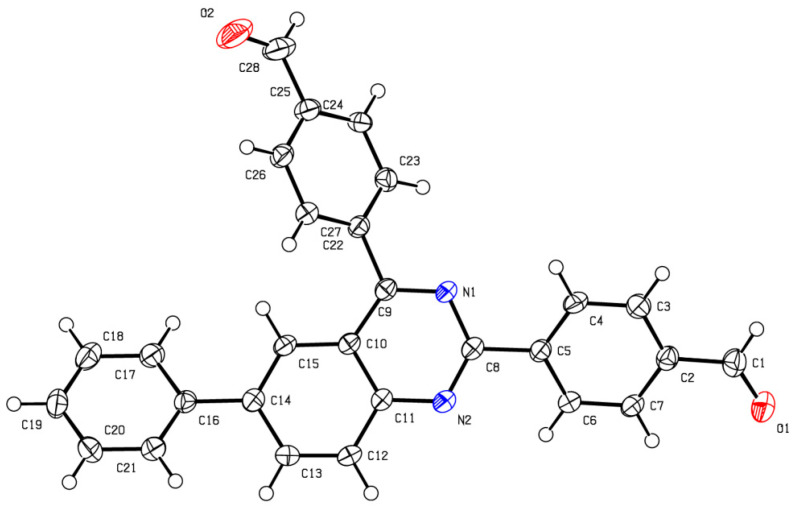
The ORTEP (Oak Ridge Thermal Ellipsoid Plot) drawing of compound **8b** with thermal ellipsoids at 30% level.

**Figure 3:**
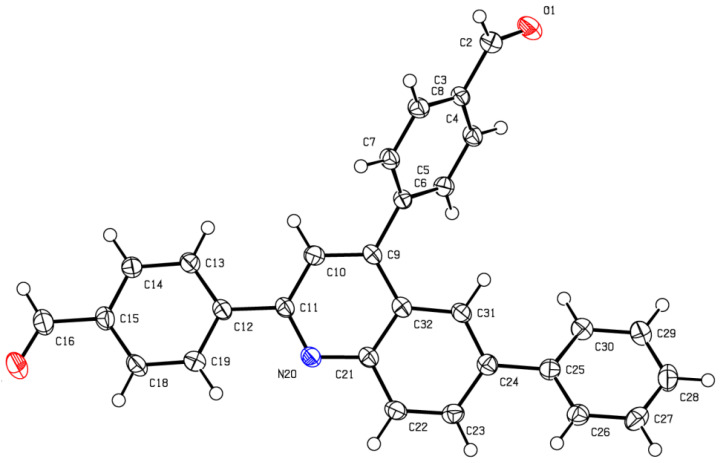
The ORTEP (Oak Ridge Thermal Ellipsoid Plot) drawing of compound **9a** with thermal ellipsoids at 30% level.

Suzuki coupling reaction with compounds **1a**–**d** with phenyl boronic acid provided the different phenyl-quinazoline-2,4-(1H,3H)-diones **3a**–**d**, which then reacted with phosphorous oxychloride in the presence of diisopropylethylamine to generate dichloro derivatives **4a**–**d**. The addition of dimethyl malonate to phenylanilines **2a**,**b** provided amido-esters **5a**,**b**. Quinoline-2,4-diones **6a**,**b** were then prepared by intramolecular cyclization of amido-esters **5a**,**b** in chlorobenzene using AlCl_3_. The lactams **6a**,**b** were subsequently chlorodehydroxylated with phosphorous oxychloride, leading to 2,4-dichloroquinolines **7a**,**b**. The intermediates bis-(formylphenyl)-quinazolines and -quinolines **8**–**9** were synthesized by a double-Suzuki-Miyaura cross-coupling reaction of the dichloro derivatives **4**,**7** with 4-formylphenylboronic acids in the presence of Pd(PPh_3_)_4_ as a catalyst and in the presence of sodium carbonate. The 3D structural determination of two new substituted derivatives **8b** and **9a** was established by X-ray crystallography (Figure 2 and Figure 3) [30] and confirmed the structure in the solid state as anticipated on the basis of NMR data. 

**Scheme 2:**
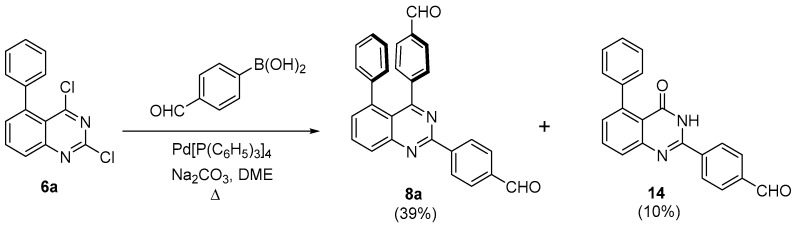
Synthesis of 2,4-bis(4-formylphenyl)-5-phenylquinazoline **8a** and 2-(4-formylphenyl)-5-phenyl-3*H*-quinazolin-4-one **14**.

During the synthesis of compound **8a**, we also isolated the 2-(4-formylphenyl)-5-phenyl-3H-quinazolin-4-one **14** as a side product (Scheme 2). 

The structure of the latter derivative **14** was confirmed by a complete NMR analysis and was consistent with previously described 5-substituted-3H-quinazolin-4-ones [31]. Condensation of various primary amines with these dialdehydes **8**,**9** provided the di-imines **10**,**11**, which were immediately reduced into the 2,4-bis[(substituted-aminomethyl)phenyl]phenylquinazolines **12a**–**n** and 2,4-bis[(substituted-aminomethyl)phenyl]quinolines **13a**–**e** using sodium borohydride as a reductive agent in refluxing methanol as previously described by our team (Scheme 1). 

Our quinazolines and quinolines **12**,**13** were then converted into ammonium oxalate salts via treatment with oxalic acid in refluxing isopropanol. These oxalate salts were less hygroscopic than the hydrochloride ones and were also soluble in water. Table S1 (in the Supplementary Materials) summarizes the physical properties of the new synthesized **12**,**13** oxalates. 

**Scheme 3:**
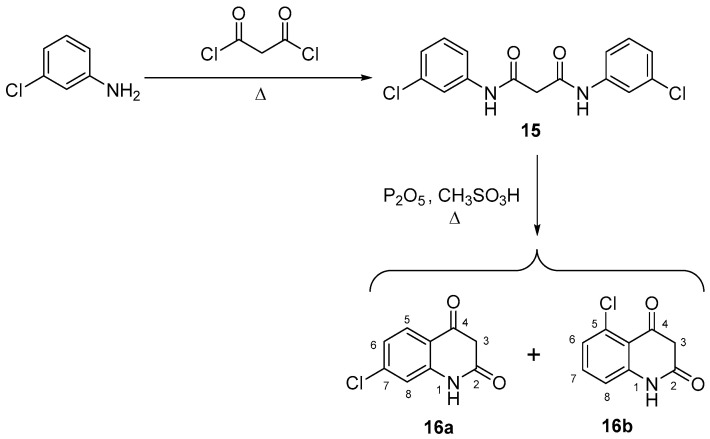
Attempt to synthesize halogeno-quinoline-2,4-diones **16a**.

The different attempts to synthesize halogeno-quinoline-2,4-diones **16a** and **16b** from the diamide **15**, previously prepared from 3-chloroaniline with malonyl chloride, and using a mixture of phosphorus pentoxide and methanesulfonic acid, led to a mixture of compounds **16a**,**b** for which the separation has always failed (Scheme 3) [32]. 

### 2.2. Biology

**Figure 4:**
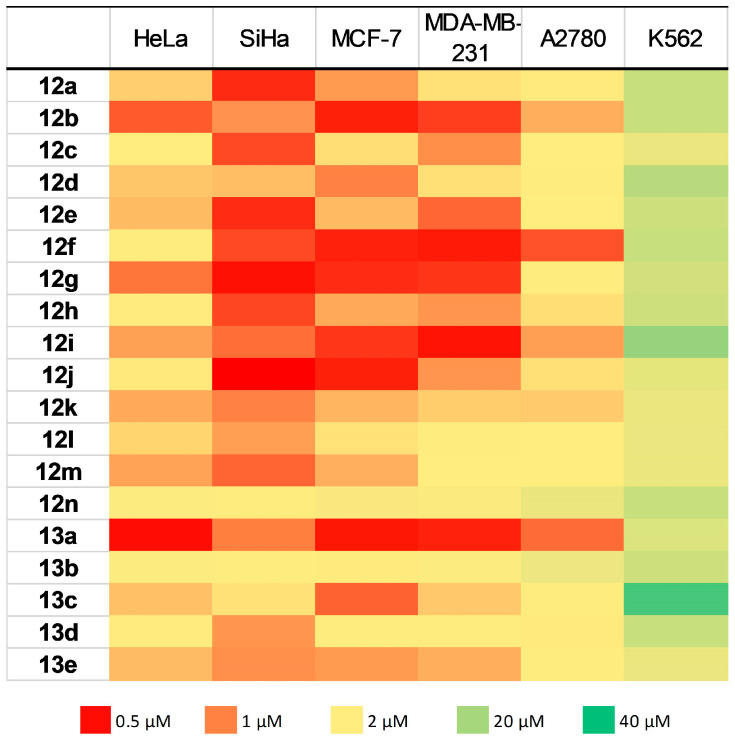
Heatmap of the IC_50_ values of compounds **12a–n** and **13a–e**. The values are calculated from the results of two independent determinations.

The in vitro antiproliferative capacity of 2,4-bis[(substituted-aminomethyl)phenyl]phenylquinazolines **12a**–**n** and 2,4-bis[(substituted-aminomethyl)phenyl]quinolines **13a**–**e** was then evaluated on a panel of human adherent cancer cell lines. These novel derivatives were tested on cervical (HeLa and SiHa), ovarian (A2780), and breast (MCF-7 and MDA-MB-231) cell lines. The antiproliferative IC_50_ values of 2,4-bis[(substituted-aminomethyl)phenyl]phenylquinazolines **12a**–**n** and 2,4-bis[(substituted-aminomethyl)phenyl]quinolines **13a**–**e** ranged from 0.33 to 7.10 μM. Additionally, the antiproliferative activity of the new derivatives **12**,**13** was also evaluated in vitro using the hematologic malignant K562 cell line. MTT assays were conducted and revealed that all compounds exhibited considerable activities (Figure 4, Table 1). Furthermore, adherent cell lines of gynecological origin were substantially more sensitive than leukemia cells. Based on these obtained biological activities, some conclusions could be formulated concerning the possible structure–activity relationships. 

Against the cervical HeLa cell line, the new derivatives **12**,**13** showed some significant antiproliferative activities, with an IC_50_ between 0.50 and 3.58 μM. Among these compounds **12**,**13**, the 2,4-bis{4-[(3-dimethylaminopropyl)aminomethyl]phenyl}-6-phenylquinoline **13a** exhibited the best antiproliferative activity (IC_50_ = 0.50 μM). In terms of structure–activity relationships, when we compared quinazolines **12a**, **12b**, **12f**, and **12k** bearing two (3-dimethylaminopropyl)aminomethylphenyl side chains and particularly the phenyl ring in positions 5, 6, 7, and 8 of the quinazoline heterocyclic moiety, respectively, derivative **12b** was more active than its other homologous (IC_50_ = 0.82 μM versus 1.19–1.51 μM for compounds **12a**, **12f**, and **12k**). The same experiments in the quinoline series showed that derivative **13a**, substituted by the phenyl group in position 6, was more active against the HeLa cell line than its analogue **13e**, substituted at position 8; i.e., IC_50_ = 0.50 μM for **13a** and 1.28 μM for **13e**. A comparison of compounds **12b** and **13a**, both substituted by (3-dimethylaminopropyl)aminomethylphenyl side chains and also by a phenyl in position 6, but different by the basic heterocyclic system, quinazoline versus quinoline, revealed that the quinoline skeleton presented a more promising antiproliferative activity (IC_50_ = 0.50 μM for **13a** and 0.82 μM for **12b**). In addition, the disubstituted 7-phenylquinazoline **12g**, which was disubstituted with two C4 dimethylaminoalkylaminobenzyl chains, exhibited a better biological activity (IC_50_ = 0.95 μM) than its C3 or C5 homologs, compounds **12f** and **12h**, with IC_50_ values of 1.51 μM and 2.31 μM, respectively. Similar comparisons between the disubstituted 6-phenylquinazolines **12b** and **12c** showed that elongation of the alkyl side chain (propyl versus butyl) led to a drop in the antiproliferative activity against HeLa cells (IC_50_ = 0.82 μM for **12b** and 2.21 μM for **12c**). This observation was consistent with 8-phenyl-derivatives **12m** and **12n**, substituted by 3-(4-methylpiperazin-1-yl)propyl)aminobenzyl or 4-(4-methylpiperazin-1-yl)butyl)aminobenzyl side chains, as in quinoline compounds **13c** and **13d** with a phenyl ring in position 6 and displayed an IC_50_ =1.30 and 2.27 μM, respectively.

In the case of the cervical SiHa cancer cell line, quinazoline **12j** was the most active compound with an IC_50_ of 0.33 μM. By comparing compounds **12a**, **12b**, **12f** and **12k** all bearing two (3-dimethylaminopropyl)aminomethylphenyl side chains and a phenyl ring linked to the quinazoline skeleton in positions 5, 6, 7 and 8, respectively, **12a** was slightly more active than its analogues (IC_50_ = 0.62 μM versus 0.75–1.08 μM). When we compared 6-phenylquinazolines **12d** versus **12e**, then 7-phenylquinazolines **12i** versus **12j**, we noticed that the heterocyclic moieties substituted by 4-(4-methylpiperazin-1-yl)butyl)aminobenzyl side chains exhibited more interesting cytotoxicity than those bearing 3-(4-methylpiperazin-1-yl)propyl)aminobenzyl side chains; i.e., IC_50_ = 0.63 μM for **12e** and 1.28 μM for **12d**, IC_50_ = 0.33 μM for **12j** and 0.92 μM for **12i**. However, the displacement of the phenyl nucleus in position 8 of these disubstituted 3-(4-methylpiperazin-1-yl)propyl)aminobenzyl and 4-(4-methylpiperazin-1-yl)butyl)aminobenzylquinazolines (compounds **12m** and **12n**) did not lead to the same conclusion as SAR, derivative **12m** being more active than **12n** (IC_50_ = 0.88 μM for **12m** and 1.89 μM for **12n**). Concerning the quinoline compounds, **13a** was slightly more active than its other substituted homologs with an IC_50_ = 0.99 μM versus 1.07–1.76 μM.

For the MCF-7 breast adenocarcinoma, the introduction of the phenyl ring in position 6 or 7 of bis(3-dimethylaminopropyl)aminomethylphenylquinazolines (compounds **12b** and **12f**) was twice as active on this MCF-7 line than substitution of this aromatic nucleus in position 5 or 8 (compounds **12a** and **12k**) with IC_50_ of 0.58 μM for **12b** and **12f**, then ranging from 1.12 to 1.24 μM for **12a** and **12k**. The best antiproliferative activity was observed for quinoline **13a** with an IC_50_ of 0.54 μM. Moreover, the chain elongation with one additional carbon atom (compound **13b**) resulted in a decrease in the biological activity (IC_50_ = 3.03 μM). Similar observations were also made between compounds **12m** and **12n** (IC_50_ = 1.22 μM for **12m** versus 4.17 μM for **12n**). 

Among the nineteen compounds tested for antiproliferative activities on the breast cancer MDA-MB-231 cell line, 7-phenyl substituted quinazoline **12i** bearing 3-(4-methylpiperazin-1-yl)propyl)aminobenzyl side chains was the most active compound with an IC_50_ of 0.52 µM. From a general point of view, the 8-phenyl substituted quinazolines **12k**–**n** were less active against this breast cancer cell line (IC_50_ ranging from 1.36 to 3.94 μM) than their 5-phenyl, 6-phenyl, and 7-phenyl analogues, compounds **12a** (IC_50_ = 1.451 μM), **12b**–**e** (IC_50_ ranging from 0.70 to 1.44 μM) and **12f**–**j** (IC_50_ ranging from 0.52 to 1.10 μM), respectively. The bio-isosteric replacement of the quinazoline moiety by a quinoline displayed no benefit in terms of antiproliferative activity against the MDA-MB-231 cell line, except for derivative **13a** that was relatively active, with an IC_50_ of 0.58 μM.

Against the ovarian A2780 cancer cell line, the bisaminoalkylaminobenzyl substituted quinazolines **12f**–**j**, bearing the phenyl nucleus in position 7 of the heterocyclic skeleton, presented better antiproliferative activities than their 5-, 6-, or 8-phenyl analogues; i.e., IC_50_ = 0.79–1.74 μM for **12f**–**j** versus 1.20–7.10 μM for **12a**–**e** and **12k**–**n**. In addition, disubtituted quinoline **13a** with an IC_50_ of 0.90 μM was found 7.2 more active than its quinoline homolog **13b** bearing only one additional carbon atom in the diaminoalkyl side chains (IC_50_ = 6.49 μM).

The antiproliferative properties of these new derivatives **12**,**13** were also examined using the human myeloid leukemia cell line K562. The disubstituted quinazolines and quinolines showed significant antiproliferative activity, with IC_50_ values ranging from 7 to 34 μM. From a general point of view, compounds **12k**–**n**, substituted in position 8 of the quinazoline moiety by a phenyl, had the best pharmacological activity against the K562 cell line with an IC_50_ ranging from 7 to 14 μM. 

The most bioactive compounds were then tested on the human epithelial cell line HEK293 to evaluate their cytotoxicity on normal cells. Compounds **12b**, **12f**, **12i**, and **13a** displayed a low cytotoxicity against this HEK293 cell line (IC_50_ > 30 μM), while derivatives **12g** and **12j** were moderately cytotoxic at 3.98 and 4.90 μM, respectively. The index of selectivity (IS) was defined as the ratio of the IC_50_ value of the normal cell line HEK293 to the IC_50_ value of the different cancer cell lines (Table 2). Derivative **13a** displayed a promising index of selectivity towards the HeLa cell line (IS > 60) and breast adenocarcinoma MCF-7 cells (IS > 55.5). Quinazoline **12f** demonstrated an interesting IS towards the cervical SiHa cancer cell line (IS > 40), as well as the ovarian A2780 cancer cell line (IS > 38). Moreover, derivative **12i** showed interesting selectivity towards the breast cancer MDA-MB-231 cell line (SI > 57.7). 

**Figure 5:**
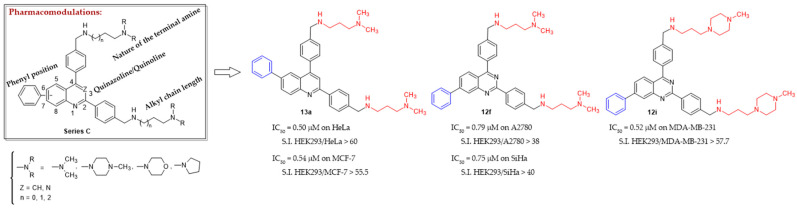
Structure–activity relationship analysis of quinazoline and quinoline derivatives **12,13**.

Consequently, comparing the two heterocyclic skeletons, we found no crucial differences between the corresponding analogs (**12b**–**13a**, **12d**–**13c**, **12k**–**13e**) and phenylquinazolines seemed more effective than their phenylquinoline analogs (**12c**–**13b**, **12e**–**13d**). Substituents containing dimethylamino functions were generally favored over methylpiperazin analogs (e.g., **12b**–**d**, **12l**–**n**, **13a**–**c**) except for **13d**, which was more active than **13b**. At the level of the position of the phenyl group, position 7 was more advantageous than position 6 (**12c**–**g**, **12d**–**i**, **12e**–**j**), while phenyl at position 5 or 8 resulted in less active molecules (e.g., **12a**, **12m**, **12n**, **13e**). The linker length between the two lateral chains (the n in Scheme 1) had limited action on the calculated IC_50_ values. However, a shorter linker (n = 1) tended to result in analogs with more pronounced cell growth-inhibiting action (**12b**, **12c**, **12m**, **12n**, **13a**–**d**). Collectively, the most promising compounds, i.e., **13a**, **12f**, **12b**, **12i**, **12g**, and **12j**, exhibited nanomolar or low micromolar IC_50_ values on the used adherent cell panel. Therefore, among these molecules, compounds **13a**, **12f**, and **12i** (Figure 5) may be regarded as drug candidates and their further development, including mechanistic cell-based studies, is highly advocated.

### 2.3. FRET-Melting Experiments

G4-forming motifs have previously been identified in oncogene promoter regions, bringing to light the therapeutic potential for targeted gene regulation at the transcriptional level. Among them, the most studied oncogene G4 promoters include c-MYC, K-RAS, BCL-2, h-Ras, and c-KIT. We thus decided to evaluate ligand-induced stabilization, specificity, and selectivity of these newly synthesized quinazolines **12a**–**n** and quinolines **13a**–**e** for various oncogene promoter G4 topologies including c-MYC, BCL-2, and K-RAS through FRET-melting experiments (Table 3, Figure S1).

The stabilization of our new derivatives was also investigated on the fluorescently labeled human telomeric sequence F21T. To probe the G4 selectivity of our novel heterocyclic compounds **12**,**13** over duplex DNA, a FRET-melting assay was performed using a duplex control sequence, FdxT. For comparison, the G4 ligand PhenDC3 was used as a reference compound. To compare levels of selectivity, the difference of melting temperature (ΔT_m_) between the T_m_ of the G-quadruplex formed by c-MYC, BCL-2, K-RAS, F21T, or FdxT in the presence or absence of each selected compound was calculated. For these novel disubstituted quinazoline and quinoline derivatives **12**,**13**, the ΔT_m_ values for the c-MYC, BCL-2, K-RAS, F21T sequences ranged from 5.0 to 33.9 °C at a 2 µM ligand concentration.

The best nitrogen heterocyclic ligands which stabilized the three pro-oncogene (c-MYC, BCL-2, K-RAS) and also the human F21T G-quadruplexes sequences (Table 4) were derivatives **12c**, **12e**, and **12h**, bearing substituted alkylaminobutylaminobenzyl or alkylaminopentylaminobenzyl side chains at positions 2 and 4 of the heterocyclic moiety, and mainly compound **12e** that showed the highest ΔT_m_ values (ΔT_m_ = 19.5, 33.1, 22.7 and 33.9 °C, respectively on the different c-MYC, K-RAS, BCL-2, and F21T sequences). Thus, the stabilization effects on the various quadruplex sequences seem to be dependent on the length of the alkylaminoalkylaminobenzyl side chains of the quinazolines **12**. 

We also observed that quinazoline compounds **12a**, **12b**, **12f**, and **12k** (all bearing two (3-dimethylaminopropyl)aminomethylphenyl side chains and a phenyl ring linked to the heterocyclic skeleton in the various positions 5, 6, 7 and 8, respectively) displayed lower stabilization than their other substituted quinazolinyl analogs **1**: ΔT_m_ = 5.8–9.9 °C on c-MYC, 10.6–20.1 °C on K-RAS, 7.4–12.6 °C on BCL-2 and 6.8–23.7 °C on F21T for **12a**, **12b**, **12f** and **12k** versus 8.4–19.5 °C on c-MYC, 15.5–33.1 °C on K-RAS, 9.5–22.7 °C on BCL-2 and 14.5–33.9 °C on F21T for the other quinazoline derivatives **12**. 

For each G-quadruplex sequence, the disubstituted quinoline ligands **13a**–**e** exhibited lower stabilization profiles in comparison with their quinazoline disubstituted homologs **12a**–**n**; these latter being better stabilizing ligands. 

Concerning the position of the substitution of the phenyl group on the nitrogen heterocycle, we cannot draw conclusions on their structure–activity relationships. In addition, quinazolines **12** and quinolines **13** were no more specific for the hybrid G4 topology of F21T sequence than for the parallel G4 structures of c-MYC, BCL-2, or K-RAS.

Hence, FRET assays showed that there was no stabilization of our heterocyclic structures on duplex DNA sequences.

### 2.4. Native Electrospray Mass Spectrometry

**Figure 6:**
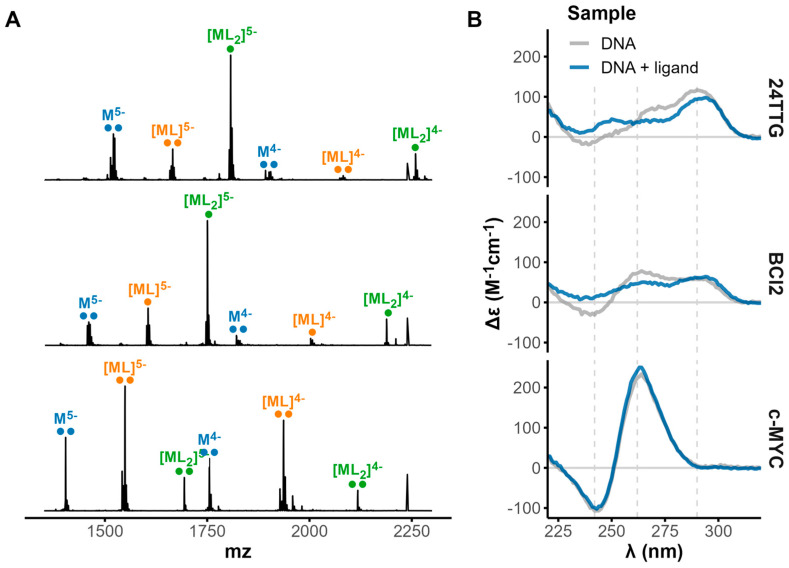
(**A**) Native electrospray mass spectra of **12e** (20 µM) and 24TTG, BCl-2, and c-MYC (10 µM) in 100 mM ammonium acetate (pH = 6.8), highlighting the 4- and 5- charge states: M = oligonucleotide monomer, L = ligand. The most abundant ammonium stoichiometry is labelled with • (1 NH_4_^+^) and •• (2 NH_4_^+^). (**B**) CD spectra of the same solutions (DNA + ligand; blue) compared to a reference with DNA alone (grey). The positions of characteristic bands at around 240, 260, and 290 nm are represented with dashed grey lines.

The binding affinity and stoichiometry of quinazoline (**12b**, **12e**) and quinoline (**13a**, **13d**) derivatives for G-quadruplexes were determined by native electrospray mass spectrometry [33]. We selected three G-quadruplex-forming oligonucleotides: the oncogene promoters c-MYC (5′-TGAG_3_TG_3_TAG_3_TG_3_TA_2_) and BCL-2 (5′-G_3_CGCG_3_AG_2_A_2_T_2_G_3_CG_3_) and the human telomeric sequence 24TTG (5′-T_2_(G_3_T_2_A)_3_G_3_A). Circular dichroism (CD) experiments revealed that in the 100 mM ammonium acetate buffer used for native MS experiments, c-MYC forms a parallel G-quadruplex (positive band at 260 nm, negative band at 240 nm; Figure 6 and Figure S2), whereas BCL-2 and 24TTG had hybrid or mixed signatures (positive bands at 290 and shoulder at 270 nm). Native MS confirms the formation of 3-tetrad G-quadruplexes specifically binding two ammonium cations (Figure 6). ds26, an oligonucleotide forming a hairpin duplex (CA_2_TCG_2_ATCGA_2_T_2_CGATC_2_GAT_2_G) was used as a control. 

The four ligands form complexes with all three G-quadruplexes with both 1:1 and 2:1 ligand:DNA stoichiometries (Figures S3–S5). The dissociation constants of the 1:1 complexes (*K*d_1_) formed by **12b**, **12e**, and **13a** with BCL-2 and 24TTG were in the micromolar range, whereas **13d** was a weaker binder by an order of magnitude (Table 5). The Kd_2_ of the second binding reaction of **12b**, **12e**, and **13a** was also in the micromolar range and in most cases lower. Remarkably, the second binding event was cooperative for **12b** and **12e** binding 24TTG (*K*d_1_/*K*d_2_ > 4) [34]. This suggests that the conformation of 24TTG and BCL-2 may be altered upon binding of these ligands to accommodate the second ligand more tightly. This is supported by (i) the change in the CD signature towards a more antiparallel signature (decrease in the shoulder at 270 nm; Figure 6B) and (ii) the displacement of an ammonium cation (particularly for 2:1 complexes). A decrease in cation stoichiometry may be the result of a switch towards two-tetrad conformers [35] or the intercalation of the ligand [36]. 

Binding of **12b**, **12e**, and **13a** to the parallel c-MYC G-quadruplex was similar in magnitude to that of 24TTG and BCL-2, but mostly restricted to 1:1 complexes (*K*d_1_~4–6 µM vs. *K*d_2_~36–100 µM). Consistently, there was no significant change in ammonium stoichiometry or CD signature. Again, **13d** was a weaker binder than its counterparts.

Finally, all four ligands bound moderately to the duplex control ds26 (Figure S6). The lack of stabilization of FdxT in the FRET-melting experiments could be explained by (i) the use of much lower oligonucleotide concentrations (0.2 µM vs. 10 µM here), leading to low binding resulting in large *K*_d_ values, and (ii) the difference in GC content between FdxT (20% GC) and ds26 (46% GC), the latter favoring intercalation of small molecules compared to the former.

### 2.5. Detection of Telomerase Activity in Cell Lysates

**Figure 7:**
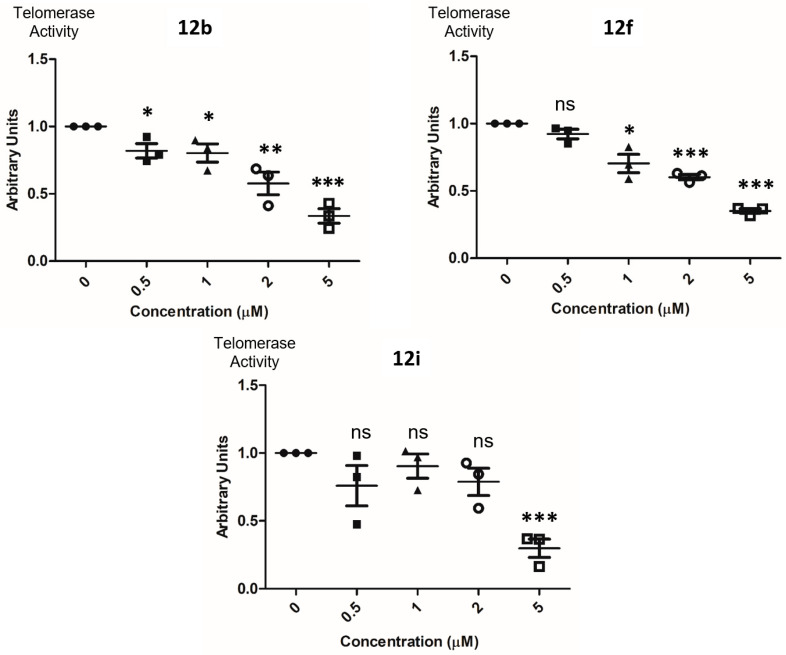
Telomerase activity in MCF-7 cell lysates was investigated in the presence of 0 to 5 μM of **12b**, **12f** and **12i** compounds. Error bars indicate standard deviation. ns: not statistically significant, *: *p* < 0.05, **: *p* < 0.01, ***: *p* < 0.001, comparing 0 mM of compound (control) to the presence of **12b**, **12f,** and **12i** compounds at concentrations between 0.5 to 5 μM.

Additionally, the more bioactive ligands **12b**, **12f**, and **12i** were also tested for telomerase activity in MCF-7 protein extracts. For MCF-7 cell lysate, we observed that 5 µM of **12b**, **12f**, or **12i** reduced drastically the telomerase activity compared to control. In our experimental conditions, MCF-7 cell lysates were more sensitive to **12b** than **12i**. Indeed, while compound **12b** decreased MCF-7 telomerase activity from 0.5 µM, for compound **12i** it was necessary to use 5 µM to drastically reduce MCF-7 telomerase activity (Figure 7). 

## 3. Materials and Methods

### 3.1. Chemistry

Commercially available reagents were used without additional purification. Melting points were determined with an SM-LUX-POL Leitz hot-stage microscope (Leitz GMBH, Midland, ON, USA) and are uncorrected. 

IR spectra were recorded on a NICOLET 380FT-IR spectrophotometer (Bruker BioSpin, Wissembourg, France). NMR spectra were recorded with tetramethylsilane as an internal standard using a BRUKER AVANCE 300 spectrometer (Bruker BioSpin, Wissembourg, France). Splitting patterns have been reported as follows: s = singlet; bs = broad singlet; d = doublet; t = triplet; q = quartet; dd = double doublet; ddd = double double doublet; qt = quintuplet; and m = multiplet. 

Analytical TLC were carried out on 0.25 precoated silica gel plates (POLYGRAM SIL G/UV254) (Merck KGaA, Darmstadt, Germany) and compounds were visualized after UV light irradiation. A silica gel 60 (70–230 mesh) was used for column chromatography. Mass spectra were recorded on an ESI LTQ Orbitrap Velos mass spectrometer (ThermoFisher, Bremen, Germany). Ionization was performed using an Electrospray ion source operating in positive ion mode with a capillary voltage of 3.80 kV and capillary temperature of 250 °C. The scan type analyzed was full scan, all MS recordings were in the *m*/*z* range between 150 to 2000 *m*/*z*. No fragmentation was carried out and the resolution used for the analysis was 60,000.

***General procedure for generating 2,4-bis(4-formylphenyl)-phenylquinazolines 8a–d and 2,4-bis(4-formylphenyl)-phenylquinolines* 9a,b
**

To a solution of 4.37 mmol of the appropriate 2,4-dichloroquinazoline **6a**–**d** or 2,4-dichloroquinoline **7a**,**b** 1.44 g of 3- or 4-formylphenyl boronic acid (9.63 mmol, 2.2 eq.) and 506 mg (0.437 mmol, 0.1 eq.) of tetrakis(triphenylphosphine)palladium(0) in 45 mL of 1,2-dimethoxyethane, 5 mL of 2 M K_2_CO_3_ aqueous solution, previously degassed for 10 min with nitrogen, were added at room temperature. The mixture was then warmed to reflux and stirred for 24 h under nitrogen-positive pressure. The reaction mix was cooled down to room temperature and the solvent was evaporated under *vacuum*. The organic layer was extracted with CH_2_Cl_2_, and the organic phase was filtered on filter paper, then washed with water (20 mL × 3 times), dried over anhydrous sodium sulfate and activated charcoal, filtered and evaporated under *vacuum*. The residue was cooled and triturated with a minimum of EtOH and EtO_2_ and filtered on sintered glassware to obtain the crude product. The residue was purified using silica gel column chromatography (CH_2_Cl_2_/CH_3_OH 95:5), then cooled and triturated in EtOH, filtered on sintered glassware, washed with a minimum of EtOH, EtO_2_, and petroleum ether, and dried under pressure to generate the solid product **8**,**9**.

***2,4-bis(4-formylphenyl)-5-phenylquinazoline* (8a)**

Pale yellow crystals (39%); m.p. = 214–216 °C; ^1^H NMR (CDCl_3_) *δ* ppm: 10.16 (s, 1H, CHO), 9.94 (s, 1H, CHO), 8.90 (d, 2H, *J* = 8.40 Hz, H-3″, and H-5″), 8.28 (dd, 1H, *J* = 8.40, and 1.20 Hz, H-8), 8.08 (d, 2H, *J* = 8.40 Hz, H-3′, and H-5′), 8.04 (dd, 1H, *J* = 8.40, and 7.20 Hz, H-7), 7.68 (dd, 1H, *J* = 7.20, and 1.50 Hz, H-6), 7.61 (d, 2H, *J* = 8.40 Hz, H-2″, and H-6″), 7.53 (d, 2H, *J* = 8.40 Hz, H-2′, and H-6′), 7.12–6.95 (m, 5H, H-phe).

***2,4-bis(4-formylphenyl)-6-phenylquinazoline* (8b)**

Yellow crystals (41%); m.p. = 221–223 °C; ^1^H NMR (CDCl_3_) *δ* ppm: 10.22 (s, 1H, CHO), 10.16 (s, 1H, CHO), 8.90 (d, 2H, *J* = 8.10 Hz, H-3″, and H-5″), 8.32 (d, 1H, *J* = 7.80 Hz, H-8), 8.26 (dd, 1H, *J* = 7.80, and 2.10 Hz, H-7), 8.24 (d, 1H, *J* = 2.10 Hz, H-5), 8.18 (d, 2H, *J* = 8.10 Hz, H-3′, and H-5′), 8.12 (d, 2H, *J* = 8.10 Hz, H-2″, and H-6″), 8.08 (d, 2H, *J* = 8.10 Hz, H-2′, and H-6′), 7.66 (d, 2H, *J* = 7.00 Hz, H-2, and H-6_phe_), 7.52 (t, 2H, *J* = 7.00 Hz, H-3, and H-5_phe_), 7.46 (t, 1H, *J* = 7.00 Hz, H-4_phe_).

***2,4-bis(4-formylphenyl)-7-phenylquinazoline* (8c)**

Pale-yellow crystals (51%); m.p. = 202–204 °C; ^1^H NMR (CDCl_3_) *δ* ppm: 10.23 (s, 1H, CHO), 10.17 (s, 1H, CHO), 8.90 (d, 2H, *J* = 8.20 Hz, H-3″, and H-5″), 8.46 (d, 1H, *J* = 1.60 Hz, H-8), 8.20 (d, 2H, *J* = 8.40, H-3′, and H-5′), 8.19 (d, 1H, *J* = 8.70 Hz, H-5), 8.11 (d, 2H, *J* = 8.20 Hz, H-2″, and H-6″), 8.08 (d, 2H, *J* = 8.20 Hz, H-2′, and H-6′), 7.93 (dd, 1H, *J* = 8.70 Hz, and *J* = 1.60 Hz, H-6), 7.86–7.82 (d, 2H, *J* = 7.40 Hz, H-2, and H-6_phe_), 7.58 (t, 2H, *J* = 7.40 Hz, H-3, and H-5_phe_), 7.50 (t, 1H, *J* = 7.40 Hz, H-4_phe_). ^13^C NMR (CDCl_3_) *δ* ppm: 192.3 (CHO), 191.8 (CHO), 166.9 (C-2), 159.4 (C-4), 152.4 (C-8a), 149.6 (C-1″), 143.4 (C-7), 143.0 (C-1_phe_), 139.1 (C-1′), 137.6 (C-4′), 137.2 (C-4″), 130.8 (C-3″ and C-5″), 130.0 (C-3′ and C-5′), 129.9 (C-2′ and C-6′), 129.3 (C-3, C-5_phe_ and C-4_phe_), 129.2 (C-2″ and C-6″), 128.9 (C-4_phe_), 127.9 (C-8), 127.6 (C-2 and C-6_phe_), 126.9 (C-5), 126.8 (C-6), 120.7 (C-4a).

***2,4-bis(4-formylphenyl)-8-phenylquinazoline* (8d)**

Pale-yellow crystals (51%); m.p. = 212–214 °C; ^1^H NMR (CDCl_3_) *δ* ppm: 10.22 (s, 1H, CHO), 10.11 (s, 1H, CHO), 8.76 (d, 2H, *J* = 8.40 Hz, H-3″, and H-5″), 8.16 (d, 2H, *J* = 8.40 Hz, H-3′, and H-5′), 8.09 (d, 2H, *J* = 8.40 Hz, H-2″, and H-6″), 8.07 (dd, 1H, *J* =7.80, and 1.20 Hz, H-7), 8.05 (dd, 1H, *J* = 7.35, and 1.20 Hz, H-5), 8.01(d, 2H, *J* = 8.40 Hz, H-2′, and H-6′), 7.88 (d, 2H, *J* = 8.10 Hz, H-2_phe,_ and H-6_phe_), 7.71 (dd, 1H, *J* =7.80 H,z, and *J* = 7.35 Hz, H-6), 7.62 (t, 2H, *J* = 8.10 Hz, H-3, and H-5_phe_),7.56 (t, 1H, *J* = 8.10 Hz, H-4_phe_).

***2,4-bis(4-formylphenyl)-6-phenylquinoline* (9a)**

Pale-orange crystals (66%); m.p. = 207–209 °C; ^1^H NMR (CDCl_3_) *δ* ppm: 10.19 (s, 1H, CHO), 10.15 (s, 1H, CHO), 8.43 (d, 2H, *J* = 8.25 Hz, H-3″, and H-5″), 8.37 (d, 1H, *J* = 8.70 Hz, H-8), 8.13 (d, 2H, *J* = 8.25 Hz, H-3′, and H-5′), 8.08 (dd, 1H, *J* = 8.70, and 1.80 Hz, H-7), 8.07 (d, 2H, *J* = 8.25 Hz, H-2″, and H6″), 8.02 (d, 1H, *J* = 1.80 Hz, H-5), 7.92 (s, 1H, H-3), 7.82 (d, 2H, *J* = 8.25 Hz, H-2′, and H-6′), 7.64 (d, 2H, *J* = 7.20 Hz, H-2, and H-6_phe_), 7.48 (t, 2H, *J* = 7.20 Hz, H-3, and H-5_phe_),7.41 (t, 1H, *J* = 7.20 Hz, H-4_phe_).

***2,4-bis(4-formylphenyl)-8-phenylquinoline* (9b)**

Pale-yellow crystals (27%); m.p. = 127–129 °C; ^1^H NMR (CDCl_3_) *δ* ppm: 10.20 (s, 1H, CHO), 10.01 (s, 1H, CHO), 8.34 (d, 2H, *J* = 8.25 Hz, H-3″, and H-5″), 8.12 (d, 2H, *J* = 8.25 Hz, H-3′, and H-5′), 8.01 (d, 2H, *J* = 8.25 Hz, H-2″, and H6″), 7.98 (dd, 1H, *J* =7.80, and 1.25 Hz, H-7), 7.96 (dd, 1H, *J* = 7.70, and 1.25 Hz, H-5), 7.93 (s, 1H, H-3), 7.88–7.76 (m, 5H, H-2′, H-6′, H-6, H-2_phe_, and H-6_phe_), 7.65–7.50 (m, 3H, H-3, H-4, and H-5_phe_).

***General procedure for 2,4-bis[(substituted-iminomethyl)phenyl]-phenylquinazolines 10a–n and 2,4-bis[(substituted-iminomethyl)phenyl]-phenylquinolines* 11a–e
**

To a solution of diamine (0.126 mmol, 2.1 eq.) in ethanol (7 mL), we added 2,4-bis(4-formylphenyl)quinazoline **8** or 1,3-bis(4-formylphenyl)quinoline **9** (0.6 mmol). The reaction mix was then heated under reflux for 5 h and evaporated to dryness under reduced pressure. After cooling, the residue was extracted with dichloromethane (40 mL). The organic layer was dried over sodium sulfate and activated charcoal and evaporated to dryness. Products were then used without further purification.

***2,4-bis{4-[(3-dimethylaminopropyl)iminomethyl]phenyl}-5-phenylquinazoline* (10a)**

Yellow oil (98%); ^1^H NMR (CDCl_3_) *δ* ppm: 8.72 (d, 2H, *J* = 8.40 Hz, H-3″, and H-5″), 8.34 (s, 1H, CH=N), 8.13 (dd, 1H, *J* = 8.40, and 1.20 Hz, H-8), 8.09 (s, 1H, CH=N), 7.86 (dd, 1H, *J* = 8.40, and 7.20 Hz, H-7), 7.84 (d, 2H, *J* = 8.40 Hz, H-3′, and H-5′), 7.52 (dd, 1H, *J* = 7.20, and 1.50 Hz, H-6), 7.35–7.33 (m, 4H, H-2″, H-6″, H-2′, and H-6′), 7.03–6.93 (m, 5H, H-phe), 3.65 (t, 2H, *J* = 7.20 Hz, NCH_2_), 3.60 (t, 2H, *J* = 7.20 Hz, NCH_2_), 2.34 (t, 2H, *J* = 7.20 Hz, NCH_2_), 2.30 (t, 2H, *J* = 7.20 Hz, NCH_2_), 2.21 (s, 6H, N(CH_3_)_2_), 2.20 (s, 6H, N(CH_3_)_2_), 1.91–1.80 (m, 4H, 2CH_2_).

***2,4-bis{4-[(3-dimethylaminopropyl)iminomethyl]phenyl}-6-phenylquinazoline* (10b)**

Yellow crystals (97%); m.p. = 116–118 °C; ^1^H NMR (CDCl_3_) *δ* ppm: 8.77 (d, 2H, *J* = 8.40 Hz, H-3″, and H-5″), 8.46 (s, 1H, CH=N), 8.40 (s, 1H, CH=N), 8.29 (d, 1H, *J* = 1.95 Hz, H-5), 8.24 (d, 1H, *J* = 8.40 Hz, H-8), 8.18 (dd, 1H, *J* = 8.40, and 1.95 Hz, H-7), 8.03–7.98 (m, 4H, H-3′,H-5′, H-2″, and H-6″), 7.90 (d, 2H, *J* = 8.40 Hz, H-2′, and H-6′), 7.66 (d, 2H, *J* = 8.40 Hz, H-2, and H-6_phe_), 7.51 (t, 2H, *J* = 8.40 Hz, H-3, and H-5_phe_), 7.43 (t, 1H, *J* = 8.40 Hz, H-4_phe_), 3.74 (t, 2H, *J* = 7.05 Hz, NCH_2_), 3.72 (t, 2H, *J* = 7.05 Hz, NCH_2_), 2.43 (t, 2H, *J* = 7.05 Hz, NCH_2_), 2.40 (t, 2H, *J* = 7.05 Hz, NCH_2_), 2.28 (s, 6H, N(CH_3_)_2_), 2.27 (s, 6H, N(CH_3_)_2_), 1.99–1.87 (m, 4H, 2CH_2_).

***2,4-bis{4-[(4-dimethylaminobutyl)iminomethyl]phenyl}-6-phenylquinazoline* (10c)**

Orange oil (98%); ^1^H NMR (CDCl_3_) *δ* ppm: 8.72 (d, 2H, *J* = 8.70 Hz, H-3″ and H-5″), 8.39 (s, 1H, CH=N), 8.34 (s, 1H, CH=N), 8.23 (d, 1H, *J* = 1.80 Hz, H-5), 8.18 (d, 1H, *J* = 8.70 Hz, H-8), 8.12 (dd, 1H, *J* = 8.70, and 1.80 Hz, H-7), 7.96–7.93 (m, 4H, H-3′,H-5′, H-2″, and H-6″), 7.85 (d, 2H, *J* = 8.70 Hz, H-2′, and H-6′), 7.59 (d, 2H, *J* = 7.50 Hz, H-2, and H-6_phe_),7.44 (t, 2H, *J* = 7.50 Hz, H-3, and H-5_phe_), 7.36 (t, 1H, *J* = 7.50 Hz, H-4_phe_), 3.68 (t, 2H, *J* = 6.90 Hz, NCH_2_), 3.64 (t, 2H, *J* = 6.90 Hz, NCH_2_), 2.32 (t, 2H, *J* = 6.90 Hz, NCH_2_), 2.28 (t, 2H, *J* = 6.90 Hz, NCH_2_), 2.22 (s, 6H, N(CH_3_)_2_), 2.21 (s, 6H, N(CH_3_)_2_), 1.80–1.69 (m, 4H, 2CH_2_), 1.60–1.52 (m, 4H, 2CH_2_).

***2,4-bis{4-[(3-(4-methylpiperazin-1-yl)propyl)iminomethyl]phenyl}-6-phenylquinazoline* (10d)**

Yellow crystals (98%); m.p. = 129–131 °C; ^1^H NMR (CDCl_3_) *δ* ppm: 8.66 (d, 2H, *J* = 8.10 Hz, H-3″ and H-5″), 8.34 (s, 1H, CH=N), 8.27 (s, 1H, CH=N), 8.16 (d, 1H, *J* = 1.80 Hz, H-5), 8.11 (d, 1H, *J* = 8.20 Hz, H-8), 8.04 (dd, 1H, *J* = 8.70, and 1.80 Hz, H-7), 7.90–7.86 (m, 4H, H-3′,H-5′, H-2″, and H-6″), 7.79 (d, 2H, *J* = 8.10 Hz, H-2′, and H-6′), 7.52 (d, 2H, *J* = 8.10 Hz, H-2, and H-6_phe_), 7.37 (t, 2H, *J* = 8.10 Hz, H-3, and H-5_phe_) 7.30 (t, 1H, *J* = 8.10 Hz, H-4_phe_), 3.65 (t, 2H, *J* = 6.90 Hz, NCH_2_), 3.61 (t, 2H, *J* = 6.90 Hz, NCH_2_), 2.52–2.30 (m, 20H, 2NCH_2,_ and 8NCH_2pip_), 2.22 (s, 6H, 2NCH_3_), 1.90–1.86 (m, 4H, 2CH_2_).

***2,4-bis{4-[(4-(4-methylpiperazin-1-yl)butyl)iminomethyl]phenyl}-6-phenylquinazoline* (10e)**

Yellow-orange oil (57%); ^1^H NMR (CDCl_3_) *δ* ppm: 8.72 (d, 2H, *J* = 8.25 Hz, H-3″, and H-5″), 8.38 (s, 1H, CH=N), 8.32 (s, 1H, CH=N), 8.22 (d, 1H, *J* = 1.80 Hz, H-5), 8.18 (d, 1H, *J* = 8.75 Hz, H-8), 8.12 (dd, 1H, *J* = 8.75, and 1.80 Hz, H-7), 7.97–7.90 (m, 4H, H-3′, H-5′, H-2″, and H-6″), 7.84 (d, 2H, *J* = 8.25 Hz, H-2′, and H-6′), 7.58 (d, 2H, *J* = 8.40 Hz, H-2, and H-6_phe_), 7.43 (t, 2H, *J* = 8.40 Hz, H-3, and H-5_phe_), 7.34 (t, 1H, *J* = 8.40 Hz, H-4_phe_), 3.70–3.61 (m, 4H, 2NCH_2_), 3.65–2.32 (m, 20H, 2 NCH_2,_ and 8 NCH_2pip_), 2.24 (s, 6H, 2NCH_3_), 1.78–1.70 (m, 4H, 2CH_2_), 1.61–1.55 (m, 4H, 2CH_2_).

***2,4-bis{4-[(3-dimethylaminopropyl)iminomethyl]phenyl}-7-phenylquinazoline* (10f)**

Yellow oil (98%); ^1^H NMR (CDCl_3_) *δ* ppm: 8.71 (d, 2H, *J* = 8.40 Hz, H-3″, and H-5″), 8.38 (s, 1H, CH=N), 8.32 (s, 1H, CH=N), 8.28 (d, 1H, *J* = 1.50 Hz, H-8), 8.05 (d, 1H, *J* = 8.70 Hz, H-5), 7. 92 (d, 2H, *J* = 8.40 Hz, H-3′, and H-5′), 7.89 (d, 2H, *J* = 8.40 Hz, H-2″ and H-6″), 7.83 (d, 2H, *J* = 8.40 Hz, H-2′, and H-6′), 7.77 (dd, 1H, *J* = 8.70, and 1.50 Hz, H-6), 7.72 (d, 2H, *J* = 7.50 Hz, H-2, and H-6_phe_), 7.46 (t, 2H, *J* = 7.50 Hz, H-3, and H-5_phe_), 7.38 (t, 1H, *J* = 7.50 Hz, H-4_phe_), 3.69 (t, 2H, *J* = 6.90 Hz, NCH_2_), 3.66 (t, 2H, *J* = 6.90 Hz, NCH_2_), 2.37 (t, 2H, *J* = 6.90 Hz, NCH_2_), 2.35 (t, 2H, *J* = 6.90 Hz, NCH_2_), 2.24 (s, 6H, N(CH_3_)_2_), 2.23 (s, 6H, N(CH_3_)_2_), 1.97–1.83 (m, 4H, 2CH_2_).

***2,4-bis{4-[(4-dimethylaminobutyl)iminomethyl]phenyl}-7-phenylquinazoline* (10g)**

Yellow oil (98%); ^1^H NMR (CDCl_3_) *δ* ppm: 8.73 (d, 2H, *J* = 8.40 Hz, H-3″, and H-5″), 8.40 (s, 1H, CH=N), 8.35 (s, 1H, CH=N), 8.33 (d, 1H, *J* = 1.65 Hz, H-8), 8.11 (d, 1H, *J* = 8.70 Hz, H-5), 7.95 (d, 2H, *J* = 8.40, H-3′, H-5′), 7.93 (d, 2H, *J* = 8.40, H-2″, and H-6″), 7.86 (d, 2H, *J* = 8.40 Hz, H-2′, and H-6′), 7.78 (dd, 1H, *J* = 8.70, and 1.65 Hz, H-6), 7.75 (d, 2H, *J* = 7.80 Hz, H-2, and H-6_phe_), 7.51 (t, 2H, *J* = 7.80 Hz, H-3, and H-5_phe_), 7.43 (t, 1H, *J* = 7.80 Hz, H-4_phe_), 3.70 (t, 2H, *J* = 6.90 Hz, NCH_2_), 3.67 (t, 2H, *J* = 6.90 Hz, NCH_2_), 2.36 (t, 2H, *J* = 6.90 Hz, NCH_2_), 2.32 (t, 2H, *J* = 6.90 Hz, NCH_2_), 2.25 (s, 6H, N(CH_3_)_2_), 2.23 (s, 6H, N(CH_3_)_2_), 1.82–1.70 (m, 4H, 2CH_2_), 1.65–1.54 (m, 4H, 2CH_2_).

***2,4-bis{4-[(5-dimethylaminopentyl)iminomethyl]phenyl}-7-phenylquinazoline* (10h)**

Orange oil (98%); ^1^H NMR (CDCl_3_) *δ* ppm: 8.72 (d, 2H, *J* = 8.40 Hz, H-3″, and H-5″), 8.37 (s, 1H, CH=N), 8.31 (s, 1H, CH=N), 8.30 (d, 1H, *J* = 1.80 Hz, H-8), 8.09 (d, 1H, *J* = 8.70 Hz, H-5), 7.93 (d, 2H, *J* = 8.40, H-3′, H-5′), 7.91 (d, 2H, *J* = 8.40, H-2″, and H-6″), 7.85 (d, 2H, *J* = 8.40 Hz, H-2′, and H-6′), 7.76 (dd, 1H, *J* = 8.70, and 1.80 Hz, H-6), 7.73 (d, 2H, *J* = 7.20 Hz, H-2, and H-6_phe_), 7.48 (t, 2H, *J* = 7.20 Hz, H-3, and H-5_phe_), 7.41 (t, 1H, *J* = 7.20 Hz, H-4_phe_), 3.67 (t, 2H, *J* = 6.90 Hz, NCH_2_), 3.63 (t, 2H, *J* = 6.90 Hz, NCH_2_), 2.31 (t, 2H, *J* = 6.90 Hz, NCH_2_), 2.28 (t, 2H, *J* = 6.90 Hz, NCH_2_), 2.23 (s, 6H, N(CH_3_)_2_), 2.22 (s, 6H, N(CH_3_)_2_), 1.80–1.72 (m, 4H, 2CH_2_), 1.56–1.45 (m, 4H, 2CH_2_), 1.43–1.35 (m, 4H, 2CH_2_).

***2,4-bis{4-[(3-(4-methylpiperazin-1-yl)propyl)iminomethyl]phenyl}-7-phenylquinazoline* (10i)**

Yellow oil (98%); ^1^H NMR (CDCl_3_) *δ* ppm: 8.69 (d, 2H, *J* = 8.40 Hz, H-3″, and H-5″), 8.36 (s, 1H, CH=N), 8.31 (s, 1H, CH=N), 8.29 (d, 1H, *J* = 1.50 Hz, H-8), 8.07 (d, 1H, *J* = 8.70 Hz, H-5), 7.91–7.85 (m, 4H, H-3′, H-5′, H-2″, and H-6″), 7.82 (d, 2H, *J* = 8.40 Hz, H-2′, and H-6′), 7.73 (dd, 1H, *J* = 8.70 and 1.50 Hz, H-6), 7.71 (d, 2H, *J* = 8.00 Hz, H-2, and H-6_phe_), 7.46 (t, 2H, *J* = 8.00 Hz, H-3, and H-5_phe_), 7.38 (t, 1H, *J* = 8.00 Hz, H-4_phe_), 3.67 (t, 2H, *J* = 6.90 Hz, NCH_2_), 3.63 (t, 2H, *J* = 6.90 Hz, NCH_2_), 2.56–2.31 (m, 20H, 2 NCH_2,_ and 8 NCH_2pip_), 2.24 (s, 3H, NCH_3_), 2.23 (s, 3H, NCH_3_), 1.95–1.83 (m, 4H, 2CH_2_).

***2,4-bis{4-[(4-(4-methylpiperazin-1-yl)butyl)iminomethyl]phenyl}-7-phenylquinazoline* (10j)**

Orange-yellow oil (98%); ^1^H NMR (CDCl_3_) *δ* ppm: 8.74 (d, 2H, *J* = 8.40 Hz, H-3″, and H-5″), 8.39 (s, 1H, CH=N), 8.35 (s, 1H, CH=N), 8.34 (d, 1H, *J* = 1.80 Hz, H-8), 8.13 (d, 1H, *J* = 8.70 Hz, H-5), 7.95–7.92 (m, 4H, H-3′,H-5′, H-2″, and H-6″), 7.86 (d, 2H, *J* = 8.40 Hz, H-2′, and H-6′), 7.80 (dd, 1H, *J* = 8.70, and 1.80 Hz, H-6), 7.77 (d, 2H, *J* = 7.50 Hz, H-2, and H-6_phe_), 7.51 (t, 2H, *J* = 7.50 Hz, H-3, and H-5_phe_), 7.43 (t, 1H, *J* = 7.50 Hz, H-4_phe_), 3.69 (t, 2H, *J* = 6.60 Hz, NCH_2_), 3.65 (t, 2H, *J* = 6.60 Hz, NCH_2_), 2.53–2.30 (m, 20H, 2 NCH_2,_ and 8 NCH_2pip_), 2.28 (s, 3H, NCH_3_), 2.27 (s, 3H, NCH_3_), 1.80–1.70 (m, 4H, 2CH_2_), 1.65–1.53 (m, 4H, 2CH_2_).

***2,4-bis{4-[(3-dimethylaminopropyl)iminomethyl]phenyl}-8-phenylquinazoline* (10k)**

Pale-yellow crystals (95%); m.p. = 117–119 °C; ^1^H NMR (CDCl_3_) *δ* ppm: 8.67 (d, 2H, *J* = 8.40 Hz, H-3″, and H-5″), 8.46 (s, 1H, CH=N), 8.37 (s, 1H, CH=N), 8.11 (dd, 1H, *J* = 8.40, and 1.50 Hz, H-7), 7.99 (dd, 1H, *J* = 8.40, and 1.50 Hz, H-5), 7.98 (d, 2H, *J* = 8.40 Hz, H-3′, and H-5′), 7.97 (d, 2H, *J* = 8.40 Hz, H-2″, and H-6″), 7.89 (d, 2H, *J* = 7.20 Hz, H-2, and H-6_phe_), 7.84 (d, 2H, *J* = 8.40 Hz, H-2′, and H-6′), 7.67–7.51 (m, 4H, H-6, H-3, H-4, and H-5_phe_), 3.76 (t, 2H, *J* = 6.90 Hz, NCH_2_), 3.70 (t, 2H, *J* = 6.90 Hz, NCH_2_), 2.42 (t, 2H, *J* = 6.90 Hz, NCH_2_), 2.39 (t, 2H, *J* = 6.90 Hz, NCH_2_), 2.29 (s, 6H, N(CH_3_)_2_), 2.27 (s, 6H, N(CH_3_)_2_), 2.01–1.87 (m, 4H, 2CH_2_).

***2,4-bis{4-[(4-dimethylaminobutyl)iminomethyl]phenyl}-8-phenylquinazoline* (10l)**

Pale-yellow crystals (98%); m.p. = 90–92 °C; ^1^H NMR (CDCl_3_) *δ* ppm: 8.61 (d, 2H, *J* = 8.10 Hz, H-3″, and H-5″), 8.40 (s, 1H, CH=N), 8.31 (s, 1H, CH=N), 8.06 (dd, 1H, *J* = 8.20, and 1.50 Hz, H-7), 7.96–7.90 (m, 5H, H-5, H-3′, H-5′, H-2″, and H-6″), 7.85 (d, 2H, *J* = 7.20, H-2, H-6_phe_), 7.78 (d, 2H, *J* = 8.10, H-2′, and H-6′), 7.59 (t, 1H, *J* = 8.10 Hz, H-6), 7.54 (t, 2H, *J* = 7.20 Hz, H-3, and H-5_phe_), 7.46 (t, 1H, *J* = 7.20 Hz, H-4_phe_), 3.69 (t, 2H, *J* = 5.40 Hz, NCH_2_), 3.63 (t, 2H, *J* = 5.40 Hz, NCH_2_), 2.32 (t, 2H, *J* = 5.40 Hz, NCH_2_), 2.29 (t, 2H, *J* = 5.40 Hz, NCH_2_), 2.22 (s, 6H, N(CH_3_)_2_), 2.20 (s, 6H, N(CH_3_)_2_), 1.80–1.70 (m, 4H, 2CH_2_), 1.61–1.49 (m, 4H, 2CH_2_).

***2,4-bis{4-[(3-(4-methylpiperazin-1-yl)propyl)iminomethyl]phenyl}-8-phenylquinazoline* (10m)**

Pale yellow oil (98%); ^1^H NMR (CDCl_3_) *δ* ppm: 8.61 (d, 2H, *J* = 8.10 Hz, H-3″, and H-5″), 8.41 (s, 1H, CH=N), 8.32 (s, 1H, CH=N), 8.07 (dd, 1H, *J* = 8.40, and 1.50 Hz, H-7), 7.95 (dd, 1H, *J* = 8.40, and 1.50 Hz, H-5), 7.94–7.92 (m, 4H, H-3′,H-5′, H-2″, and H-6″), 7.84 (d, 2H, *J* = 7.20 Hz, H-2, and H-6_phe_), 7.78 (d, 2H, *J* = 8.10 Hz, H-2′, and H-6′), 7.62–7.57 (m, 1H, H-6), 7.54 (t, 2H, *J* = 7.20 Hz, H-3, and H-5_phe_), 7.47 (t, 1H, *J* = 7.20 Hz, H-4_phe_), 3.71 (t, 2H, *J* = 5.10 Hz, NCH_2_), 3.65 (t, 2H, *J* = 5.10 Hz, NCH_2_), 2.61–2.38 (m, 20H, 2 NCH_2, and_ 8 NCH_2pip_), 2.28 (s, 3H, NCH_3_), 2.26 (s, 3H, NCH_3_), 1.98–1.86 (m, 4H, 2CH_2_).

***2,4-bis{4-[(4-(4-methylpiperazin-1-yl)butyl)iminomethyl]phenyl}-8-phenylquinazoline* (10n)**

Yellow oil (98%); ^1^H NMR (CDCl_3_) *δ* ppm: 8.61 (d, 2H, *J* = 8.40 Hz, H-3″, and H-5″), 8.38 (s, 1H, CH=N), 8.29 (s, 1H, CH=N), 8.05 (dd, 1H, *J* = 8.40, and 1.20 Hz, H-7), 7.94–7.89 (m, 5H, H-5, H-3′, H-5′, H-2″, and H-6″), 7.84 (d, 2H, *J* = 7.20 Hz, H-2, and H-6_phe_), 7.78 (d, 2H, *J* = 8.40 Hz, H-2′, and H-6′), 7.60–7.44 (m, 4H, H-3, H-6, H-4, and H-5_phe_), 3.68 (t, 2H, *J* = 7.20 Hz, NCH_2_), 3.63 (t, 2H, *J* = 7.20 Hz, NCH_2_), 2.65–2.29 (m, 20H, 2 NCH_2, and_ 8 NCH_2pip_), 2.27 (s, 3H, NCH_3_), 2.25 (s, 3H, NCH_3_), 1.79–1.68 (m, 4H, 2CH_2_), 1.61–1.50 (m, 4H, 2CH_2_).

***2,4-bis{4-[(3-dimethylaminopropyl)iminomethyl]phenyl}-6-phenylquinoline* (11a)**

Yellow-orange oil (98%); ^1^H NMR (CDCl_3_) *δ* ppm: 8.42 (s, 1H, CH=N), 8.38 (s, 1H, CH=N), 8.31 (d, 1H, *J* = 8.60 Hz, H-8), 8.28 (d, 2H, *J* = 8.40 Hz, H-3″, and H-5″), 8.08 (d, 1H, *J* = 2.00 Hz, H-5), 8.01 (dd, 1H, *J* = 8.60, and 2.00 Hz, H-7), 7.93 (d, 2H, *J* = 8.40 Hz, H-3′,H-5′), 7.89 (d, 2H, *J* = 8.40 Hz, H-2″, and H-6″), 7.87 (s, 1H, H-3), 7.66 (d, 2H, *J* = 8.40 Hz, H-2′, and H-6′), 7.62 (d, 2H, *J* = 7.50 Hz, H-2, and H-6_phe_), 7.44 (d, 2H, *J* = 7.50 Hz, H-3, and H-5_phe_), 7.38 (t, 1H, *J* = 7.50 Hz, H-4_phe_), 3.73 (t, 2H, *J* = 6.90 Hz, NCH_2_), 3.70 (t, 2H, *J* = 6.90 Hz, NCH_2_), 2.41 (t, 2H, *J* = 6.90 Hz, NCH_2_), 2.38 (t, 2H, *J* = 6.90 Hz, NCH_2_), 2.28 (s, 6H, N(CH_3_)_2_), 2.26 (s, 6H, N(CH_3_)_2_), 1.97–1.85 (m, 4H, 2CH_2_).

***2,4-bis{4-[(4-dimethylaminobutyl)iminomethyl]phenyl}-6-phenylquinoline* (11b)**

Pale-orange oil (98%); ^1^H NMR (CDCl_3_) *δ* ppm: 8.40 (s, 1H, CH=N), 8.35 (s, 1H, CH=N), 8.30 (d, 1H, *J* = 8.70 Hz, H-8), 8.27 (d, 2H, *J* = 8.10 Hz, H-3″, and H-5″), 8.07 (d, 1H, *J* = 1.80, H-5), 8.00 (dd, 1H, *J* = 8.70, and 1.80 Hz, H-7), 7.92 (d, 2H, *J* = 8.10 Hz, H-3′and H-5′), 7.88 (d, 2H, *J* = 8.10 Hz, H-2″, and H-6″), 7.86 (s, 1H, H-3), 7.64 (d, 2H, *J* = 8.10 Hz, H-2′, and H-6′), 7.61 (d, 2H, *J* = 7.80 Hz, H-2, and H-6_phe_), 7.44 (t, 2H, *J* = 7.80 Hz, H-3, and H-5_phe_), 7.32 (t, 1H, *J* = 7.80 Hz, H-4_phe_), 3.70 (t, 2H, *J* = 6.90 Hz, NCH_2_), 3.68 (t, 2H, *J* = 6.90 Hz, NCH_2_), 2.34 (t, 2H, *J* = 6.90 Hz, NCH_2_), 2.31 (t, 2H, *J* = 6.90 Hz, NCH_2_), 2.23 (s, 6H, N(CH_3_)_2_), 2.21 (s, 6H, N(CH_3_)_2_), 1.81–1.71 (m, 4H, 2CH_2_), 1.63–1.53 (m, 4H, 2CH_2_).

***2,4-bis{4-[(3-(4-methylpiperazin-1-yl)propyl)iminomethyl]phenyl}-6-phenylquinoline* (11c)**

Yellow oil (98%); ^1^H NMR (CDCl_3_) *δ* ppm: 8.41 (s, 1H, CH=N), 8.37 (s, 1H, CH=N), 8.31 (d, 1H, *J* = 8.70 Hz, H-8), 8.28 (d, 2H, *J* = 8.40 Hz, H-3″, and H-5″), 8.07 (d, 1H, *J* = 1.80 Hz, H-5), 8.01 (dd, 1H, *J* = 8.70, and 1.80 Hz, H-7), 7.92 (d, 2H, *J* = 8.40 Hz, H-3′, and H-5′), 7.88 (d, 2H, *J* = 8.40 Hz, H-2″, and H-6″), 7.87 (s, 1H, H-3), 7.66 (d, 2H, *J* = 8.40 Hz, H-2′, and H-6′), 7.61 (d, 2H, *J* = 7.20 Hz, H-2, and H-6_phe_), 7.45 (t, 2H, *J* = 7.20 Hz, H-3, and H-5_phe_), 7.36 (t, 1H, *J* = 7.20 Hz, H-4_phe_), 3.72 (t, 2H, *J* = 6.90 Hz, NCH_2_), 3.68 (t, 2H, *J* = 6.90 Hz, NCH_2_), 2.71–2.31 (m, 20H, 2 NCH_2_ and 8 NCH_2_), 2.30 (s, 6H, 2NCH_3_), 2.00–1.89 (m, 4H, 2CH_2_).

***2,4-bis{4-[(4-(4-methylpiperazin-1-yl)butyl)iminomethyl]phenyl}-6-phenylquinoline* (11d)**

Yellow oil (98%); ^1^H NMR (CDCl_3_) *δ* ppm: 8.35 (s, 1H, CH=N), 8.31 (s, 1H, CH=N), 8.26 (d, 1H, *J* = 8.70 Hz, H-8), 8.23 (d, 2H, *J* = 8.40 Hz, H-3″, and H-5″), 8.02 (d, 1H, *J* = 1.80 Hz, H-5), 7.97 (dd, 1H, *J* = 8.70, and 1.80 Hz, H-7), 7.87 (d, 2H, *J* = 8.40 Hz, H-3′and H-5′), 7.83 (d, 2H, *J* = 8.40 Hz, H-2″, and H-6″), 7.82 (s, 1H, H-3), 7.62 (d, 2H, *J* = 8.40 Hz, H-2′, and H-6′), 7.57 (d, 2H, *J* = 7.30 Hz, H-2, and H-6_phe_), 7.40 (t, 2H, *J* = 7.30 Hz, H-3, and H-5_phe_), 7.31 (t, 1H, *J* = 7.30 Hz, H-4_phe_), 3.65 (t, 2H, *J* = 6.90 Hz, NCH_2_), 3.61 (t, 2H, *J* = 6.90 Hz, NCH_2_), 2.68–2.30 (m, 20H, 2 NCH_2,_ and 8 NCH_2_), 2.32 (s, 6H, 2NCH_3_), 1.80–1.71 (m, 4H, 2CH_2_), 1.67–1.60 (m, 4H, 2CH_2_).

***2,4-bis{4-[(3-dimethylaminopropyl)iminomethyl]phenyl}-8-phenylquinoline* (11e)**

Yellow oil (88%); ^1^H NMR (CDCl_3_) *δ* ppm: 8.44 (s, 1H, CH=N), 8.34 (s, 1H, CH=N), 8.24 (d, 2H, *J* = 8.10 Hz, H-3″, and H-5″), 7.94 (d, 2H, *J* = 8.10 Hz, H-3′, and H-5′), 7.92 (s, 1H, H-3), 7.87 (dd, 1H, *J* = 8.40, and 1.50 Hz, H-7), 7.85 (dd, 1H, *J* = 8.40, and 1.50 Hz, H-5), 7.84–7.81 (m, 4H, H-2″, H-6″, H-2′, and H-6′), 7.65 (d, 2H, *J* = 8.10 Hz, H-2, and H-6_phe_), 7.58–7.52 (m, 3H, H-3_phe_, H-5_phe_, and H-6), 7.49 (t, 1H, *J* = 8.10 Hz, H-4_phe_), 3.74 (t, 2H, *J* = 6.90 Hz, NCH_2_), 3.68 (t, 2H, *J* = 6.90 Hz, NCH_2_), 2.42 (t, 2H, *J* = 6.90 Hz, NCH_2_), 2.38 (t, 2H, *J* = 6.90 Hz, NCH_2_), 2.29 (s, 6H, N(CH_3_)_2_), 2.23 (s, 6H, N(CH_3_)_2_), 1.98–1.85 (m, 4H, 2CH_2_).

***General procedure for 2,4-bis[(substituted-aminomethyl)phenyl]-phenylquinazolines 12a–n and 2,4-bis[(substituted-aminomethyl)phenyl]-phenylquinolines* 13a–e
**

To a solution of compounds **10**,**11** (0.4 mmol) in methanol (10 mL), we added portion-wise at 0 °C sodium borohydride (3.2 mmol, 8 eq.). The reaction mix was then stirred at room temperature for 1 h and subsequently heated under reflux for 1 h. It was then evaporated to dryness under reduced pressure. After cooling, the residue was triturated in water and extracted with dichloromethane (40 mL). The organic layer was separated, dried over sodium sulfate and activated charcoal, and evaporated to dryness. Oils were used without further purification to generate compounds **12**,**13**.

***2,4-bis{4-[(3-dimethylaminopropyl)aminomethyl]phenyl}-5-phenylquinazoline* (12a)**

Yellow oil (75%); ^1^H NMR (CDCl_3_) *δ* ppm: 8.72 (d, 2H, *J* = 8.10 Hz, H-3″, and H-5″), 8.16 (dd, 1H, *J* = 8.40, and 1.20 Hz, H-8), 7.91 (dd, 1H, *J* = 8.40, and 7.20 Hz, H-7), 7.57 (dd, 1H, *J* = 7.20, and 1.50 Hz, H-6), 7.48 (d, 2H, *J* = 8.10 Hz, H-3′, and H-5′), 7.31 (d, 2H, *J* = 8.10 Hz, H-2″, and H-6″), 7.09–6.95 (m, 7H, H-2′, H-6′, and H-phe), 3.89 (s, 2H, NCH_2_Ar), 3.66 (s, 2H, NCH_2_Ar), 2.68 (t, 2H, *J* = 7.20 Hz, NCH_2_), 2.58 (t, 2H, *J* = 7.20 Hz, NCH_2_), 2.37–2.33 (m, 4H, 2NCH_2_), 2.22 (s, 6H, N(CH_3_)_2_), 2.20 (s, 6H, N(CH_3_)_2_), 1.76–1.63 (m, 4H, 2CH_2_); ^13^C NMR (CDCl_3_) *δ* ppm: 169.3 (C-2), 160.3 (C-4), 155.0 (C-8a), 144.5 (C-1_phe_), 142.4 (C-5, C-4′, and C-4″), 140.0 (C-1″), 137.9 (C-1′), 134.1 (C-7), 131.8 (C-4_phe_), 131.6 (C-3 and C-5_phe_), 131.1 (C-3″ and C-5″), 130.1 (C-3′ and C-5′), 130.0 (C-8), 129.6 (C-2 and C-6_phe_), 129.0 (C-2″ and C-6″), 128.5 (C-2′ and C-6′), 127.9 (C-6), 121.3 (C-4a), 59.5 (NCH_2_), 55.2 (NCH_2_), 54.9 (NCH_2_), 49.3 (NCH_2_), 48.8 (NCH_2_), 47.0 (N(CH_3_)_2_), 29.5 (CH_2_); MALDI-TOF MS *m*/*z* [M + H]^+^ Calcd for C_38_H_47_N_6_: 587.3862, Found: 587.3845.

***2,4-bis{4-[(3-dimethylaminopropyl)aminomethyl]phenyl}-6-phenylquinazoline* (12b)**

Yellow oil (96%); ^1^H NMR (CDCl_3_) *δ* ppm: 8.66 (d, 2H, *J* = 8.10 Hz, H-3″, and H-5″), 8.29 (d, 1H, *J* = 1.80 Hz, H-5), 8.19 (d, 1H, *J* = 8.85 Hz, H-8), 8.11 (dd, 1H, *J* = 8.85, and 1.80 Hz, H-7), 7.90 (d, 2H, *J* = 8.10 Hz, H-3′, and H-5′), 7.62 (d, 2H, *J* = 8.10 Hz, H-2″, and H-6″), 7.54 (d, 2H, *J* = 8.10 Hz, H-2′, and H-6′), 7.49–7.35 (m, 5H, H_phe_), 3.93 (s, 2H, NCH_2_Ar), 3.88 (s, 2H, NCH_2_Ar), 2.75 (t, 2H, *J* = 7.20 Hz, NCH_2_), 2.70 (t, 2H, *J* = 7.20 Hz, NCH_2_), 2.36 (t, 2H, *J* = 7.20 Hz, NCH_2_), 2.33 (t, 2H, *J* = 7.20 Hz, NCH_2_), 2.24 (s, 6H, N(CH_3_)_2_), 2.22 (s, 6H, N(CH_3_)_2_), 1.79–1.64 (m, 4H, 2CH_2_); ^13^C NMR (CDCl_3_) *δ* ppm: 169.5 (C-2), 161.4 (C-4), 152.8 (C-8a), 144.1 (C-1″), 144.0 (C-6), 141.3 (C-1_phe_), 141.0 (C-1′), 138.4 (C-4′), 137.7 (C-4″), 134.5 (C-4_phe_), 131.7 (C-3″ and C-5″), 130.9 (C-8), 130.4 (C-3′ and C-5′), 130.1 (C-2′ and C-6′), 129.7 (C-2″, C-6″, C-3, C-5_phe_, and C-4_phe_), 129.3 (C-7), 128.7 (C-2 and C-6_phe_), 125.9 (C-5), 123.2 (C-4a), 59.5 (NCH_2_), 55.1 (NCH_2_), 49.5 (NCH_2_), 49.2 (NCH_2_), 46.9 (N(CH_3_)_2_), 29.3 (CH_2_); MALDI-TOF MS *m*/*z* [M+3H]^+^ Calcd for C_38_H_49_N_6_: 589.4018, Found: 589.551.

***2,4-bis{4-[(4-dimethylaminobutyl)aminomethyl]phenyl}-6-phenylquinazoline* (12c)**

Yellow oil (97%); ^1^H NMR (CDCl_3_) *δ* ppm: 8.66 (d, 2H, *J* = 8.10 Hz, H-3″, and H-5″), 8.30 (d, 1H, *J* = 1.20 Hz, H-5), 8.20 (d, 1H, *J* = 8.70 Hz, H-8), 8.13 (dd, 1H, *J* = 8.70, and 1.20 Hz, H-7), 7.91 (d, 2H, *J* = 8.10 Hz, H-3′, H-5′), 7.65 (d, 2H, *J* = 8.10 Hz, H-2″, and H-6″), 7.57 (d, 2H, *J* = 8.10 Hz, H-2′, and H-6′), 7.50–7.44 (m, 4H, H-2, H-3, H-5, and H-6_phe_), 7.40–7.36 (m, 1H, H-4_phe_), 3.94 (s, 2H, NCH_2_Ar), 3.89 (s, 2H, NCH_2_Ar), 2.78–2.66 (m, 4H, 2NCH_2_), 2.30–2.25 (m, 4H, 2NCH_2_), 2.21 (s, 6H, N(CH_3_)_2_), 2.21 (s, 6H, N(CH_3_)_2_), 1.83–1.74 (m, 4H, 2CH_2_), 1.59–1.47 (m, 4H, 2CH_2_); ^13^C NMR (CDCl_3_) *δ* ppm: 169.5 (C-8), 161.4 (C-4), 152.8 (C-8a), 144.2 (C-1″), 143.9 (C-6), 141.3 (C-1_phe_), 141.1 (C-1′), 138.4 (C-4′), 137.7 (C-4″), 134.5 (C-4_phe_), 131.7 (C-3″ and C-5″), 130.9 (C-8), 130.4 (C-3′ and C-5′), 130.1 (C-2′ and C-6′), 129.7 (C-2″, C-6″, C-3, C-5_phe_, and C-4_phe_), 129.3 (C-7), 128.7 (C-2 and C-6_phe_), 125.9 (C-5), 123.2 (C-4a), 65.9 (NCH_2_), 61.0 (NCH_2_), 55.1 (NCH_2_), 50.9 (NCH_2_), 50.6 (NCH_2_), 46.8 (N(CH_3_)_2_), 29.3 (CH_2_), 29.1 (CH_2_), 26.9 (CH_2_); MALDI-TOF MS *m*/*z* [M + H]^+^ Calcd for C_40_H_51_N_6_: 615.4175, Found: 615.4152.

***2,4-bis{4-[(4-(3-methylpiperazin-1-yl)propyl)aminomethyl]phenyl}-6-phenylquinazoline* (12d)**

Yellow oil (97%); ^1^H NMR (CDCl_3_) *δ* ppm: 8.59 (d, 2H, *J* = 8.25 Hz, H-3″ and H-5″), 8.21 (d, 1H, *J* = 1.90 Hz, H-5), 8.09 (d, 1H, *J* = 8.85 Hz, H-8), 8.01 (dd, 1H, *J* = 8.85, and 1.90 Hz, H-7), 7.82 (d, 2H, *J* = 8.25 Hz, H-3′and H-5′), 7.52 (d, 2H, *J* = 8.25 Hz, H-2″, and H-6″), 7.48 (d, 2H, *J* = 8.25 Hz, H-2′, and H-6′), 7.39 (d, 2H, *J* = 8.10 Hz, H-2, and H-6_phe_), 7.31 (t, 2H, *J* = 8.10 Hz H-3, and H-5_phe_), 7.28 (t, 1H, *J* = 8.10 Hz H-4_phe_), 3.83 (s, 2H, NCH_2_Ar), 3.79 (s, 2H, NCH_2_Ar), 2.67 (t, 2H, *J* = 6.90 Hz, NCH_2_), 2.62 (t, 2H, *J* = 6.90 Hz, NCH_2_), 2.50–2.25 (m, 20H, 2NCH_2, and_ 8NCH_2pip_), 2.19 (s, 3H, *J* = 7.20 Hz, NCH_3_), 2.17 (s, 3H, NCH_3_), 1.73–1.61 (m, 4H, 2CH_2_); ^13^C NMR (CDCl_3_) *δ* ppm: 169.4 (C-2), 161.3 (C-4), 152.7 (C-8a), 144.1 (C-1″), 144.0 (C-6), 141.2 (C-1_phe_), 140.9 (C-1′), 138.2 (C-4′), 137.6 (C-4″), 134.4 (C-4 phe), 131.7 (C-3″ and C-5″), 130.7 (C-8), 130.3 (C-3′ and C-5′), 130.0 (C-2′ and C-6′), 129.5 (C-2″, C-6″, C-3, C-5_phe_, and C-4_phe_), 129.3 (C-7), 128.6 (C-2 and C-6_phe_), 123.1 (C-4a), 58.4 (NCH_2_), 56.5 (NCH_2pip_), 55.10 (NCH_2_), 54.6 (NCH_2pip_), 49.7 (NCH_2_), 49.5 (NCH_2_), 47.4 (NCH_3_), 28.3 (CH_2_); MALDI-TOF MS *m*/*z* [M+2H]^+^ Calcd for C_44_H_58_N_8_: 698.4784, Found: 698.167.

***2,4-bis{4-[(4-(4-methylpiperazin-1-yl)butyl)aminomethyl]phenyl}-6-phenylquinazoline* (12e)**

Yellow oil (97%); ^1^H NMR (CDCl_3_) *δ* ppm: 8.64 (d, 2H, *J* = 8.25 Hz, H-3″, and H-5″), 8.31 (d, 1H, *J* = 1.80 Hz, H-5), 8.20 (d, 1H, *J* = 8.70 Hz, H-8), 8.13 (dd, 1H, *J* = 8.70, and 1.80 Hz, H-7), 7.90 (d, 2H, *J* = 8.25 Hz, H-3′and H-5′), 7.64 (d, 2H, *J* = 8.25 Hz, H-2″, and H-6″), 7.56 (d, 2H, *J* = 8.25 Hz, H-2′, and H-6′), 7.49–7.44 (m,4H-2, H-3, H-5, and H-6 _phe_), 7.41–7.36 (m, 1H, H-4 _phe_), 3.92 (s, 2H, NCH_2_Ar), 3.88 (s, 2H, NCH_2_Ar), 2.72–2.58 (m, 4H, 2NCH_2_), 2.50–2.29 (m, 20H, 2NCH_2, and_ 8NCH_2pip_), 2.27 (s, 6H, 2NCH_3_), 2.58–1.53 (m, 8H, 4CH_2_); ^13^C NMR (CDCl_3_) *δ* ppm: 169.5 (C-2), 161.4 (C-4), 152.8 (C-8a), 144.1 (C-1″), 144.0 (C-6), 141.3 (C-1_phe_), 141.1 (C-1′), 138.3 (C-4′), 137.7 (C-4″), 134.5 (C-4 phe), 131.7 (C-3″ and C-5″), 130.8 (C-8), 130.4 (C-3′ and C-5′), 130.1 (C-2′ and C-6′), 129.7 (C-2″ and C-6″), 129.6 (C-3, C-5_phe_, and C-4_phe_), 129.2 (C-7), 128.7 (C-2 and C-6_phe_), 123.1 (C-4a), 59.9 (NCH_2_), 56.5 (NCH_2pip_), 55.20 (NCH_2_), 54.5 (NCH_2pip_), 50.9 (NCH_2_), 50.6 (NCH_2_), 47.4 (NCH_3_), 29.5 (CH_2_), 26.1 (CH_2_); MALDI-TOF MS *m*/*z* [M + H]^+^ Calcd for C_46_H_61_N_8_: 725.5019, Found: 725.4992.

***2,4-bis{4-[(3-dimethylaminopropyl)aminomethyl]phenyl}-7-phenylquinazoline* (12f)**

Pale yellow oil (98%); ^1^H NMR (CDCl_3_) *δ* ppm: 8.63 (d, 2H, *J* = 8.10 Hz, H-3″, and H-5″), 8.29 (d, 1H, *J* = 1.50 Hz, H-8), 8.12 (d, 1H, *J* = 8.70 Hz, H-5), 7.83 (d, 2H, *J* = 8.10 Hz, H-3′, and H-5′), 7.73 (d, 2H, *J* = 8.10 Hz, H-2″, and H-6″), 7.71 (dd, 1H, *J* = 8.70, and 1.50 Hz, H-6), 7.52 (d, 2H, *J* = 8.10 Hz, H-2′, and H-6′), 7.49–7.37 (m, 5H, H_phe_), 3.89 (s, 2H, NCH_2_Ar), 3.84 (s, 2H, NCH_2_Ar), 2.71 (t, 2H, *J* = 7.05 Hz, NCH_2_), 2.66 (t, 2H, *J* = 7.05 Hz, NCH_2_), 2.31 (t, 2H, *J* = 7.05 Hz, NCH_2_), 2.29 (t, 2H, *J* = 7.05 Hz, NCH_2_), 2.21 (s, 6H, N(CH_3_)_2_), 2.18 (s, 6H, N(CH_3_)_2_), 1.75–1.61 (m, 4H, 2CH_2_); ^13^C NMR (CDCl_3_) *δ* ppm: 169.1 (C-2), 161.9 (C-4), 153.8 (C-8a), 147.3 (C-1″), 144.5 (C-7), 144.1 (C-1_phe_), 140.8 (C-1′), 138.3 (C-4′), 137.6 (C-4″), 131.6 (C-3″ and C-5″), 130.4 (C-3′ and C-5′), 129.9 (C-2′and C-6′), 129.5 (C-2″, C-6″,C-3, C-5, C-4_phe_), 128.8 (C-2, C-6_phe_, and C-8), 127.8 (C-6 and C-5), 122.0 (C-4a), 59.5 (NCH_2_), 55.2 (NCH_2_), 49.4 (NCH_2_), 49.3 (NCH_2_), 46.9 (N(CH_3_)_2_), 29.4 (CH_2_); MALDI-TOF MS *m*/*z* [M+2H]^+^ Calcd for C_38_H_48_N_6_: 588.394, Found: 588.988.

***2,4-bis{4-[(4-dimethylaminobutyl)aminomethyl]phenyl}-7-phenylquinazoline* (12g)**

Pale yellow oil (96%); ^1^H NMR (CDCl_3_) *δ* ppm: 8.65 (d, 2H, *J* = 8.10 Hz, H-3″, and H-5″), 8.33 (d, 1H, *J* = 1.50 Hz, H-8), 8.17 (d, 1H, *J* = 8.70 Hz, H-5), 7.88 (d, 2H, *J* = 8.10 Hz, H-3′and H-5′), 7.80–7.76 (m, 3H, H-2″, H-6″, and H-6), 7.56 (d, 2H, *J* = 8.10 Hz, H-2′, and H-6′), 7.52–7.40 (m, 5H, H_phe_), 3.93 (s, 2H, NCH_2_), 3.88 (s, 2H, NCH_2_), 2.70 (t, 2H, J=6.90 Hz, NCH_2_), 2.67 (t, 2H, J=6.90 Hz, NCH_2_), 2.31 (t, 2H, J = 6.90 Hz, NCH_2_), 2.29 (t, 2H, J=6.90 Hz, NCH_2_), 2.22 (s, 6H, N(CH_3_)_2_), 2.20 (s, 6H, N(CH_3_)_2_), 1.58–1.52 (m, 8H, 4CH_2_); ^13^C NMR (CDCl_3_) *δ* ppm: 169.2 (C-2), 161.9 (C-4), 153.8 (C-8a), 147.4 (C-1″), 144.1 (C-7), 143.9 (C-1_phe_), 140.9 (C-1′), 138.5 (C-4′), 137.7 (C-4″), 131.7 (C-3″ and C-5″), 130.4 (C-3′ and C-5′), 130.1 (C-2′ and C-6′), 129.9 (C-4_phe_), 129.7 (C-2″, C-6″, and C-8), 127.8 (C-6, C-5), 122.0 (C-4a), 61.0 (NCH_2_), 55.0 (NCH_2_), 50.6 (NCH_2_), 50.2 (NCH_2_), 46.7 (N(CH_3_)_2_), 29.3 (CH_2_), 26.9 (CH_2_); MALDI-TOF MS *m*/*z* [M + H]^+^ Calcd for C_40_H_51_N_6_: 615.4175, Found: 615.4171.

***2,4-bis{4-[(5-dimethylaminopentyl)aminomethyl]phenyl}-7-phenylquinazoline* (12h)**

Yellow oil (93%); ^1^H NMR (CDCl_3_) *δ* ppm: 8.65 (d, 2H, *J* = 8.10 Hz, H-3″, and H-5″), 8.32 (d, 1H, *J* = 1.50 Hz, H-8), 8.16 (d, 1H, *J* = 8.70 Hz, H-5), 7.86 (d, 2H, *J* = 8.10 Hz, H-3′, and H-5′), 7.78–7.52 (m, 3H, H-2″, H-6″, and H-6), 7.54 (d, 2H, *J* = 8.10 Hz, H-2′, and H-6′), 7.50–7.41 (m, 5H, H_phe_), 3.91 (s, 2H, NCH_2_), 3.86 (s, 2H, NCH_2_), 2.69 (t, 2H, *J* = 6.90 Hz, NCH_2_), 2.66 (t, 2H, *J* = 6.90 Hz, NCH_2_), 2.26 (t, 2H, *J* = 6.90 Hz, NCH_2_), 2.23 (t, 2H, *J* = 6.90 Hz, NCH_2_), 2.20 (s, 6H, N(CH_3_)_2_), 2.19 (s, 6H, N(CH_3_)_2_), 1.60–1.44 (m, 8H, 4CH_2_), 1.39–1.30 (m, 4H, 2CH_2_); ^13^C NMR (CDCl_3_) *δ* ppm: 169.2 (C-2), 161.9 (C-4), 153.8 (C-8a), 147.4 (C-1″), 144.4 (C-7), 144.0 (C-1_phe_), 140.9 (C-1′), 138.3 (C-4′), 137.7 (C-4″), 131.6 (C-3″ and C-5″), 130.4 (C-3′, C-5′and C-4_phe_), 130.1 (C-2′ and C-6′), 129.9 (C-8), 129.6 (C-2″, C-6″, C-3, and C-5_phe_), 128.9 (C-2 and C-6_phe_), 127.8 (C-6 and C-5), 122.0 (C-4a), 61.1 (NCH_2_), 55.2 (NCH_2_), 50.8 (NCH_2_), 50.7 (NCH_2_), 46.8 (N(CH_3_)_2_), 31.4 (CH_2_), 28.9 (CH_2_), 26.6 (CH_2_); MALDI-TOF MS *m*/*z* [M + H]^+^ Calcd for C_42_H_55_N_6_: 643.4488, Found: 643.4495.

***2,4-bis{4-[(4-(3-methylpiperazin-1-yl)propyl)aminomethyl]phenyl}-7-phenylquinazoline* (12i)**

Yellow oil (97%); ^1^H NMR (CDCl_3_) *δ* ppm: 8.66 (d, 2H, *J* = 8.10 Hz, H-3″, and H-5″), 8.35 (d, 1H, *J* = 1.50 Hz, H-8), 8.18 (d, 1H, *J* = 8.70 Hz, H-5), 7.89 (d, 2H, *J* = 8.10, H-3′, and H-5′), 7.79 (d, 2H, *J* = 8.10 Hz, H-2″and H-6″), 7.78 (dd, 1H, *J* = 8.70, and 1.50 Hz, H-6); 7.56 (d, 2H, *J* = 8.10 Hz, H-2′, and H-6′), 7.52–7.42 (m, 5, H_phe_), 3.93 (s, 2H, NCH_2_Ar), 3.88 (s, 2H, NCH_2_Ar), 2.76 (t, 2H, *J* = 6.90 Hz, NCH_2_), 2.70 (t, 2H, *J* = 6.90 Hz, NCH_2_), 2.58–2.35 (m, 20H, 2NCH_2, and_ 8NCH_2pip_), 2.28 (s, 3H, NCH_3_), 2.27 (s, 3H, NCH_3_),1.82–1.70 (m, 4H, 2CH_2_); ^13^C NMR (CDCl_3_) *δ* ppm: 169.2 (C-2), 162.0 (C-4), 153.8 (C-8a), 147.4 (C-1″), 144.0 (C-7), 144.1 (C-1_phe_), 140.9 (C-1′), 138.4 (C-4′), 137.7 (C-4″), 131.7 (C-3″ and C-5″), 130.5 (C-3′ and C-5′), 130.1 (C-2′ and C-6′), 129.9 (C-8), 129.6 (C-2″, C-6″, C-3, C-5_phe_, and C-4_phe_), 128.9 (C-2 and C-6_phe_), 127.9 (C-5 and C-6), 122.0 (C-4a), 58.4 (NCH_2_), 56.6 (NCH_2pip_), 55.2 (NCH_2_), 54.7 (NCH_2pip_), 49.6 (NCH_2_), 49.5 (NCH_2_), 47.4 (NCH_3_), 28.4 (CH_2_); MALDI-TOF MS *m*/*z* [M + H]^+^ Calcd for C_44_H_57_N_8_: 697.4706, Found: 697.516.

***2,4-bis{4-[(4-(4-methylpiperazin-1-yl)butyl)aminomethyl]phenyl}-7-phenylquinazoline* (12j)**

Yellow oil (96%); ^1^H NMR (CDCl_3_) *δ* ppm: 8.67 (d, 2H, *J* = 8.20 Hz, H-3″, and H-5″), 8.37 (d, 1H, *J* = 1.80 Hz, H-8), 8.20 (d, 1H, *J* = 8.70 Hz, H-5), 7.90 (d, 2H, *J* = 8.20 Hz, H-3′, and H-5′), 7.81 (dd, 1H, *J* = 8.70, and 1.80 Hz, H-6), 7.80 (d, 2H, *J* = 8.20 Hz, H-2″, and H-6″), 7.59 (d, 2H, *J* = 8.20 Hz, H-2′, and H-6′), 7.53 (d, 2H, *J* = 7.80 Hz, H-2, and H-6_phe_), 7.51–7.45 (m, 3H-2, H-3, H-4, and H-5 _phe_), 3.96 (s, 2H, NCH_2_), 3.91 (s, 2H, NCH_2_), 2.76–2.67 (m, 4H, 2NCH_2_), 2.53–2.32 (m, 20H, 2NCH_2, and_ 8NCH_2pip_), 2.29 (s, 3H, NCH_3_), 2.28 (s, 3H, NCH_3_), 1.62–1.55 (m, 8H, 4CH_2_); ^13^C NMR (CDCl_3_) *δ* ppm: 169.1 (C-2), 161.9 (C-4), 153.8 (C-8a), 147.4 (C-1″), 143.7 (C-7), 143.5 (C-1_phe_), 140.9 (C-1′), 138.3 (C-4′), 137.7 (C-4″), 131.7 (C-3″ and C-5″), 130.5 (C-3′,C-5′, and C-4_phe_), 130.2 (C-2′ and C-6′), 129.8 (C-2″, C-6″,C-3, and C-5_phe_), 128.9 (C-2, C-6_phe_, and C-8), 127.9 (C-5 and C-6), 122.0 (C-4a), 59.8 (NCH_2_), 56.4 (NCH_2pip_), 54.80 (NCH_2_), 54.5 (NCH_2pip_), 50.6 (NCH_2_), 50.5 (NCH_2_), 47.4 (NCH_3_), 29.5 (CH_2_), 26.1 (CH_2_); MALDI-TOF MS *m*/*z* [M + H]^+^ Calcd for C_46_H_61_N_8_: 725.5019, Found: 725.5022.

***2,4-bis{4-[(3-dimethylaminopropyl)aminomethyl]phenyl}-8-phenylquinazoline* (12k)**

Pale yellow oil (86%); ^1^H NMR (CDCl_3_) *δ* ppm: 8.53 (d, 2H, *J* = 8.10 Hz, H-3″, and H-5″), 8.08 (dd, 1H, *J* = 8.40, and 1.50 Hz, H-7), 7.91 (dd, 1H, *J* = 8.40, and 1.50 Hz, H-5), 7.86–7.83 (m, 4H, H-3′,H-5′, H-2″, and H-6″), 7.57–7.51 (m, 5H, H-2′,H-6′, H-6, H-2_phe_, and H-6_phe_), 7.47–7.42 (m, 1H, H-4_phe_), 7.39–7.35 (m, 2H, H-3, and H-5_phe_), 3.91 (s, 2H, NCH_2_), 3.82 (s, 2H, NCH_2_), 2.74 (t, 2H, *J* = 6.90 Hz, NCH_2_), 2.65 (t, 2H, *J* = 7.20 Hz, NCH_2_), 2.37 (t, 2H, *J* = 6.90 Hz, NCH_2_), 2.32 (t, 2H, *J* = 6.90 Hz, NCH_2_), 2.25 (s, 6H, N(CH_3_)_2_), 2.21 (s, 6H, N(CH_3_)_2_), 1.80–1.64 (m, 4H, 2CH_2_); ^13^C NMR (CDCl_3_) *δ* ppm: 167.5 (C-2), 158.3 (C-4), 148.5 (C-8a), 141.7 (C-1″), 141.4 (C-8), 139.3 (C-1_phe_), 137.5 (C-1′), 136.2 (C-4′), 135.6 (C-4″), 132.8 (C-4_phe_), 130.0 (C-3″ and C-5″, C-3′ and C-5′), 129.4 (C-3 and C-5_phe_), 127.8 (C-2″ and C-6″), 127.3 (C-2′and C-6′), 126.9 (C-2, C-8, and C-6_phe_), 126.5 (C-7), 125.5 (C-5), 125.4 (C-6), 121.1 (C-4a) 57.1 (NCH_2_), 52.8 (NCH_2_), 52.4 (NCH_2_), 47.0 (NCH_2_), 46.8 (NCH_2_), 44.6 (N(CH_3_)_2_), 44.5 (N(CH_3_)_2_), 27.0 (CH_2_), 26.9 (CH_2_); MALDI-TOF MS *m*/*z* [M + H]^+^ Calcd for C_38_H_47_N_6_: 587.3862, Found: 587.3851.

***2,4-bis{4-[(4-dimethylaminobutyl)aminomethyl]phenyl}-8-phenylquinazoline* (12l)**

Pale yellow oil (91%); ^1^H NMR (CDCl_3_) *δ* ppm: 8.52 (d, 2H, *J* = 8.10 Hz, H-3″, and H-5″), 8.06 (dd, 1H, *J* = 8.10, and 1.50 Hz, H-7), 7.88 (dd, 1H, *J* = 8.10, and 1.50 Hz, H-5), 7.84–7.81 (m, 4H, H-3′, H-5′, H-2″, and H-6″), 7.54–7.50 (m, 5H, H-2′,H-6′, H-2, H-6, and H-6_phe_), 7.45–7.42 (m, 1H, H-4_phe_), 7.39–7.36 (m, 2H, H-3, and H-5_phe_), 3.90 (s, 2H, NCH_2_), 3.81 (s, 2H, NCH_2_), 2.69 (t, 2H, *J* = 5.70 Hz, NCH_2_), 2.62 (t, 2H, *J* = 5.70 Hz, NCH_2_), 2.26 (t, 2H, *J* = 5.70 Hz, NCH_2_), 2.24 (t, 2H, *J* = 5.70 Hz, NCH_2_), 2.19 (s, 6H, N(CH_3_)_2_), 2.15 (s, 6H, N(CH_3_)_2_), 1.54–1.47 (m, 8H, 4CH_2_); ^13^C NMR (CDCl_3_) *δ* ppm: 167.5 (C-2), 158.3 (C-4), 148.4 (C-8a), 141.5 (C-1″), 141.3 (C-8), 139.2 (C-1_phe_), 137.4 (C-1′), 136.2 (C-4′), 135.6 (C-4″), 132.8 (C-4_phe_), 130.0 (C-3″ and C-5″), 129.3 (C-3′ and C-5′), 127.8 (C-3 and C-5_phe_), 127.2 (C-2″, C-6″, C-2′, and C-6′), 126.8 (C-2 and C-6_phe_), 126.5 (C-7), 125.5 (C-5), 125.4 (C-6), 121.1 (C-4a), 58.6 (NCH_2_), 58.5 (NCH_2_), 52.7 (NCH_2_), 52.6 (NCH_2_), 48.3 (NCH_2_), 48.1 (NCH_2_), 44.4 (N(CH_3_)_2_), 44.3 (N(CH_3_)_2_), 26.9 (CH_2_), 26.8 (CH_2_), 24.5 (CH_2_), 24.4 (CH_2_); MALDI-TOF MS *m*/*z* [M + H]^+^ Calcd for C_40_H_51_N_6_: 615.4175, Found: 615.4169.

***2,4-bis{4-[(4-(3-methylpiperazin-1-yl)propyl)aminomethyl]phenyl}-8-phenylquinazoline* (12m)**

Pale yellow oil (71%); ^1^H NMR (CDCl_3_) *δ* ppm: 8.51 (d, 2H, *J* = 8.10 Hz, H-3″, and H-5″), 8.06 (dd, 1H, *J* = 8.30, and 1.50 Hz, H-7), 7.88 (dd, 1H, *J* = 8.30, and 1.50 Hz, H-5), 7.84–7.81 (m, 4H, H-3′, H-5′, H-2″, and H-6″), 7.55–7.48 (m, 5H, H-2′, H-6′, H-2_phe_, H-6_phe_, and H-6), 7.44–7.42 (m, 1H, H-4 phe), 7.37–7.34 (m, 2H, H-3, and H-5_phe_), 3.88 (s, 2H, NCH_2_), 3.79 (s, 2H, NCH_2_), 2.70 (t, 2H, *J* = 5.10 Hz, NCH_2_), 2.63 (t, 2H, *J* = 5.10 Hz, NCH_2_), 2.53–2.29 (m, 20H, 2NCH_2, and_ 8NCH_2pip_), 1.72 (qt, 2H, *J* = 5.10 Hz, CH_2_), 1.66 (qt, 2H, *J* = 5.10 Hz, 2CH_2_); ^13^C NMR (CDCl_3_) *δ* ppm: 167.5 (C-2), 158.3 (C-4), 148.4 (C-8a), 141.7 (C-1″), 141.4 (C-8), 139.3 (C-1_phe_), 137.4 (C-1′), 136.1 (C-4′), 135.6 (C-4″), 132.8 (C-4_phe_), 130.0 (C-3″, C-5″,C-3′, and C-5′), 129.3 (C-3 and C-5_phe_), 127.8 (C-2″ and C-6″), 127.1 (C-2′ and C-6′), 126.8 (C-2 and C-6_phe_), 126.5 (C-7), 125.5 (C-5), 125.4 (C-6), 121.1 (C-4a), 56.0 (NCH_2_), 55.9 (NCH_2_), 54.2 (NCH_2pip_), 54.1 (NCH_2pip_), 52.7 (NCH_2_), 52.3 (NCH_2pip_), 52.2 (NCH_2pip_), 47.2 (NCH_2_), 47.0 (NCH_2_), 45.1 (NCH_3_), 45.0 (NCH_3_), 26.0 (CH_2_), 25.8 (CH_2_); MALDI-TOF MS *m*/*z* [M + H]^+^ Calcd for C_44_H_57_N_8_: 697.4706, Found: 697.4691.

***2,4-bis{4-[(4-(4-methylpiperazin-1-yl)butyl)aminomethyl]phenyl}-8-phenylquinazoline* (12n)**

Pale yellow oil (93%); ^1^H NMR (CDCl_3_) *δ* ppm: 8.53 (d, 2H, *J* = 8.10 Hz, H-3″, and H-5″), 8.09 (dd, 1H, *J* = 8.40, and 1.20 Hz, H-7), 7.91 (dd, 1H, *J* = 8.40, and 1.20 Hz, H-5), 7.88–7.83 (m, 4H, H-3′, H-5′, H-2″, and H-6″), 7.58–7.50 (m, 5H, H-2′, H-6′, H-2_phe_, H-6_phe_, and H-6), 7.48–7.44 (m, 1H, H-4_phe_), 7.42–7.40 (m, 2H, H-3, and H-5_phe_), 3.92 (s, 2H, NCH_2_), 3.83 (s, 2H, NCH_2_), 2.71 (t, 2H, *J* = 5.70 Hz, NCH_2_), 2.63 (t, 2H, *J* = 5.70 Hz, NCH_2_), 2.54–2.30 (m, 20H, 2NCH_2, and_ 8NCH_2pip_), 1.59–1.50 (m, 8H, 4CH_2_); ^13^C NMR (CDCl_3_) *δ* ppm: 168.5 (C-2), 159.3 (C-4), 149.4 (C-8a), 143.0 (C-1″), 142.5 (C-8), 140.2 (C-1_phe_), 138.4 (C-1′), 137.1 (C-4′), 136.6 (C-4″), 133.8 (C-4_phe_) 131.0 (C-3″ and C-5″), 130.3 (C-3′ and C-5′), 128.8 (C-3 and C-5_phe_), 128.2 (C-2″ and C-6″), 128.1 (C-2′ and C-6′), 127.8 (C-2, C-6_phe_), 127.5 (C-7), 126.5 (C-5), 126.4 (C-6), 122.1 (C-4a), 58.6 (NCH_2_), 58.5 (NCH_2_), 55.1 (NCH_2pip_), 53.8 (NCH_2_), 53.3 (NCH_2pip_), 53.2 (NCH_2pip_), 49.4 (NCH_2_), 49.2 (NCH_2_), 46.1 (NCH_2_), 28.2 (CH_2_), 28.1 (CH_2_), 24.8 (CH_2_), 24.7 (CH_2_); MALDI-TOF MS *m*/*z* [M + H]^+^ Calcd for C_46_H_61_N_8_: 725.5019, Found: 725.5015.

***2,4-bis{4-[(3-dimethylaminopropyl)aminomethyl]phenyl}-6-phenylquinoline* (13a)**

Yellow oil (97%); ^1^H NMR (CDCl_3_) *δ* ppm: 8.31 (d, 1H, *J* = 8.70 Hz, H-8), 8.18 (d, 2H, *J* = 8.10 Hz, H-3″, and H-5″), 8.11 (d, 1H, *J* = 1.65 Hz, H-5), 7.99 (dd, 1H, *J* = 8.70, and 1.65 Hz, H-7), 7.83 (s, 1H, H-3), 7.62 (d, 2H, *J* = 8.10 Hz, H-3′and H-5′), 7.57 (d, 2H, *J* = 8.10 Hz, H-2″, and H-6″), 7.52 (d, 2H, *J* = 8.10 Hz, H-2′, and H-6′), 7.48 (d, 2H, *J* = 7.20 Hz, H-2, and H-6_phe_), 7.45 (t, 2H, *J* = 7.20 Hz, H-3, and H-5_phe_), 7.36 (t, 1H, *J* = 7.20 Hz, H-4 _phe_), 3.92 (s, 2H, NCH_2_), 3.89 (s, 2H, NCH_2_), 2.77 (t, 2H, *J* = 7.20 Hz, NCH_2_), 2.71 (t, 2H, *J* = 7.20 Hz, NCH_2_), 2.38 (t, 2H, *J* = 7.20 Hz, NCH_2_), 2.34 (t, 2H, *J* = 7.20 Hz, NCH_2_), 2.25 (s, 6H, N(CH_3_)_2_), 2.24 (s, 6H, N(CH_3_)_2_), 1.81–1.66 (m, 4H, 2CH_2_); ^13^C NMR (CDCl_3_) *δ* ppm: 158.0 (C-2), 150.5 (C-4), 149.6 (C-8a), 143.2 (C-1″), 142.3 (C-6), 142.0 (C-1_phe_), 140.3 (C-1′), 139.6 (C-4′), 138.3 (C-4″), 131.9 (C-4_phe_), 131.0 (C-3″ and C-5″), 130.6 (C-8), 130.2 (C-3′ and C-5′), 129.9 (C-3 and C-5_phe_), 129.8 (C-2′ and C-6′), 128.9 (C-2″, C-6″, and C-7), 128.8 (C-2 and C-6_phe_), 127.3 (C-4a), 124.8 (C-5), 121.1 (C-3), 59.5 (NCH_2_), 55.2 (NCH_2_), 49.2 (NCH_2_), 47.0 (N(CH_3_)_2_), 29.4 (CH_2_); MALDI-TOF MS *m*/*z* [M + H]^+^ Calcd for C_39_H_48_N_5_: 586.3910, Found: 586.4230.

***2,4-bis{4-[(4-dimethylaminobutyl)aminomethyl]phenyl}-6-phenylquinoline* (13b)**

Yellow oil (yield, 97%); ^1^H NMR (CDCl_3_) *δ* ppm: 8.29 (d, 1H, *J* = 8.70 Hz, H-8), 8.17 (d, 2H, *J* = 8.10 Hz, H-3″, and H-5″), 8.11 (d, 1H, *J* = 1.80 Hz, H-5), 7.99 (dd, 1H, *J* = 8.70, and 1.80 Hz, H-7), 7.83 (s,1H,H-3), 7.62 (d, 2H, *J* = 8.10 Hz, H-3′, and H-5′), 7.58 (d, 2H, *J* = 8.10 Hz, H-2″, and H-6″), 7.52 (d, 2H, *J* = 8.10 Hz, H-2′, and H-6′), 7.47 (d, 2H, *J* = 7.20 Hz, H-2, and H-6_phe_), 7.45 (t, 2H, *J* = 7.20 Hz, H-3, and H-5_phe_), 7.35 (t, 1H, *J* = 7.20 Hz, H-4_phe_), 3.92 (s, 2H, NCH_2_), 3.88 (s, 2H, NCH_2_), 2.74 (t, 2H, *J* = 6.90 Hz, NCH_2_), 2.68 (t, 2H, *J* = 6.90 Hz, NCH_2_), 2.30 (t, 2H, *J* = 6.90 Hz, NCH_2_), 2.28 (t, 2H, *J* = 6.90 Hz, NCH_2_), 2.23 (s, 6H, N(CH_3_)_2_), 2.22 (s, 6H, N(CH_3_)_2_), 1.60–1.52 (m, 4H, 2CH_2_); ^13^C NMR (CDCl_3_) *δ* ppm: 158.0 (C-2), 150.5 (C-4), 149.6 (C-8a), 143.1 (C-1″), 142.2 (C-6), 142.0 (C-1_phe_), 140.3 (C-1′), 139.7 (C-4′), 138.3 (C-4″), 131.8 (C-4_phe_), 131.0 (C-3″ and C-5″), 130.6 (C-8), 130.2 (C-3′ and C-5′), 130.0 (C-3 and C-5_phe_), 129.8 (C-2′ and C-6′), 129.0 (C-2″, C-6″, and C-7) 128.8 (C-2 and C-6_phe_), 127.3 (C-4a), 124.8 (C-5), 121.1 (C-3), 61.0 (NCH_2_), 55.1 (NCH_2_), 50.9 (NCH_2_), 50.6 (NCH_2_), 46.8 (N(CH_3_)_2_), 29.3 (CH_2_), 26.8 (CH_2_); MALDI-TOF MS *m*/*z* [M + H]^+^ Calcd for C_41_H_52_N_5_: 614.4223, Found: 614.4225.

***2,4-bis{4-[(4-(3-methylpiperazin-1-yl)propyl)aminomethyl]phenyl}-6-phenylquinoline* (13c)**

Yellow oil (65%); ^1^H NMR (CDCl_3_) *δ* ppm: 8.29 (d, 1H, *J* = 9.00 Hz, H-8), 8.18 (d, 2H, *J* = 8.10 Hz, H-3″, and H-5″), 8.11 (d, 1H, *J* = 1.80, H-5), 7.99 (dd, 1H, *J* = 9.00, and 1.80 Hz, H-7), 7.83 (s,1H, H-3), 7.62 (d, 2H, *J* = 8.10 Hz, H-3′and H-5′), 7.57 (d, 2H, *J* = 8.10 Hz, H-2″, and H-6″), 7.51 (d, 2H, *J* = 8.10 Hz, H-2′, and H-6′), 7.47 (d, 2H, *J* = 7.50 Hz, H-2, and H-6_phe_), 7.44 (t, 2H, *J* = 7.50 Hz, H-3, and H-5_phe_), 7.36 (t, 1H, *J* = 7.50 Hz, H-4_phe_), 3.92 (s, 2H, NCH_2_), 3.88 (s, 2H, NCH_2_), 2.78 (t, 2H, *J* = 6.90 Hz, NCH_2_), 2.73 (t, 2H, *J* = 6.90 Hz, NCH_2_), 2.65–2.50 (m, 20H, 2NCH_2, and_ 8NCH_2pip_), 2.28 (s, 3H, NCH_3_), 2.26 (s, 3H, NCH_3_), 1.83–1.69 (m, 4H, 2CH_2_); ^13^C NMR (CDCl_3_) *δ* ppm: 158.0 (C-2), 150.5 (C-4), 149.6 (C-8a), 143.2 (C-1″), 142.3 (C-6), 142.0 (C-1_phe_), 140.3 (C-1′), 139.7 (C-4′), 138.3 (C-4″), 131.9 (C-4_phe_) 131.0 (C-3″ and C-5″), 130.6 (C-8), 130.3 (C-3′ and C-5′), 129.9 (C-3 and C-5_phe_), 129.7 (C-2′ and C-6′), 128.9 (C-7, C-2″ and C-6″), 128.8 (C-2 and C-6_phe_), 127.3 (C-4a), 124.8 (C-5), 121.0 (C-3), 58.4 (NCH_2_), 56.6 (NCH_2pip_), 55.1 (NCH_2_), 54.7 (NCH_2pip_), 47.4 (NCH_3_), 28.4 (CH_2_); MALDI-TOF MS *m*/*z* [M + H]^+^ Calcd for C_47_H_62_N_7_: 696.4753, Found: 696.4740.

***2,4-bis{4-[(4-(4-methylpiperazin-1-yl)butyl)aminomethyl]phenyl}-6-phenylquinoline* (13d)**

Yellow oil (76%); ^1^H NMR (CDCl_3_) *δ* ppm: 8.28 (d, 1H, *J* = 8.70 Hz, H-8), 8.17 (d, 2H, *J* = 8.10 Hz, H-3″, and H-5″), 8.10 (d, 1H, *J* = 1.80, H-5), 7.99 (dd, 1H, *J* = 8.70, and 1.80 Hz, H-7),7.82 (s,1H, H-3), 7.62 (d, 2H, *J* = 8.10 Hz, H-3′and H-5′), 7.57 (d, 2H, *J* = 8.10 Hz, H-2″, and H-6″), 7.50 (d, 2H, *J* = 8.10 Hz, H-2′, and H-6′), 7.45 (d, 2H, *J* = 7.50 Hz, H-2, and H-6_phe_), 7.41 (t, 2H, *J* = 7.50 Hz, H-3, and H-5_phe_), 7.33 (t, 1H, *J* = 7.50 Hz, H-4_phe_), 3.92 (s, 2H, NCH_2_), 3.88 (s, 2H, NCH_2_), 2.74 (t, 2H, *J* = 6.60 Hz, NCH_2_), 2.67 (t, 2H, *J* = 6.60 Hz, NCH_2_), 2.50–2.45 (m, 20H, 2NCH_2, and_ 8NCH_2pip_), 2.28 (s, 6H, 2NCH_3_), 1.59–1.53 (m, 8H, 4CH_2_); ^13^C NMR (CDCl_3_) *δ* ppm: 158.0 (C-2), 150.5 (C-4), 149.6 (C-8a), 143.2 (C-1″), 142.3 (C-6), 142.0 (C-1_phe_), 140.3 (C-1′), 139.7 (C-4′), 138.4 (C-4″), 131.9 (C-4_phe_) 131.0 (C-3″ and C-5″), 130.6 (C-8), 130.0 (C-3′ and C-5′), 129.8 (C-2′ and C-6′), 128.9 (C-2″ and C-6″), 128.8 (C-7, C-2_phe_, and C-6_phe_), 127.3 (C-4a), 124.8 (C-5), 121.0 (C-3), 59.9 (NCH_2_), 56.5 (NCH_2pip_), 55.1 (NCH_2_), 54.6 (NCH_2pip_), 47.4 (NCH_3_), 29.5 (CH_2_), 26.2 (CH_2_); MALDI-TOF MS *m*/*z* [M + H]^+^ Calcd for C_47_H_62_N_7_: 724.5067, Found: 724.5074.

***2,4-bis{4-[(3-dimethylaminopropyl)aminomethyl]phenyl}-8-phenylquinoline* (13e)**

Yellow oil (83%); ^1^H NMR (CDCl_3_) *δ* ppm: 8.16 (d, 2H, *J* = 8.10 Hz, H-3″, and H-5″), 7.92 (dd, 1H, *J* = 8.40, and 1.50 Hz, H-7), 7.90 (d, 2H, *J* = 8.10 Hz, H-3′, and H-5′), 7.88 (s, 1H, H-3), 7.79 (dd, 1H, *J* = 8.40, and 1.50 Hz, H-5), 7.58–7.35 (m, 10H, H-2″, H-6″, H-2′, H-6′, H-6, and H_phe_), 3.94 (s, 2H, NCH_2_), 3.85 (s, 2H, NCH_2_), 2.79 (t, 2H, *J* = 6.90 Hz, NCH_2_), 2.69 (t, 2H, *J* = 6,90 Hz, NCH_2_), 2.37 (t, 2H, *J* = 6.90 Hz, NCH_2_), 2.33 (t, 2H, *J* = 6.90 Hz, NCH_2_), 2.27 (s, 6H, N(CH_3_)_2_), 2.26 (s, 6H, N(CH_3_)_2_), 1.80–1.68 (m, 4H, 2CH_2_); ^13^C NMR (CDCl_3_) *δ* ppm: 156.6 (C-2), 150.6 (C-4), 147.5 (C-8a), 143.1 (C-1″), 142.4 (C-8), 142.1 (C-1_phe_), 141.3 (C-1′), 139.5 (C-4′), 138.8 (C-4″), 132.6 (C-3″ and C-5″), 131.7 (C-4_phe_), 131.1 (C-3′ and C-5′), 129.9 (C-3 and C-5_phe_), 129.7 (C-2″ and C-6″), 129.0 (C-2′and C-6′), 128.9 (C-2 and C-6_phe_), 128.4 (C-7), 127.7 (C-4a), 127.3 (C-5), 126.7 (C-6), 119.8 (C-3), 59.4 (NCH_2_), 55.1 (NCH_2_), 49.4 (NCH_2_), 49.2 (NCH_2_), 46.9 (N(CH_3_)_2_), 29.4 (CH_2_); MALDI-TOF MS *m*/*z* [M + H]^+^ Calcd for C_39_H_48_N_5_: 586.3910, Found: 586.5060.

***2-(4-Formylphenyl)-5-phenyl-3H-quinazolin-4-one* (14)**

White crystals (10%); m.p. = 252 °C. ^1^H NMR (CDCl_3_) *δ* ppm: 11.99 (s, 1H, NH), 10.13 (s, 1H, CHO), 8.16 (d, 2H, *J* = 8.40 Hz, H-3′, and H-5′), 7.85 (dd, 1H, *J* = 7.50, and 1.40 Hz, H-6), 7.82 (d, 2H, *J* = 8.40 Hz, H-2′and H-6′), 7.79 (dd, 1H, *J* = 8.40, and 7.50 Hz, H-7), 7.47–7.29 (m, 6H, H-8, and H-phe); ^13^C NMR (CDCl_3_) *δ* ppm: 191.7 (CHO), 163.6 (CON), 150.6 (C-2), 143.5 (C-8a), 141.6 (C-4′), 137.7 (C-1_phe_), 137.4 (C-1′), 133.8 (C-7), 131.0 (C-5), 130.1 (C-3′ and C-5′), 129.0 (C-3_phe_ and C-5_phe_), 128.1 (C-4_phe_), 127.9 (C-2_phe_ and C-6_phe_), 127.5 (C-2′ and C-6′), 127.2 (C-6), 117.6 (C-8); MS-ES^+^ *m*/*z* [M + H]^+^ Calcd for C_21_H_15_N_2_O_2_: 327.1134, Found: 327.1130. 

### 3.2. FRET-Melting Experiments

FRET-melting experiments were conducted on a Stratagene MX3005P (Thermo Fisher Scientific, Illkirch-Graffenstaden, France) real-time PCR equipment in 96-well plates on the DNA sequences reported in Table 4. Experiments were performed in 10 mM lithium cacodylate buffer (pH 7.2) and either 10 mM KCl and 90 mM LiCl (FK-RAST, FBCL-2T, F21T, and FdxT) or 1 mM KCl and 99 mM LiCl (Fc-MYCT) concentrations, depending on the T_m_ of the G4s alone. The DNA concentration was 0.2 µM. The stabilization induced by the compounds was calculated as the difference (ΔTm) between the average transition temperature of the nucleic acid alone and that measured with the appropriate ligand concentration (2 µM). Data are presented as an average of three independent measurements, each conducted in duplicate conditions (λ_exc_ = 492 nm, λ_em_ = 516 nm, T interval = 25–95 °C, ramp: 25 °C for 5 min, then 1 °C/min, measurements every 1 °C, 8× magnification of the fluorescence signal). 

### 3.3. Native MS

The affinity and stoichiometry of binding to oligonucleotides were determined by native electrospray ionization mass spectrometry in the negative ion mode on a Thermo Orbitrap Exactive mass spectrometer calibrated daily, and operated in negative mode, on a 400–3000 *m*/*z* scan range, using the 50,000 resolution setting, with the following tuning parameters: spray voltage: 3.2 kV, capillary temperature: 170 °C, sheath gas: 60, aux gas: 0, heater temperature: 35 °C, tube lens voltage: −175 V, capillary voltage: −17 V, skimmer voltage: −22 V. The syringe injection flow rate was 3 µL/min. These parameters ensured a good signal intensity without disrupting noncovalent complexes, which was verified using d[G_4_T_4_G_4_]_2_ as a control [37]. Oligonucleotides were purchased lyophilized, and RP-cartridge purified from Eurogentec (Kanaka Eurogentec, Seraing, Belgium), then buffer exchanged with ammonium acetate (5 M; Sigma-Aldrich, Saint-Quentin-Fallavier, France) using Amicon Ultra (3 K cut-off; Merck Millipore, Cork, Ireland). Sample solutions were prepared by diluting the proper volume of oligonucleotide stock solutions to reach 10 µM of DNA and 20 µM of ligand, in 100 mM ammonium acetate. The solutions were incubated for 16 h at 4 °C before analysis. *K*d were calculated as already described [29]. 

### 3.4. Circular Dichroism

CD experiments were performed with a JASCO J-1500 spectropolarimeter (JASCO, Lisses, France) using quartz cells of 2 mm path length. The scans were recorded at 22 °C, from 220 to 450 nm with the following parameters: 1.0 nm data pitch, 2 nm bandwidth, 0.5 s response, 50 nm/min scanning speed; they are the result of three accumulations. Solutions were prepared as in Section 3.3. CD data were blank subtracted then normalized against molar dichroic absorption (Δ*ε*, in cm^−1^·M^−1^) using the equation below, where *θ* is the ellipticity in millidegrees, *C* is the oligonucleotide concentration in mol/l, and *l* is the path length in centimeters:

$$∆\varepsilon =\frac{\theta }{32,980\times C\times l}$$

### 3.5. Determination of Antiproliferative Effects (MTT Assay)

The growth-inhibitory effects of the compounds were determined by MTT (3-(4,5-dimethylthiazol-2-yl)-2,5-diphenyltetrazolium bromide) assay on selected cell lines, including HeLa and SiHa (cervical cancers), MDA-MB-231 and MCF-7 (breast cancers), and A2780 ovarian cancer cells [38]. All cell lines were purchased from the European Collection of Cell Cultures (Salisbury, UK) except SiHa, which was obtained from the American Tissue Culture Collection (Manassas, VA, USA). The cells were kept in minimum essential medium supplemented with 10% fetal bovine serum, 1% non-essential amino acids, and 1% penicillin-streptomycin-amphotericin-B at 37 °C in a humidified atmosphere containing 5% CO_2_. Media and supplements were obtained from Lonza Group Ltd., (Basel, Switzerland). Cancer cells were seeded onto 96-well plates (5000 cells/well), after an overnight incubation the test compounds were added in six concentrations (0.1, 0.3, 1.0, 3.0, 10.0, 30.0 µM) and incubated for 72 h under cell-culture condition. After the incubation, 20 μL of 5 mg/mL MTT solution was added to each well and incubated for a further 4 h. The medium was removed, and the precipitated formazan crystals were dissolved in DMSO for 30 min under shaking at 37 °C. The absorbance was measured at 545 nm with a microplate reader. Untreated cells were included as controls. Calculations were performed by means of the GraphPad Prism 5.01 software (GraphPad Software Inc., San Diego, CA, USA). At least two independent determinations were performed with 5 parallel wells for each condition. 

The human leukemic cell line K562 was grown in RPMI 1640 medium (Life Technology-Invitrogen by Thermo Fisher Scientific, Saint-Aubin, France) supplemented with 10% fetal calf serum (FCS), antibiotics (100 U/mL penicillin, 100 µg/mL streptomycin), and L-glutamine, (Eurobio, Les Ulis, France) at 37 °C, 5% CO_2_ in air. The MTS cell proliferation assay (Promega, Charbonnières-les-Bains, France) is a colorimetric assay system, which measures the transformation of a tetrazolium component (MTS) into formazan produced by the mitochondria of viable cells. Cells were washed twice in PBS (Phosphate Buffer Saline) and plated in quadruplicate into microtiter-plate wells in 100 μL culture media with or without our various compounds at increasing concentrations (0, 1, 5, 10, 20, and 50 μM) for 1, 2, and 3 days. After 3 h of incubation at 37 °C with 20 μL MTS/well, the plates were read using an ELISA microplate reader (Thermo, Electrocorporation, Waltham, MA, USA) at 490 nm. The amount of color produced was directly proportional to the number of viable cells. The results are expressed as the concentrations inhibiting cell growth by 50% after a 3-day incubation period. The 50% cytotoxic concentrations (CC_50_) were determined by linear regression analysis, expressed in μM ± SD (Microsoft Excel). 

The MTT proliferation test on the epithelial cell line HEK293 was performed as pre-viously described [39]. The 50% inhibiting concentrations (IC_50_) were determined by linear regression analysis. 

### 3.6. Telomerase Assay

Telomerase activity was assessed using the TRAP assay kit (TRAPeze^®^ RT telomerase detection kit, Chemicon, Millipore Sigma S7700, Merck KGaA, Darmstadt, Germany) according to manufacturer’s instructions with some modifications. Briefly, 106 were resuspended in CHAPS lysis buffer and proteins were extracted. Protein extracts and various concentrations of compounds (0 to 5 µM) were used to extend a synthetic telomeric DNA using an enzymatic reaction (30 °C during 20 min), followed by a quantification of telomeric DNA using a telomeric PCR amplification: 95 °C for 3 min, 2 cycles of 95 °C for 20 s, and 49 °C for 20 s, followed by 30 cycles 95 °C for 20 s and 60 °C for 20 s with signal acquisition) in a Stratagene Mx3005P system (Agilent, Les Ulis, France) using specific telomeric PCR quantitative primers [40]. Each sample was measured in technical duplicate with control DNA. For each drug and each dilution, we used biological triplicate of cell lysates. 

## 4. Conclusions

In this study, our team designed and synthesized a series of new 2,4-bis[(substituted-aminomethyl)phenyl]phenylquinazoline and 2,4-bis[(substituted-aminomethyl)phenyl]phenylquinoline derivatives **12,13** and their cytotoxic activities against five human cancer cell lines (HeLa, SiHa, MCF-7, MDA-MB-231, A2780) were investigated. Their cytotoxicity on the human leukemic K562 cell line was also evaluated. Their ability to stabilize various oncogene promoter G4-structures (c-MYC, K-RAS, and BCL-2) was also determined through a FRET-melting assay. Native mass spectrometry experiments showed that **12b**, **12e**, or **13a** can bind to the human telomeric sequence and the oncogene promoters BCL-2 in the micromolar range. Significant conformational changes allowed the binding of a second ligand; again, in the micromolar range. Conversely, binding to the parallel G-quadruplex formed by the c-MYC promoters was mostly 1:1 and with no conformational change. By using these biological and biophysical data, preliminary SAR studies on these quinazoline and quinoline compounds were discussed. The pharmacological evaluations of these new nitrogen compounds **12**,**13** showed antiproliferative activities against the different human and gynecologic cancer cell lines, and also the K562 leukemic cell line. The disubstituted 6- and 7-phenyl-bis(3-dimethylaminopropyl)aminomethylphenyl-quinazolines **12b**, **12f**, and **12i** displayed the most interesting antiproliferative activities against these human cancer cell lines, as well as 6-phenyl-bis(3-dimethylaminopropyl)aminomethylphenylquinoline **13a**. Based on our presented results, these novel substituted phenylquinazolines and phenylquinolines can be regarded as attractive cores for the design and synthesis of innovative molecules with potent anticancer actions. The most effective members of the current library are suitable for further mechanistic studies. 

## Figures and Tables

**Table 1:** Antiproliferative activities of compounds **12,13**.

Compound	IC_50_ Values (μM) ± SEM ^a^						
	HeLa	SiHa	MCF-7	MDA-MB-231	A2780	K562	HEK293
**12a**	1.36 ± 0.04	0.62 ± 0.12	1.12 ± 0.13	1.45 ± 0.14	2.60 ± 0.03	14.0 ± 0.2	n.d. ^b^
**12b**	0.82 ± 0.31	1.08 ± 0.10	0.58 ± 0.02	0.70 ± 0.16	1.20 ± 0.27	14.0 ± 0.3	>30
**12c**	2.21 ± 0.86	0.76 ± 0.01	1.44 ± 0.03	1.07 ± 0.42	2.04 ± 0.54	7.0 ± 0.2	n.d. ^b^
**12d**	1.32 ± 0.02	1.28 ± 0.27	1.01 ± 0.30	1.44 ± 0.17	1.74 ± 0.11	17.0 ± 0.4	n.d. ^b^
**12e**	1.27 ± 0.02	0.63 ± 0.05	1.27 ± 0.01	0.89 ± 0.46	2.19 ± 0.41	13.0 ± 0.2	n.d. ^b^
**12f**	1.51 ± 0.09	0.75 ± 0.10	0.58 ± 0.08	0.55 ± 0.01	0.79 ± 0.19	14.0 ± 0.3	>30
**12g**	0.95 ± 0.51	0.52 ± 0.22	0.63 ± 0.35	0.67 ± 0.35	1.74 ± 0.89	12.0 ± 0.3	3.98 ± 0.12
**12h**	2.31 ± 0.76	0.75 ± 0.01	1.19 ± 0.27	1.10 ± 0.38	1.43 ± 0.07	13.0 ± 0.2	n.d. ^b^
**12i**	1.15 ± 0.36	0.92 ± 0.24	0.67 ± 0.31	0.52 ± 0.02	1.14 ± 0.16	23.0 ± 0.5	>30
**12j**	1.48 ± 0.80	0.33 ± 0.08	0.58 ± 0.25	1.10 ± 0.15	1.44 ± 0.10	8.0 ± 0.2	4.90 ± 0.15
**12k**	1.19 ± 0.24	1.01 ± 0.27	1.24 ± 0.14	1.36 ± 0.06	1.34 ± 0.14	7.0 ± 0.1	n.d. ^b^
**12l**	1.39 ± 0.16	1.14 ± 0.51	1.45 ± 0.02	1.55 ± 0.12	2.08 ± 0.48	7.0 ± 0.2	n.d. ^b^
**12m**	1.17 ± 0.23	0.88 ± 0.14	1.22 ± 0.19	2.00 ± 0.45	2.10 ± 0.48	7.0 ± 0.2	n.d. ^b^
**12n**	3.51 ± 0.17	1.89 ± 0.74	4.17 ± 0.50	3.94 ± 0.54	7.10 ± 1.15	14.0 ± 0.3	n.d. ^b^
**13a**	0.50 ± 0.01	0.99 ± 0.15	0.54 ± 0.01	0.58 ± 0.02	0.90 ± 0.04	10.0 ± 0.4	>30
**13b**	3.58 ± 0.18	1.76 ± 0.61	3.03 ± 0.64	3.68 ± 0.80	6.49 ± 1.76	13.0 ± 0.4	n.d. ^b^
**13c**	1.30 ± 0.06	1.45 ± 0.13	0.86 ± 0.38	1.33 ± 0.13	1.58 ± 0.10	34.0 ± 0.7	n.d. ^b^
**13d**	2.27 ± 0.89	1.11 ± 0.37	1.62 ± 0.09	1.64 ± 0.11	2.10 ± 0.09	14.0 ± 0.3	n.d. ^b^
**13e**	1.28 ± 0.21	1.07 ± 0.45	1.12 ± 0.10	1.21 ± 0.03	1.50 ± 0.34	7.0 ± 0.1	n.d. ^b^

^a^ Standard error from two independent determinations duplicate. ^b^ n.d.: not done.

**Table 2:** Selectivity indexes of selected compounds **12,13**.

Compound	Selectivity Index ^a^					
	HEK293/HeLa	HEK293/SiHa	HEK293/MCF-7	HEK293/MDA-MB-231	HEK293/A2780	HEK293/K562
**12b**	>36.6	>27.8	>51.7	>42.8	>25.0	>2.14
**12f**	>19.9	>40.0	>51.7	>54.5	>38.0	>2.14
**12g**	4.19	7.65	6.32	5.94	2.29	0.33
**12i**	>26.1	>32.6	>44.8	>57.7	>26.3	>1.30
**12j**	3.31	14.85	8.45	4.45	3.40	0.61
**13a**	>60.0	>30.3	>55.5	>51.7	>33.3	>3.0

^a^ SI was defined as the ratio between the IC_50_ value on the HEK293 normal cells and the IC_50_ value against the leukemia HeLa, SiHa, MCF-7, MDA-MB-231, A2780 or K562 cells.

**Table 3:** FRET-melting values for compounds **12**,**13** and PhenDC3.

Compound	ΔT_1/2_ (°C) ^a^				
	Fc-MYCT	FK-RAST	FBCL-2T	F21T	FdxT
PhenDC3	11.5 ± 0.3	16.9 ± 0.4	13.1 ± 0.1	21.2 ± 0.5	−0.6 ± 0.1
**12a**	9.3 ± 0.3	10.6 ± 0.6	7.4 ± 0.9	6.8 ± 0.8	0.9 ± 0.2
**12b**	5.8 ± 0.9	17.4 ± 0.6	8.9 ± 0.7	23.7 ± 1.7	0.4 ± 0.3
**12c**	17.7 ± 0.8	24.5 ± 0.7	16.4 ± 0.4	28.0 ± 0.4	3.8 ± 0.8
**12d**	8.4 ± 0.9	15.5 ± 0.5	9.7 ± 0.06	15.8 ± 1.2	0.5 ± 0.3
**12e**	19.5 ± 1.0	33.1 ± 2.1	22.7 ± 0.4	33.9 ± 0.5	5.4 ± 1.6
**12f**	9.1 ± 1.4	20.1 ± 0.3	12.6 ± 0.6	16.3 ± 1.5	2.4 ± 0.1
**12g**	14.7 ± 0.8	24.3 ± 1.1	17.5 ± 0.6	19.2 ± 1.0	3.2 ± 0.3
**12h**	18.7 ± 0.2	28.6 ± 2.1	18.2 ± 1.7	28.1 ± 2.3	2.7 ± 1.0
**12i**	12.5 ± 0.7	20.2 ± 0.5	14.8 ± 0.7	18.6 ± 0.2	1.9 ± 0.1
**12j**	14.1 ± 0.3	18.7 ± 1.6	9.8 ± 1.0	14.5 ± 1.2	1.5 ± 0.4
**12k**	9.9 ± 1.4	13.0 ± 0.4	9.5 ± 0.2	17.7 ± 0.3	0.8 ± 0.1
**12l**	14.2 ± 1.1	18.7 ± 0.8	17.2 ± 1.2	24.9 ± 0.3	2.9 ± 0.2
**12m**	14.6 ± 0.9	18.2 ± 0.6	15.5 ± 0.3	23.3 ± 0.7	2.2 ± 0.2
**12n**	17.0 ± 2.0	18.0 ± 2.5	12.0 ± 2.0	20.7 ± 3.1	2.4 ± 0.7
**13a**	7.3 ± 1.0	15.0 ± 0.3	7.7 ± 0.6	21.5 ± 0.4	0.4 ± 0.1
**13b**	13.8 ± 3.0	19.6 ± 3.5	11.1 ± 2.6	13.6 ± 2.9	1.5 ± 0.6
**13c**	6.8 ± 1.4	14.5 ± 0.8	7.5 ± 0.5	11.3 ± 0.9	0.1 ± 0.2
**13d**	10.6 ± 0.7	12.7 ± 2.2	5.0 ± 1.1	8.0 ± 1.5	0.8 ± 0.2
**13e**	5.5 ± 0.9	11.6 ± 1.4	8.3 ± 0.1	15.9 ± 0.4	0.2 ± 0.1

^a^ All experiments were conducted in K^+^ conditions at 2 μM. ΔT_m_ of Fc-MYCT, FK-RAST, FBCL-2T, F21T, and FdxT (0.2 μM) were recorded in 10 mM lithium cacodylate (pH 7.2), 10 mM KCl, 90 mM LiCl. Compound PhenDC3 was tested at 0.5 μM. Error margins correspond to SD of three replicates.

**Table 4:** Labelled DNA sequences used in the present study.

Name	Sequences ^a^	Topology
FK-RAST	FAM-A GGG C GG TGT GGG AAGA GGG A-TAMRA	Parallel
FBCL-2T	FAM-GGG CGC GGG A GG AAG GGG GC GGG-TAMRA	Parallel
F21T	FAM-GGG TTA GGG TTA GGG TTA GGG-TAMRA	Hybrid
Fc-MYCT	FAM-GGG T GGG TA GGG T GGG TAA-TAMRA	Parallel
FdxT	FAM-TAT AGC TAT A-hexaethylene glycol-T ATA GCT ATA-TAMRA	Hairpin duplex

^a^ All sequences are provided in the 5′ => 3′direction.

**Table 5:** Binding affinities of **12b**, **12e**, **13a,** and **13d** for BCL-2, 24TTG, c-MYC, and ds26 determined by native mass spectrometry, expressed as percentage of bound DNA and dissociation constants (*K*d_1_ and *K*d_2_ correspond to two successive binding events). The second binding event is cooperative if *K*d_1_/*K*d_2_ > 4 [34].

	12b	12e	13a	13d
**BCL-2**				
*Bound DNA (%)*	*79*	*79*	*92*	*33*
*K*d1	5.6	6.2	1.6	53
*K*d2	2.8	2.2	0.75	20
*K*d1/*K*d2	2.0	2.8	2.1	2.6
**24TTG**				
*Bound DNA (%)*	*77*	*72*	*81*	*39*
*K*d1	9.5	14	3.7	29
*K*d2	1.5	1.9	7.1	83
*K*d1/*K*d2	6.3	7.2	0.5	0.4
**c-MYC**				
*Bound DNA (%)*	*70*	*75*	*78*	*44*
*K*d1	5.8	4.3	3.8	20
*K*d2	100	74	36	440
*K*d1/*K*d2	0.1	0.1	0.1	0.1
**ds26**				
*Bound DNA (%)*	*48*	*36*	*53*	*18*
*K*d1	17	30	14	85
*K*d2	130	280	95	460
*K*d1/*K*d2	0.1	0.1	0.2	0.2

## Data Availability

Data is contained within the article and supplementary material.

## Supplementary Materials

The following supporting information can be downloaded at: https://www.mdpi.com/article/10.3390/ph17010030/s1, Table S1: Physical properties of amines **12a**–**n** and **13a**–**e**; Figure S1: FRET-melting curves of compounds **12c**, **12g** and **13c**; Figure S2: Circular dichroism spectra of solutions containing **12b**, **12e**, **13a** or **13d** (20 μM) with 24TTG, BCL-2 or c-MYC (10 μM) in 100 mM ammonium acetate (pH = 6.8) (DNA + ligand; blue) compared to a reference with DNA alone (grey). The positions of characteristic bands at around 240, 260, and 290 nm are shown with dashed grey lines; Figure S3: Native electrospray mass spectra of solutions containing **12b**, **12e**, **13a** or **13d** (20 μM) with 24TTG (10 μM) in 100 mM ammonium acetate (pH = 6.8), zoomed on the 4- and 5- charge states: M = oligonucleotide monomer, L = ligand; Figure S4: Native electrospray mass spectra of solutions containing **12b**, **12e**, **13a** or **13d** (20 μM) with BCL-2 (10 μM) in 100 mM ammonium acetate (pH = 6.8), zoomed on the 4- and 5- charge states: M = oligonucleotide monomer, L = ligand; Figure S5: Native electrospray mass spectra of solutions containing **12b**, **12e**, **13a** or **13d** (20 μM) with c-MYC (10 μM) in 100 mM ammonium acetate (pH = 6.8), zoomed on the 4- and 5- charge states: M = oligonucleotide monomer, L = ligand; Figure S6: Native electrospray mass spectra of solutions containing **12b**, **12e**, **13a** or **13d** (20 μM) with ds26 (10 μM) in 100 mM ammonium acetate (pH = 6.8), zoomed on the 4-, 5- and 6- charge states: M = oligonucleotide monomer, L = ligand; CD spectra, native electrospray mass spectra, physical properties of amines **12a**–**n** and **13a**–**e**. ^1^H, ^13^C NMR and MS spectra for quinazolines **12** and quinolines **13** are also available (Figures S7–S63).

## References

[1] World Cancer Research Fund International. https://www.wcrf.org/cancer-trends/worldwide-cancer-data/.

[2] World Health Organization Cancer. https://www.who.int/news-room/fact-sheets/detail/cancer.

[3] Vasan N., Baselga J., Hyman D.M. (2019). A View on Drug Resistance in Cancer. Nature.

[4] Bajaj S., Kumar M.S., Peters G.J., Mayur Y.C. (2020). Targeting telomerase for its advent in cancer therapeutics. Med. Res. Rev..

[5] Dolinnaya N.G., Ogloblina A.M., Yakubovskaya M.G. (2016). Structure, properties, and biological relevance of the DNA and RNA G-quadruplexes: Overview 50 years after their discovery. Biochemistry.

[6] Roxo C., Kotkowiak W., Pasternak A. (2019). G-quadruplex forming aptamers—Characteristics, applications, and perspectives. Molecules.

[7] Ivancich M., Schrank Z., Wojdyla L., Leviskas B., Kuckovic A., Sanjali A., Puri N. (2017). Treating Cancer by Targeting Telomeres and Telomerase. Antioxidants.

[8] Mengual Gomez D.L., Armando R.G., Cerrudo C.S., Ghiringhelli P.D., Gomez D.E. (2016). Telomerase as a Cancer Target. Development of New Molecules. Curr. Med. Chem..

[9] Lipinska N., Romaniuk A., Paszel-Jaworska A., Toton E., Kopczynski P., Rubis B. (2017). Telomerase and drug resistance in cancer. Cell. Mol. Life Sci..

[10] Zidanloo S.G., Colagar A.H., Ayatollahi H., Bagheryan Z. (2019). G-quadruplex forming region within WT1 promoter is selectively targeted by daunorubicin and mitoxantrone: A possible mechanism for anti-leukemic effect of drugs. J. Biosci..

[11] Asamitsu S., Obata S., Yu Z., Bando T., Sugiyama H. (2019). Recent Progress of Targeted G-Quadruplex-Preferred Ligands Toward Cancer Therapy. Molecules.

[12] Sun Z.-Y., Wang X.-N., Cheng S.-Q., Su X.-X., Ou T.-M. (2019). Developing Novel G-Quadruplex Ligands: From Interaction with Nucleic Acids to Interfering with Nucleic Acid-Protein Interaction. Molecules.

[13] Che T., Wang Y.-Q., Huang Z.L., Tan J.-H., Huang Z.-S., Chen S.B. (2018). Natural Alkaloids and Heterocycles as G-Quadruplex Ligands and Potential Anticancer Agents. Molecules.

[14] Duarte A.R., Cadoni E., Ressurreição A.S., Moreira R., Paulo A. (2018). Design of Modular G-quadruplex Ligands. ChemMedChem.

[15] Ilyinsky N.S., Varizhuk A.M., Beniaminov A.D., Puzanov M.A., Shchyolkina A.K., Kaluzhny D.N. (2014). G-quadruplex ligands: Mechanisms of anticancer action and target binding. Mol. Biol..

[16] Liu J.N., Guo J.F., Zhou J.M., Feng G.K., Huang Z.S., Gu L.Q., Zeng Y.X., Zhu X.F. (2007). Inhibition of myc promoter and telomerase activity and induction of delayed apoptosis by SYUIQ-5, a novel G-quadruplex interactive agent in leukemia cells. Leukemia.

[17] Jiang Y., Chen A.-C., Kuang G.-T., Wang S.-K., Ou T.-M., Tan J.-H., Li D., Huang Z.-S. (2016). Design, synthesis and biological evaluation of 4-anilinoquinazoline derivatives as new c-myc G-quadruplex lignads. Eur. J. Med. Chem..

[18] Navneetha O., Deepthi K., Rao A.M., Jyostna T.S. (2017). A review on chemotherapeutic activities of quinoline. Int. J. Pharm. Chem. Biol. Sci..

[19] Man R.-J., Jeelani N., Zhou C., Yang Y.-S. (2021). Recent progress in the development of quinoline derivatives for the exploitation of anti-cancer agents. Anticancer Med. Chem..

[20] Hussaini S.M.A. (2016). Therapeutic significance of quinolines: A patent review (2013–2015). Expert Opin. Ther. Pat..

[21] Wdowiak P., Matysiak J., Kuszta P., Czarnek K., Niezabitowska E., Baj T. (2021). Quinazoline derivatives as potential therapeutic agents in urinary bladder cancer therapy. Front. Chem..

[22] Desplat V., Vincenzi M., Lucas R., Moreau S., Savrimoutou S., Pinaud N., Lesbordes J., Peyrilles E., Marchivie M., Routier S. (2016). Synthesis and evaluation of the cytotoxic activity of novel ethyl 4-[4-(4-substitutedpiperidin-1-yl)]benzyl-phenylpyrrolo[1,2-*a*]quinoxaline-carboxylate derivatives in myeloid and lymphoid leukemia cell lines. Eur. J. Med. Chem..

[23] Desplat V., Vincenzi M., Lucas R., Moreau S., Savrimoutou S., Rubio S., Pinaud N., Bigat D., Enriquez E., Marchivie M. (2017). Synthesis and Antiproliferative Effect of Ethyl 4-[4-(4-Substituted Piperidin-1-yl)]benzylpyrrolo[1,2-*a*]quinoxalinecarboxylate Derivatives on Human Leukemia Cells. ChemMedChem.

[24] Guillon J., Vincenzi M., Pinaud N., Ronga L., Rossi F., Savrimoutou S., Moreau S., Desplat V., Marchivie M. (2016). Synthesis and Crystal Structure of 3-{4-[(4-(2-Oxo-2,3-dihydro-1*H*-benzimidazol- 1-yl)piperidin-1-yl)benzyl]}-2-phenylindole. Struct. Chem. Crystallogr. Commun..

[25] Guillon J., Savrimoutou S., Rubio S., Desplat V. (2018). 1-Methyl-3-{4-[(4-(2-oxo-2,3-dihydro-1*H*-benzimidazol-1-yl)piperidin-1-yl)benzyl]}-2-phenylindole. Molbank.

[26] Das R.N., Chevret E., Desplat V., Rubio S., Mergny J.-L., Guillon J. (2018). Design, Synthesis and Biological Evaluation of New Substituted Diquinolinyl-Pyridine Ligands as Anticancer Agents by Targeting G-Quadruplex. Molecules.

[27] Guillon J., Savrimoutou S., Rubio S., Moreau S., Pinaud N., Marchivie M., Desplat V. (2020). 1-Phenyl-8-[[4-(pyrrolo[1,2-*a*]quinoxalin-4-yl)phenyl]methyl]-1,3,8-triazaspiro[4.5]decan-4-one: Synthesis, Crystal Structure and Anti-Leukemic Activity. Molbank.

[28] Gueddouda N.M., Hurtado M.R., Moreau S., Ronga L., Das R.N., Savrimoutou S., Rubio S., Marchand A., Mendoza O., Marchivie M. (2017). Design, Synthesis, and Evaluation of 2,9-Bis[(substituted-aminomethyl)phenyl]-1,10-phenanthroline Derivatives as G-Quadruplex Ligands. ChemMedChem.

[29] Guillon J., Denevault-Sabourin C., Chevret E., Brachet-Botineau M., Milano V., Guedin-Beaurepaire A., Moreau S., Ronga L., Savrimoutou S., Rubio S. (2021). Design, synthesis, and antiproliferative effect of 2,9-bis[4-(pyridinylalkylaminomethyl)phenyl]-1,10-phenanthroline derivatives on human leukemic cells by targeting G-quadruplex. Arch. Pharm. (Weinheim).

[30] CCDC-2238804 and CCDC-2238806 Contain the Supplementary Crystallographic Data for This Paper. These Data Can Be Obtained Free of Charge from the Cambridge Crystallographic Data Centre, University Chemical Lab, Lensfield Road, Cambridge, CB2 1EW, UK. https://www.ccdc.cam.ac.uk/.

[31] Liu X., Fu H., Jiang Y., Zhao Y. (2009). A Simple and Highly Efficient Approach to Quinazolinones under Mild Copper-Catalyzed Conditions. Angew. Chem. Int. Ed..

[32] Kappe T., Karem A.S., Stadlbauer W. (1988). Synthesis of benzo-halogenated 4-hydroxy-2(1*H*)-quinolones. J. Heterocycl. Chem..

[33] Largy E., König A., Ghosh A., Ghosh D., Benabou S., Rosu F., Gabelica V. (2022). Mass Spectrometry of Nucleic Acid Noncovalent Complexes. Chem. Rev..

[34] Rosu F., De Pauw E., Gabelica V. (2008). Electrospray mass spectrometry to study drug-nucleic acids interactions. Biochimie.

[35] Largy E., Marchand A., Amrane S., Gabelica V., Mergny J.-L. (2016). Quadruplex Turncoats: Cation-Dependent Folding and Stability of Quadruplex-DNA Double Switches. J. Am. Chem. Soc..

[36] Ghosh A., Trajkovski M., Teulade-Fichou M.-P., Gabelica V., Plavec J. (2022). Phen-DC3 Induces Refolding of Human Telomeric DNA into a Chair-Type Antiparallel G-Quadruplex through Ligand Intercalation. Angew. Chem..

[37] Balthasart F., Plavec J., Gabelica V. (2013). Ammonium Ion Binding to DNA G-Quadruplexes: Do Electrospray Mass Spectra Faithfully Reflect the Solution-Phase Species?. J. Am. Soc. Mass Spectrom..

[38] Mosmann T. (1983). Rapid Colorimetric Assay for Cellular Growth and Survival: Application to Proliferation and Cytotoxicity Assays. J. Immunol. Methods.

[39] Zhang G., Tang Z., Fan S., Li C., Li Y., Liu W., Long X., Zhang W., Zhang Y., Li Z. (2023). Synthesis and biological assessment of indole derivatives containing penta-heterocycles scaffold as novel anticancer agents towards A549 and K562 cells. J. Enzyme Inhib. Med. Chem..

[40] Cawthon R.M. (2009). Telomere length measurement by a novel monochrome multiplex quantitative PCR method. Nucleic Acids Res..

