# Supplementary material for: New 2,4-bis[(substituted-aminomethyl)phenyl]phenylquinazoline and 2,4-bis[(substituted-aminomethyl)phenyl]phenylquinoline Derivatives: Synthesis and Biological Evaluation as Novel Anticancer Agents by Targeting G-Quadruplex
Source: Pharmaceuticals (Basel). 2023 Dec 25;17(1):30. doi: 10.3390/ph17010030 (PMC10819771; doi:10.3390/ph17010030)
Supplement: Supplementary file 1 [file pharmaceuticals-17-00030-s001.zip › pharmaceuticals-2741246-supplementary.pdf]

Supplementary Materials

New 2,4-bis[(substituted-aminomethyl)phenyl]phenylquinazoline and 2,4-bis[(substituted-aminomethyl)phenyl]phenylquinoline Derivatives: Synthesis and Biological Evaluation as Novel Anticancer Agents by Targeting G-Quadruplex

Jean Guillon [1], Marc Le Borgne [2,*], Vittoria Milano [1], Aurore Guédin-Beaurepaire [1], Stéphane Moreau [1], Noël Pinaud [3], Luisa Ronga [4], Solène Savrimoutou [1], Sandra Albenque-Rubio [1], Mathieu Marchivie [5], Haouraa Kalout [1], Charley Walker [1], Louise Chevallier [1], Corinne Buré [6], Eric Largy [7], Valérie Gabelica [7], Jean-Louis Mergny [8], Virginie Baylot [9], Jacky Ferrer [10], Yamina Idrissi [10], Edith Chevret [10], David Cappellen [10,11], Vanessa Desplat [10], Zsuzsanna Schelz [12] and István Zupkó [12,*]

[1] INSERM, CNRS, ARNA, U1212, UMR 5320, UFR des Sciences Pharmaceutiques, Univ. Bordeaux, F-33076 Bordeaux, France; jean.guillon@u-bordeaux.fr (J.G.); vickymilano88@gmail.com (V.M.); aurore.guedin-beaurepaire@u-bordeaux.fr (A.G.-B.); stephane.moreau@u-bordeaux.fr (S.M.); solene.savrimoutou@u-bordeaux.fr (S.S.); sandra.rubio@u-bordeaux.fr (S.A.-R.); hkalout171996@gmail.com (H.K.); charley.walker@etu.u-bordeaux.fr (C.W.); chevallier.louise@yahoo.fr (L.C.)

[2] Small Molecules for Biological Targets Team, Centre de Recherche en Cancérologie de Lyon, Centre Léon Bérard, CNRS 5286, INSERM 1052, Université Claude Bernard Lyon 1, Univ. Lyon, F-69373 Lyon, France

[3] ISM—CNRS UMR 5255, Univ. Bordeaux, F-33405 Talence, France; noel.pinaud@u-bordeaux.fr (N.P.)

[4] E2S UPPA, CNRS, IPREM, Université de Pau et des Pays de l'Adour, F-64053 Pau, France; luisa.ronga@univ-pau.fr

[5] ICMCB—UMR 5026, Univ. Bordeaux, F-33608 Pessac, France; mathieu.marchivie@u-bordeaux.fr

[6] CNRS, INSERM, IECB, US1, UAR 3033, Univ. Bordeaux, F-33600 Pessac, France; corinne.bure@u-bordeaux.fr

[7] CNRS, INSERM, ARNA, UMR 5320, U1212, IECB, Univ. Bordeaux, F-33600 Pessac, France; eric.largy@u-bordeaux.fr (E.L.); valerie.gabelica@inserm.fr (V.G.)

[8] Ecole Polytechnique, Laboratoire d'Optique et Biosciences, CNRS, INSERM, Institut Polytechnique de Paris, F-91120 Palaiseau, France; jean-louis.mergny@inserm.fr

[9] Centre de Recherche en Cancérologie de Marseille (CRCM), Institut Paoli-Calmettes, CNRS UMR7258, Inserm U1068, Univ. Aix Marseille, F-13009 Marseille, France; virginie.baylot@inserm.fr

[10] INSERM UMR1312, BRIC, BoRdeaux Institute of onCology, Univ. Bordeaux, F-33076 Bordeaux, France; jacky_ferrer@yahoo.fr (J.F.); yamina.idrissi@u-bordeaux.fr (Y.I.); edith.chevret@u-bordeaux.fr (E.C.); david.cappellen@u-bordeaux.fr (D.C.); vanessa.desplat@u-bordeaux.fr (V.D.)

[11] Service Tumor Biology and Tumor Bank Laboratory, Groupe Hospitalier Bordeaux, CHU Bordeaux, F-33000 Bordeaux, France

[12] Institute of Pharmacodynamics and Biopharmacy, Faculty of Pharmacy, University of Szeged, 6720 Szeged, Hungary; schelz.zsuzsanna@szte.hu

* Correspondence: marc.le-borgne@univ-lyon1.fr (M.L.B.); zupko.istvan@szte.hu (I.Z.)

Table S1. Physical properties of amines **12a-n** and **13a-e**.

Compound	Crystal color	Salt[a]	mp (°C)[b]	Yield (%)[c]
**2a**	Yellow	4 $(COOH)_2$	147-149	67
**2b**	Yellow	4 $(COOH)_2$	234-236	71
**2c**	Yellow	4 $(COOH)_2$	152-155	63
**2d**	Pale-yellow	6 $(COOH)_2$	226-228	67
**2e**	White	6 $(COOH)_2$	165-167	50
**2f**	White	4 $(COOH)_2$	223-225	74
**2g**	Pale-yellow	4 $(COOH)_2$	145-147	66
**2h**	Pale-yellow	4 $(COOH)_2$	144-146	65
**2i**	Pale-yellow	6 $(COOH)_2$	221-223	60
**2j**	Pale-yellow	6 $(COOH)_2$	187-189	55
**2k**	White	4 $(COOH)_2$	211-213	81
**2l**	Pale-yellow	4 $(COOH)_2$	186-188	73
**2m**	White	6 $(COOH)_2$	224-226	52
**2n**	White	6 $(COOH)_2$	177-179	58
**3a**	Yellow	4 $(COOH)_2$	192-194	63
**3b**	Yellow	4 $(COOH)_2$	140-142	62
**3c**	Pale-yellow	6 $(COOH)_2$	219-221	84
**3d**	Yellow	6 $(COOH)_2$	165-167	51
**3e**	Pale-yellow	4 $(COOH)_2$	174-176	57

[a] The stoichiometry and composition of the salts were determined by elemental analyses and obtained values were within ± 0.4% of the theoretical values. [b] Crystallization solvent: 2-PrOH–H_2O. [c] The yields only included the conversions into the ammonium oxalates.

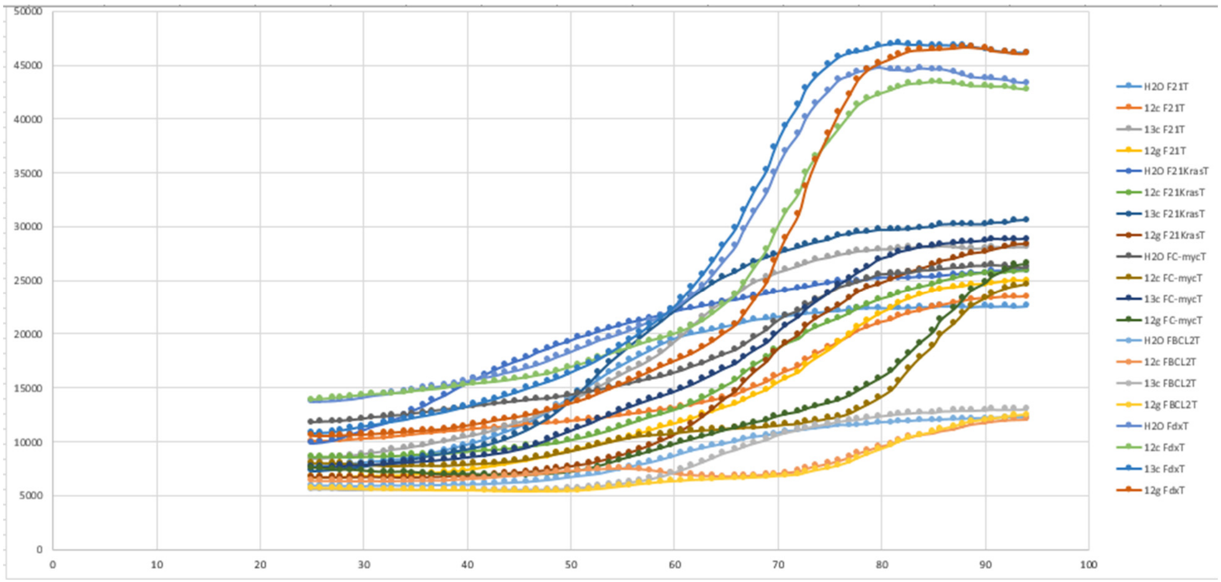

Figure S1. FRET-melting curves of compounds **12c**, **12g** and **13c**.

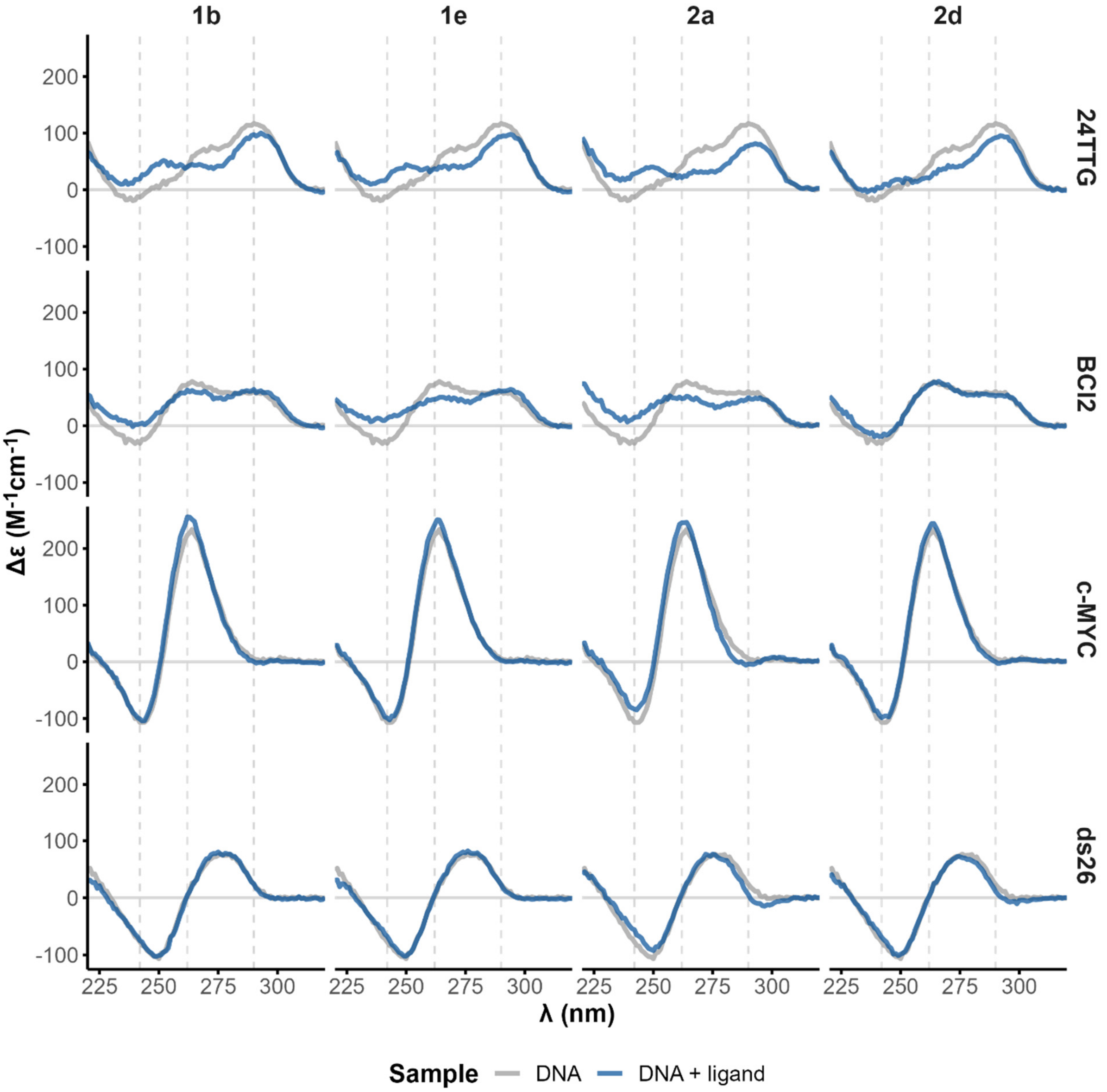

Figure S2. Circular dichroism spectra of solutions containing **12b**, **12e**, **13a** or **13d** (20 μM) with 24TTG, BCL-2 or c-MYC (10 μM) in 100 mM ammonium acetate (pH = 6.8) (DNA + ligand; blue) compared to a reference with DNA alone (grey). The positions of characteristic bands at around 240, 260, and 290 nm are shown with dashed grey lines.

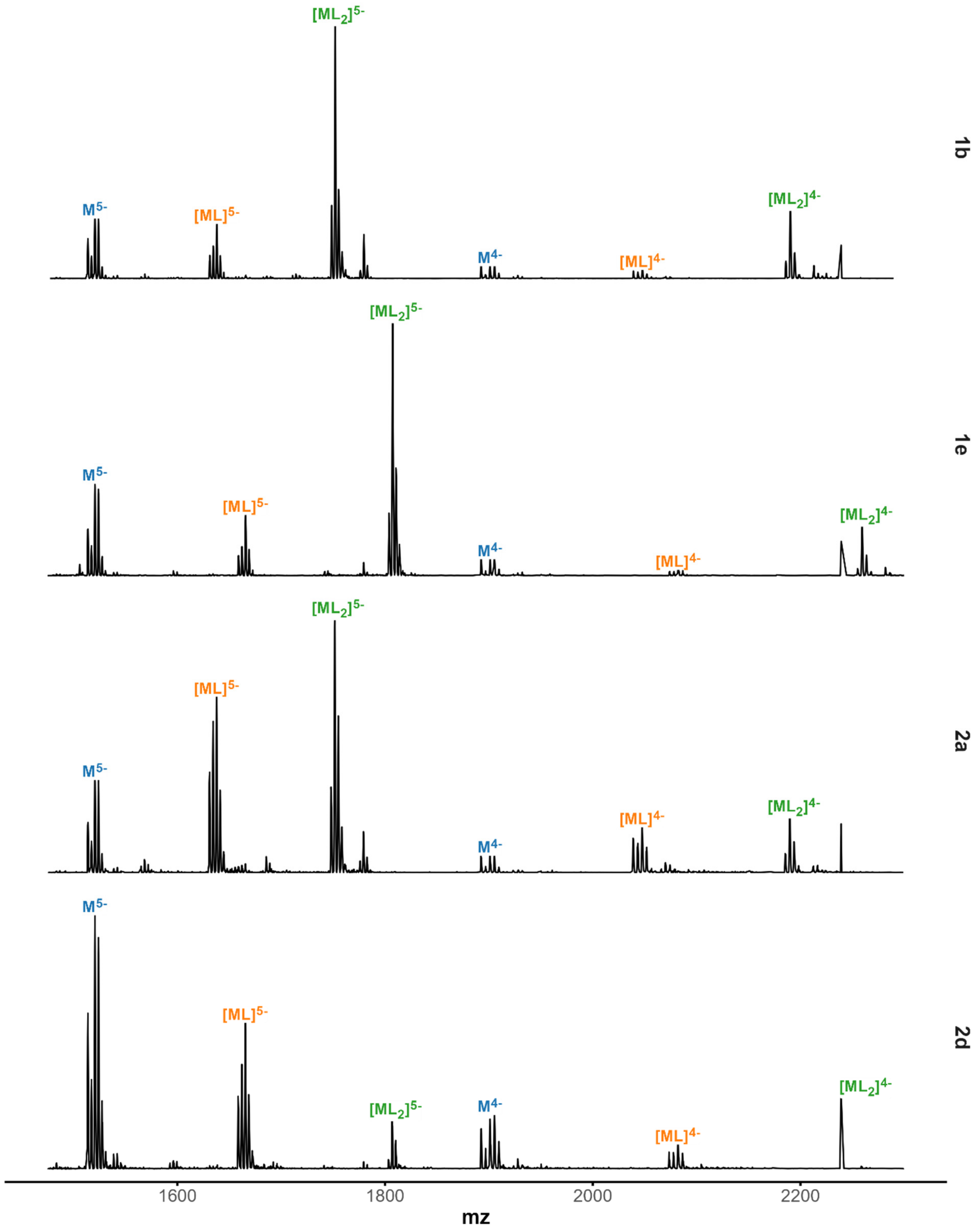

Figure S3. Native electrospray mass spectra of solutions containing **12b**, **12e**, **13a** or **13d** (20 μM) with 24TTG (10 μM) in 100 mM ammonium acetate (pH = 6.8), zoomed on the 4- and 5- charge states: M = oligonucleotide monomer, L = ligand.

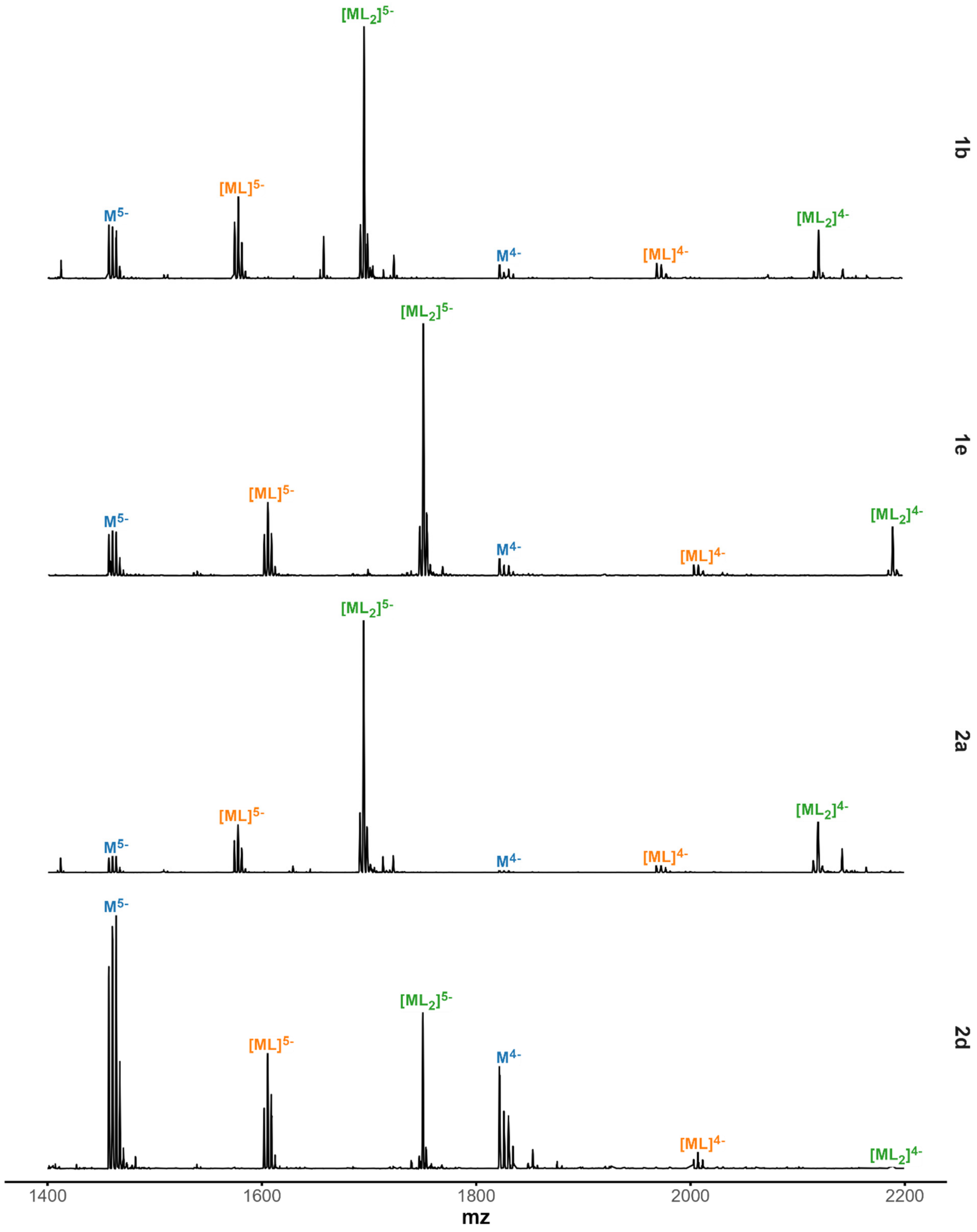

Figure S4. Native electrospray mass spectra of solutions containing **12b**, **12e**, **13a** or **13d** (20 μM) with BCL-2 (10 μM) in 100 mM ammonium acetate (pH = 6.8), zoomed on the 4- and 5- charge states: M = oligonucleotide monomer, L = ligand.

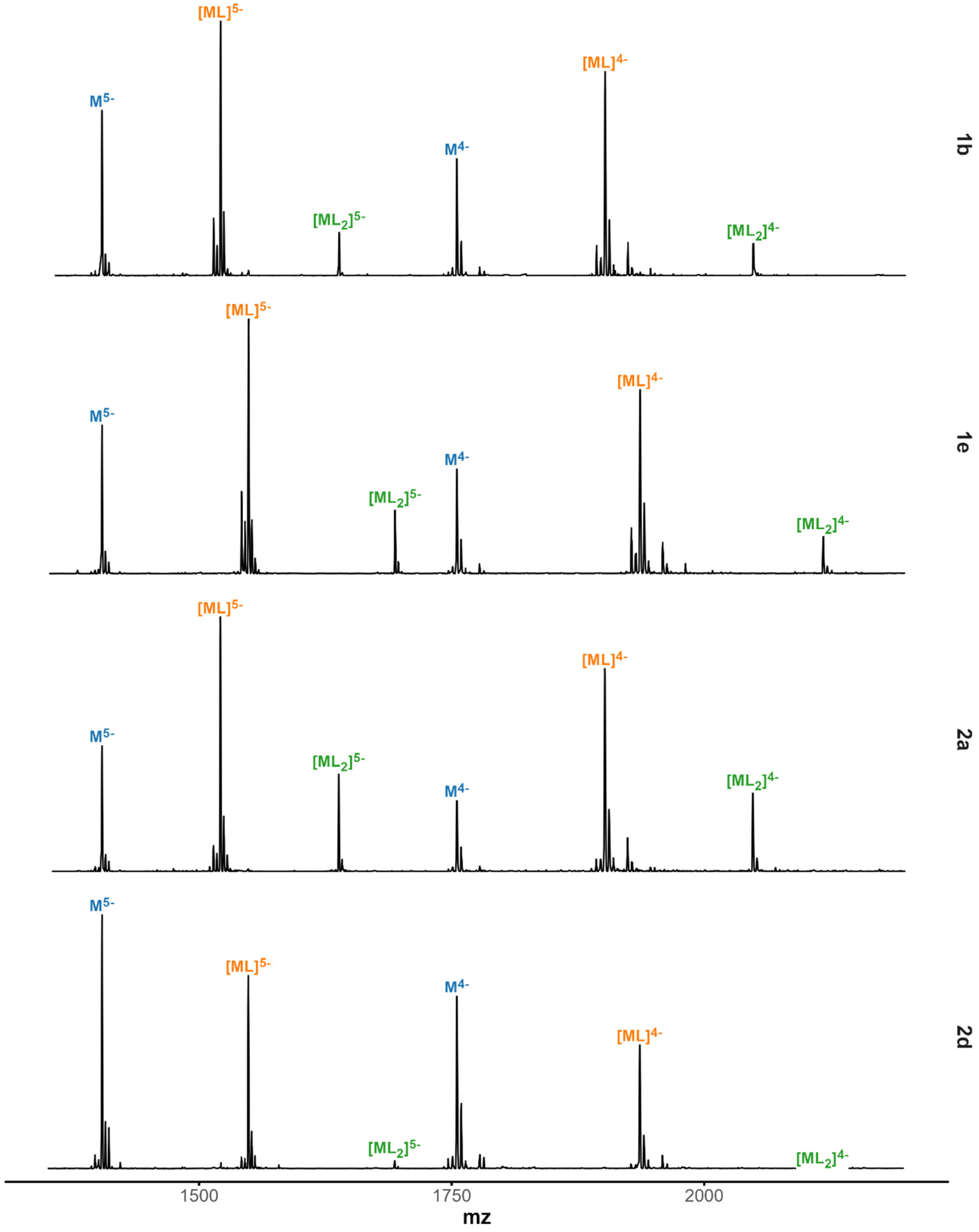

Figure S5. Native electrospray mass spectra of solutions containing **12b**, **12e**, **13a** or **13d** (20 µM) with c-MYC (10 µM) in 100 mM ammonium acetate (pH = 6.8), zoomed on the 4- and 5- charge states: M = oligonucleotide monomer, L = ligand.

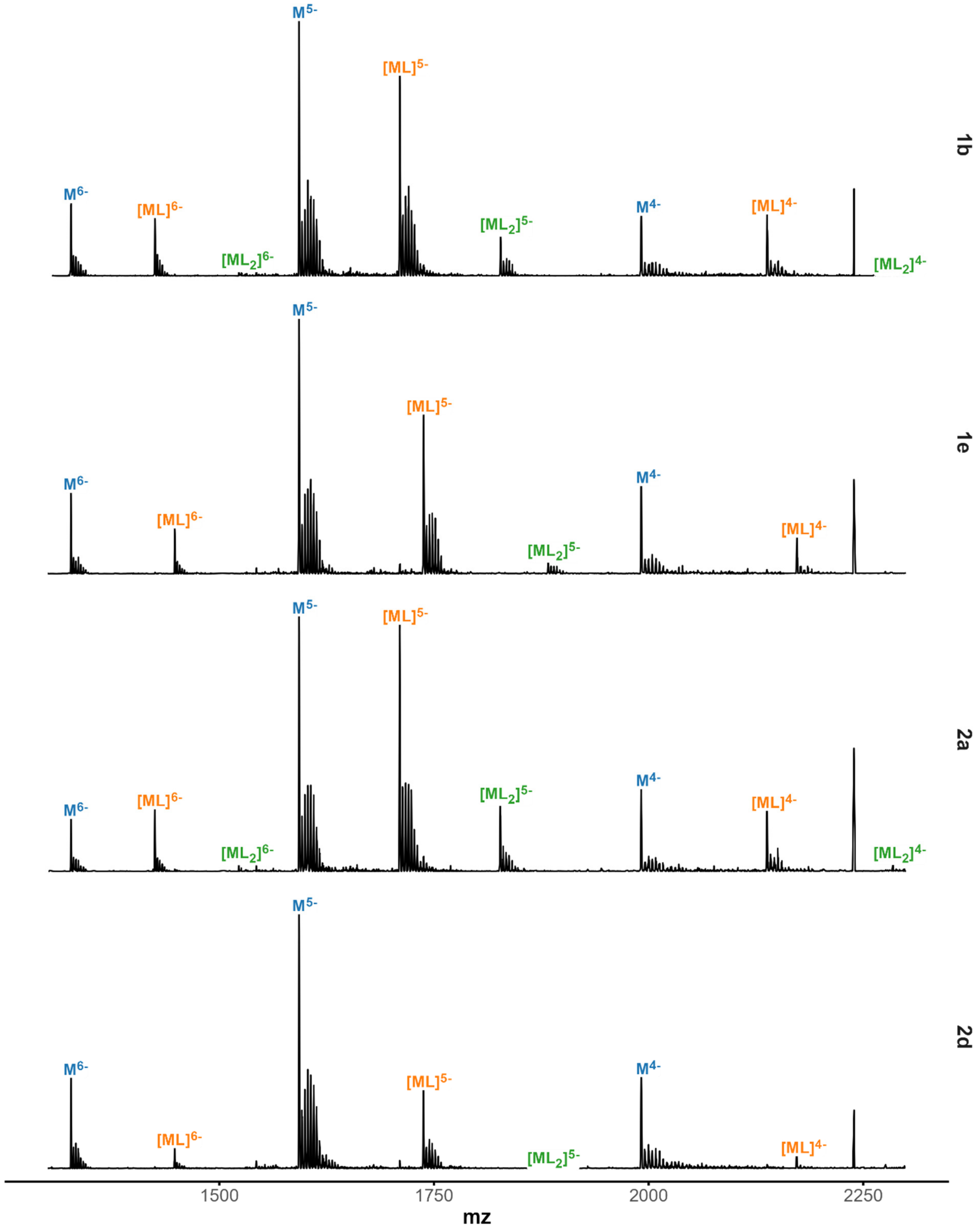

Figure S6. Native electrospray mass spectra of solutions containing **12b**, **12e**, **13a** or **13d** (20 µM) with ds26 (10 µM) in 100 mM ammonium acetate (pH = 6.8), zoomed on the 4-, 5- and 6- charge states: M = oligonucleotide monomer, L = ligand.

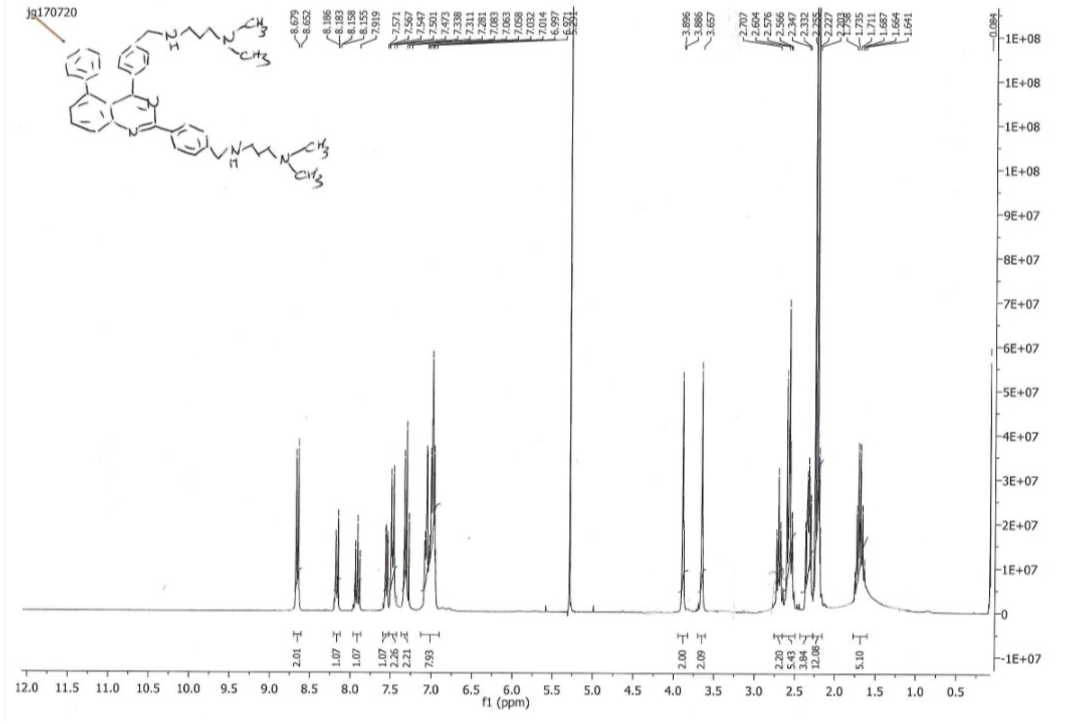

Figure S7. ^{1}H NMR spectrum of **12a**.

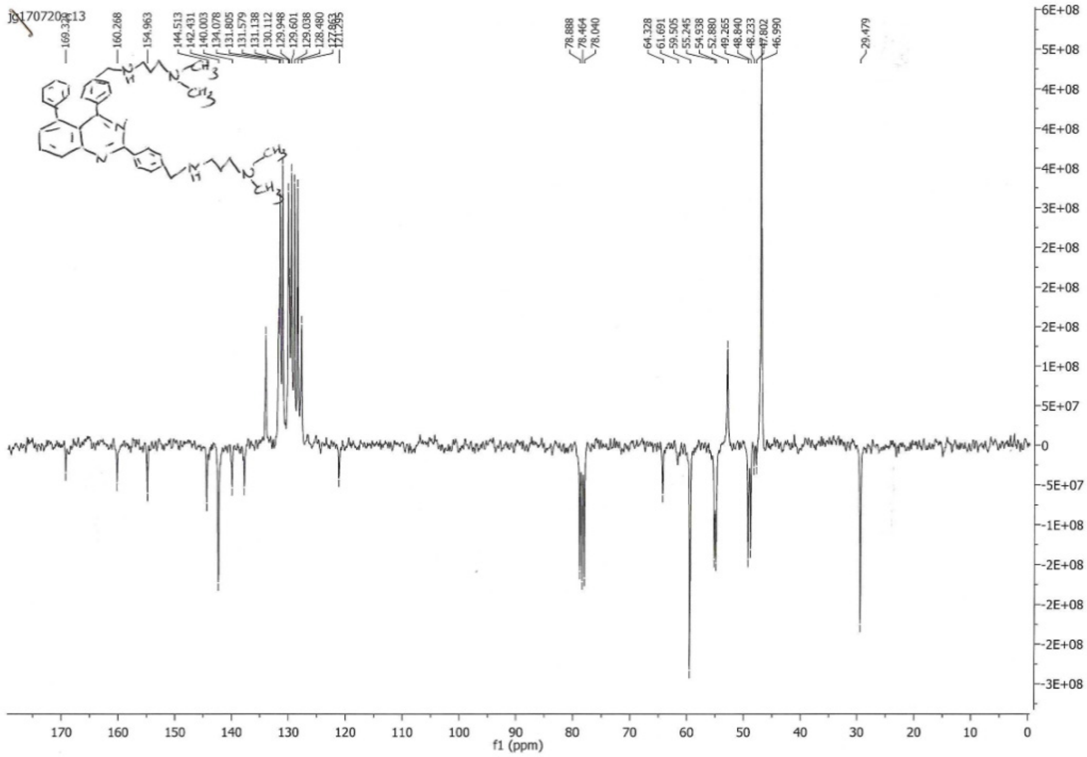

Figure S8. ^{13}C NMR spectrum of **12a**.

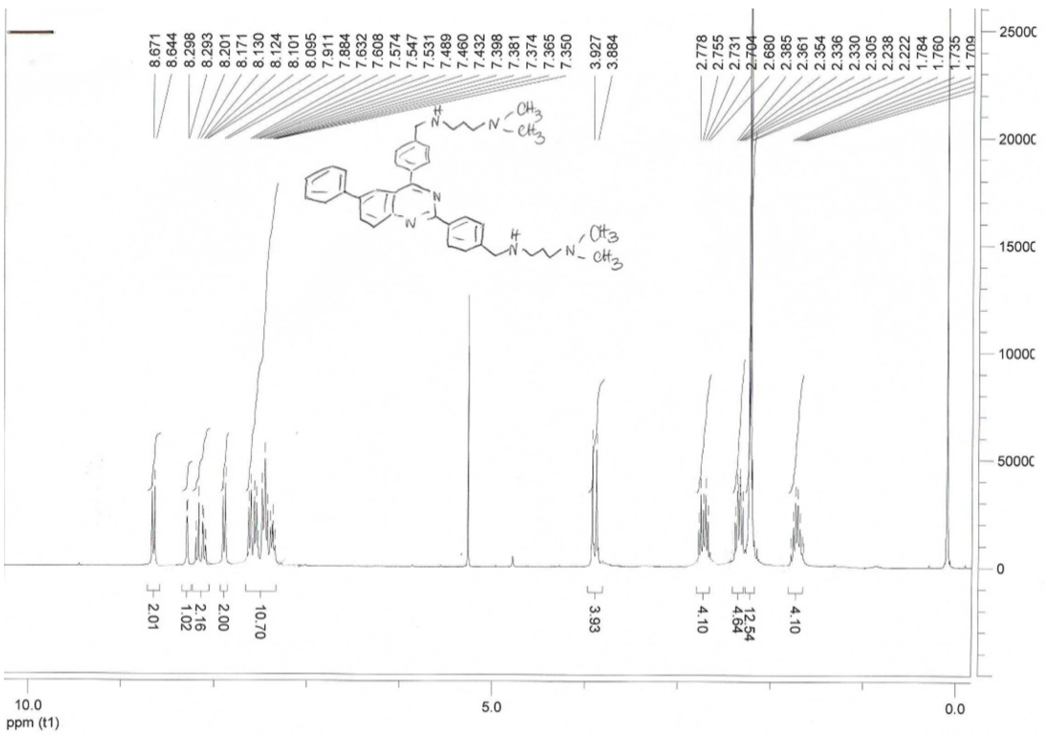

Figure S9. ^{1}H NMR spectrum of **12b**.

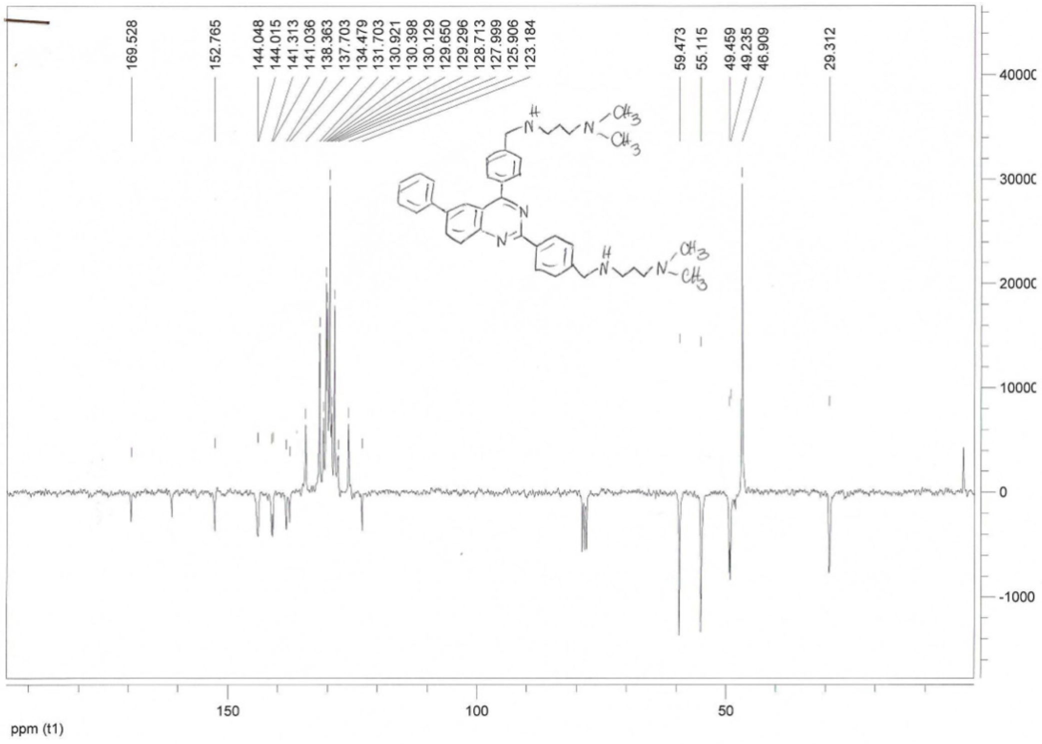

Figure S10. ^{13}C NMR spectrum of **12b**.

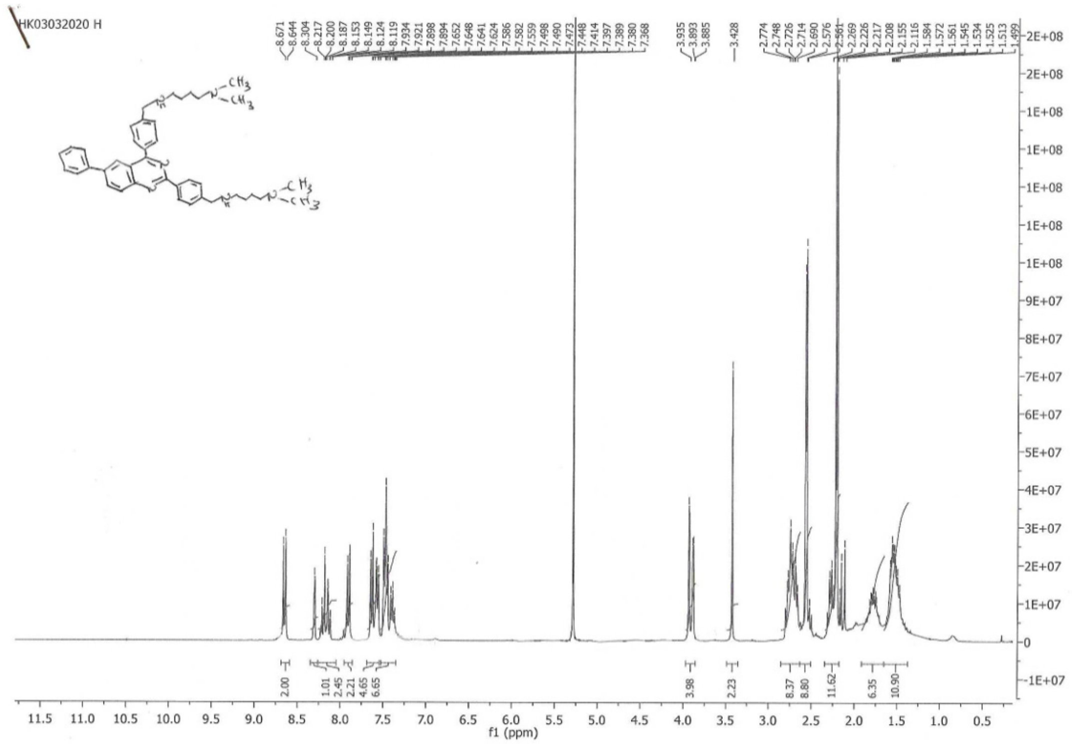

Figure S11. ^{1}H NMR spectrum of **12c**.

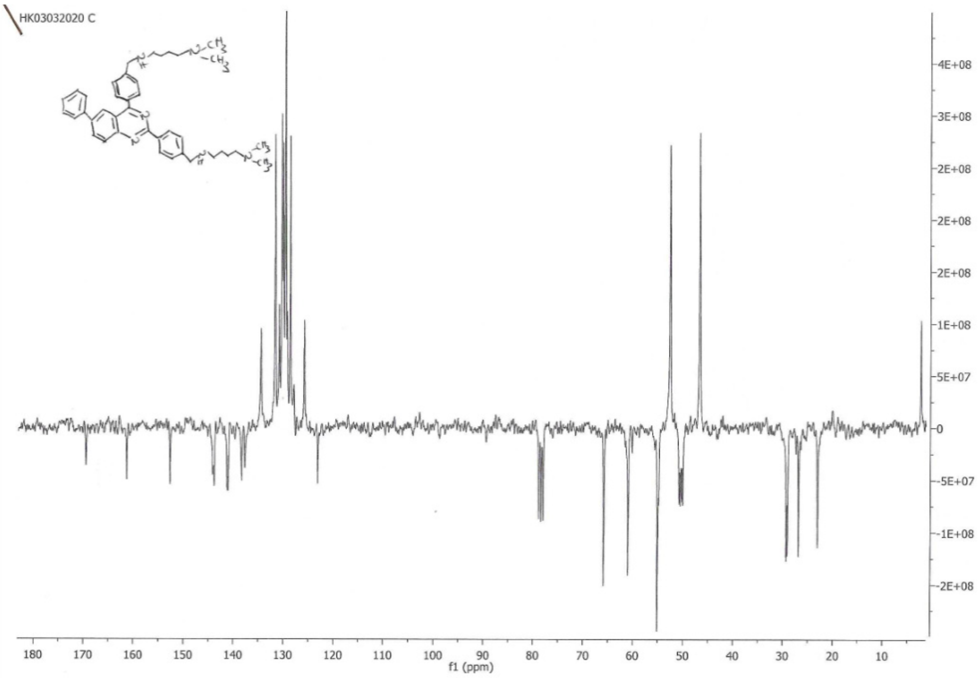

Figure S12. ^{13}C NMR spectrum of **12c**.

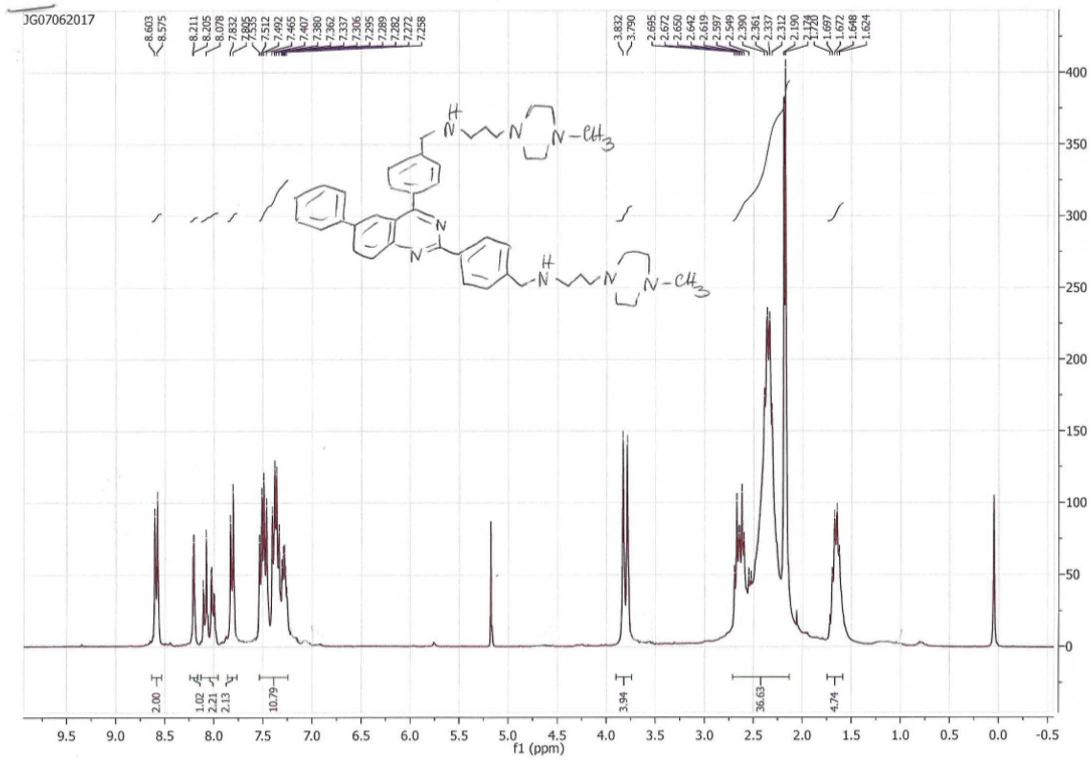

Figure S13. ^{1}H NMR spectrum of **12d**.

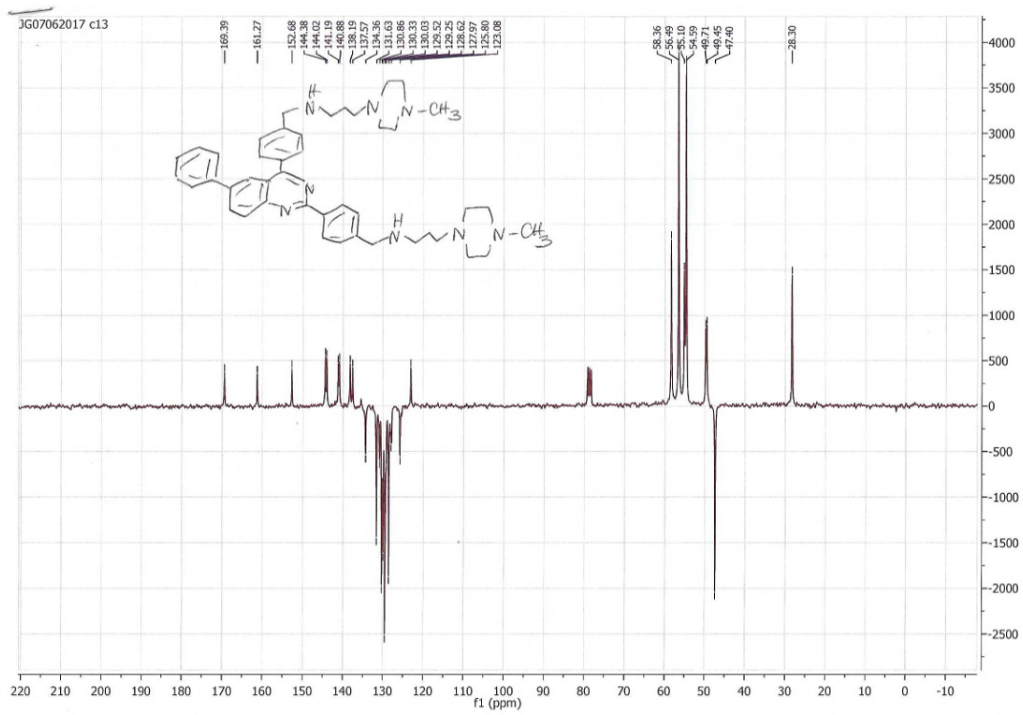

Figure S14. ^{13}C NMR spectrum of **12d**.

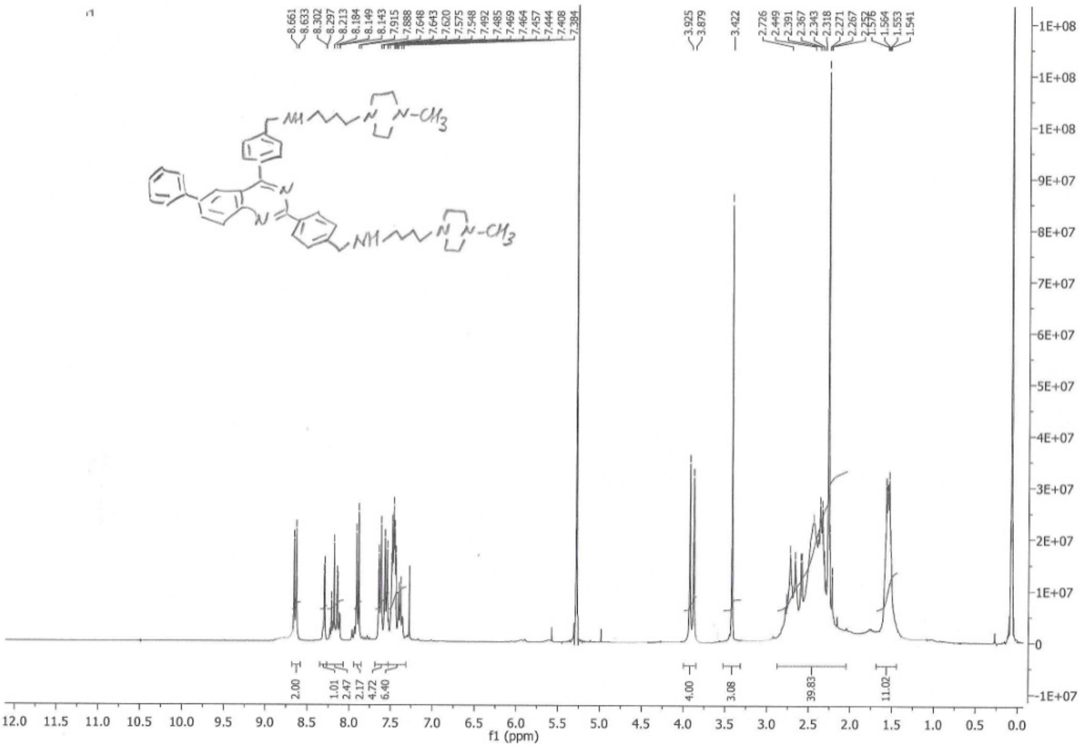

Figure S15. ^{1}H NMR spectrum of **12e**.

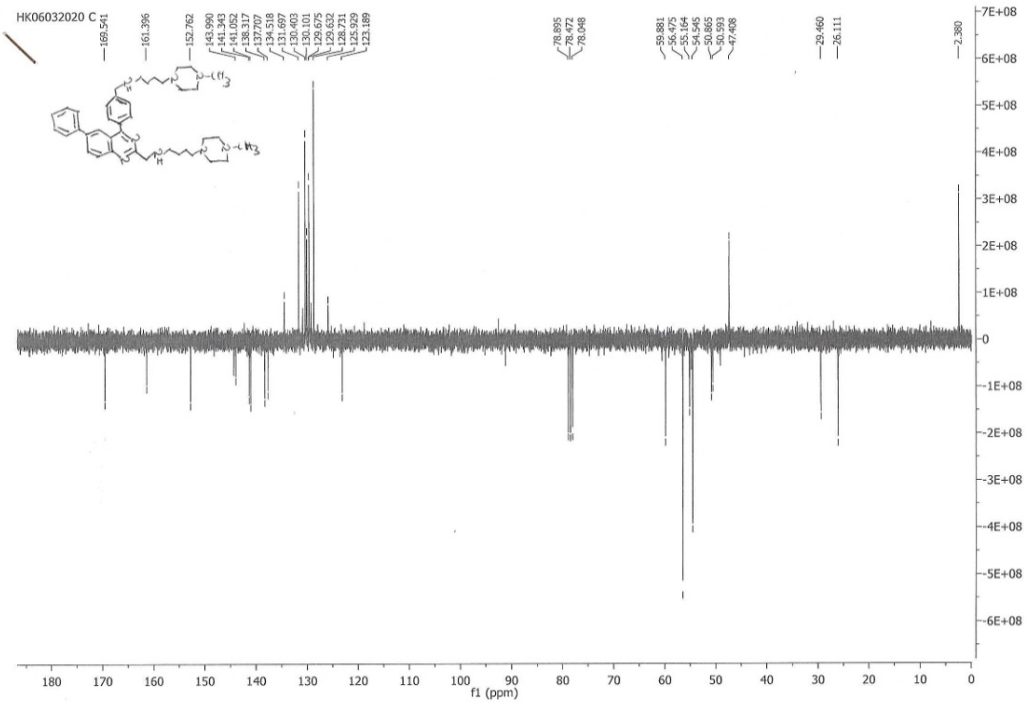

Figure S16. ^{13}C NMR spectrum of **12e**.

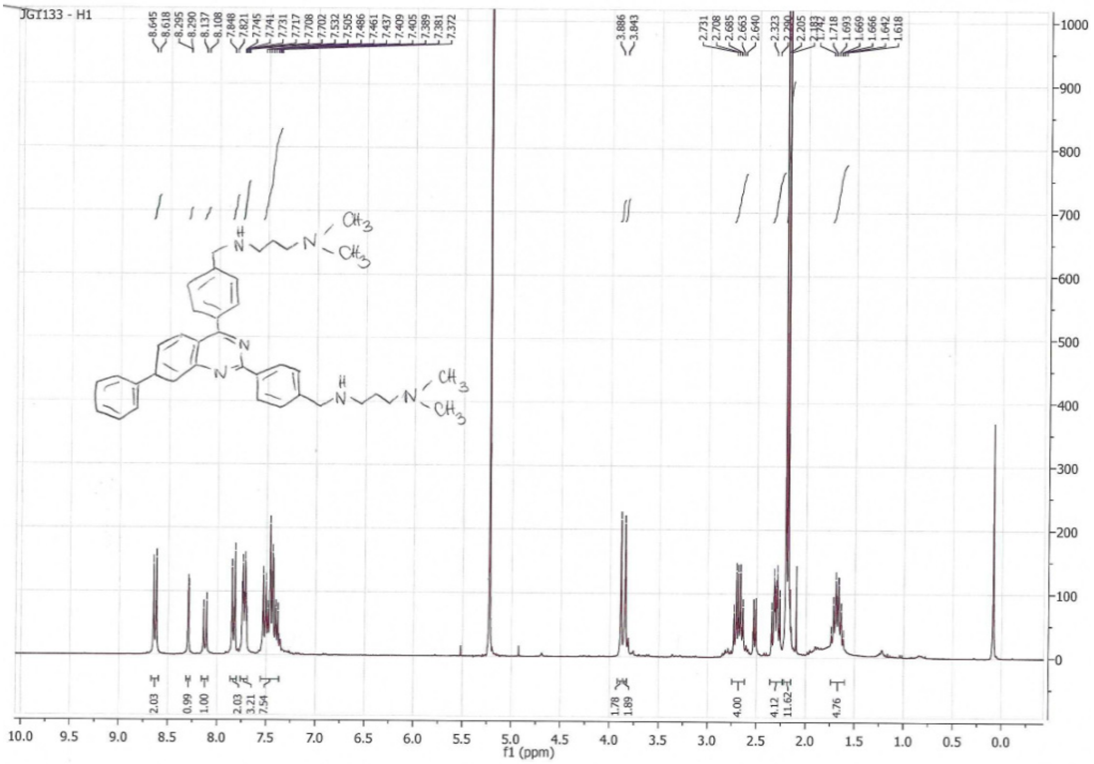

Figure S17. ^{1}H NMR spectrum of **12f**.

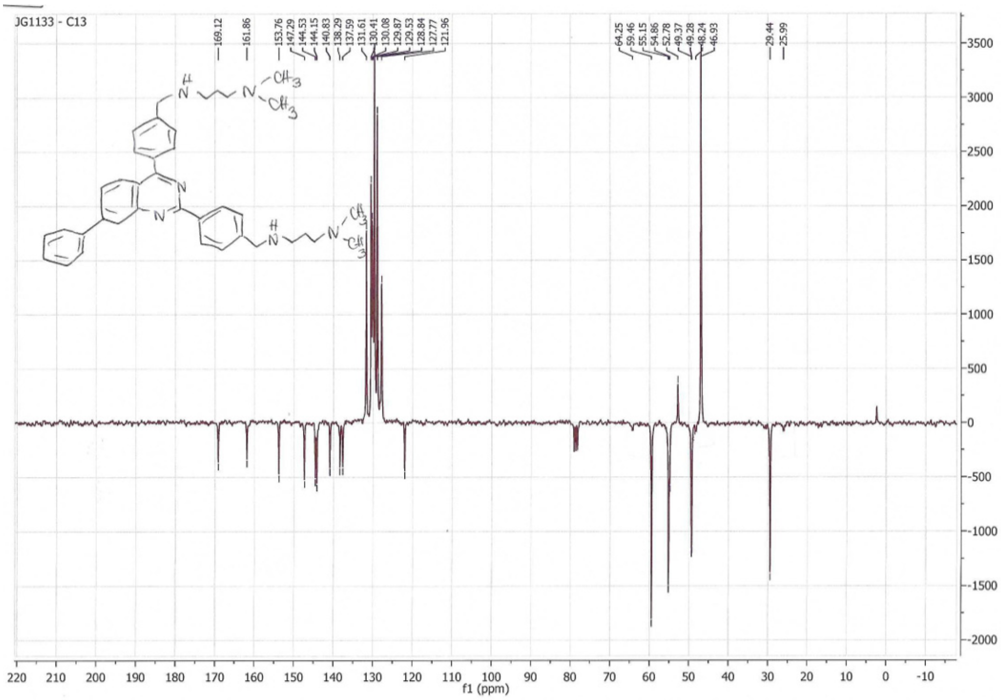

Figure S18. ^{13}C NMR spectrum of **12f**.

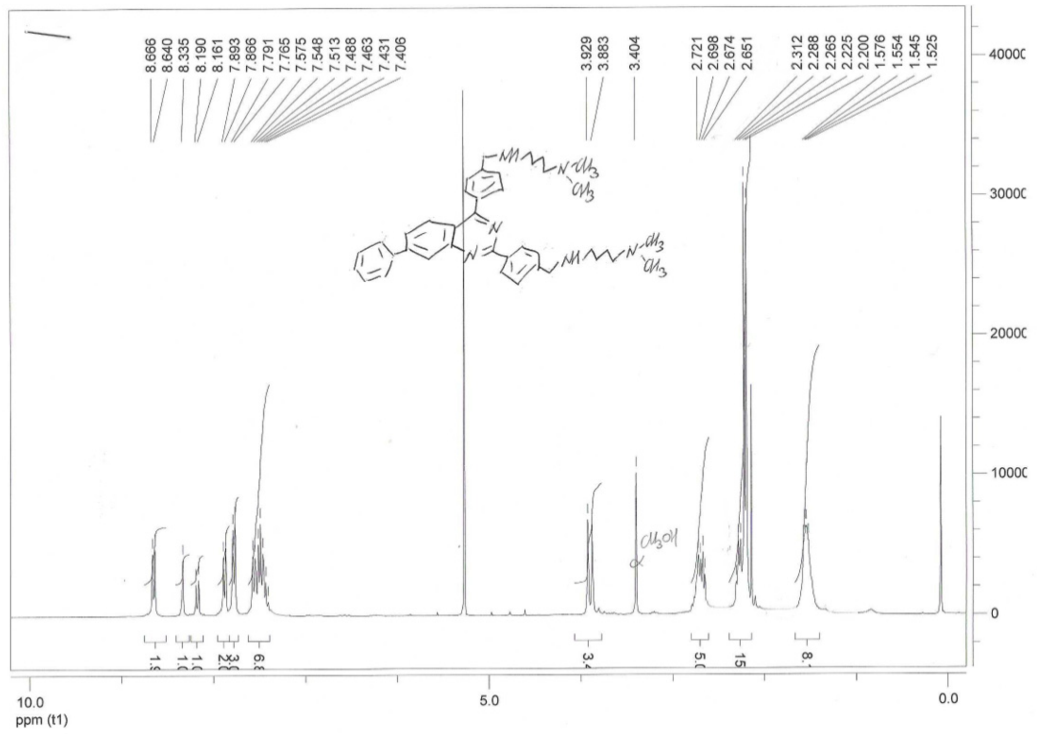

Figure S19. ^{1}H NMR spectrum of **12g**.

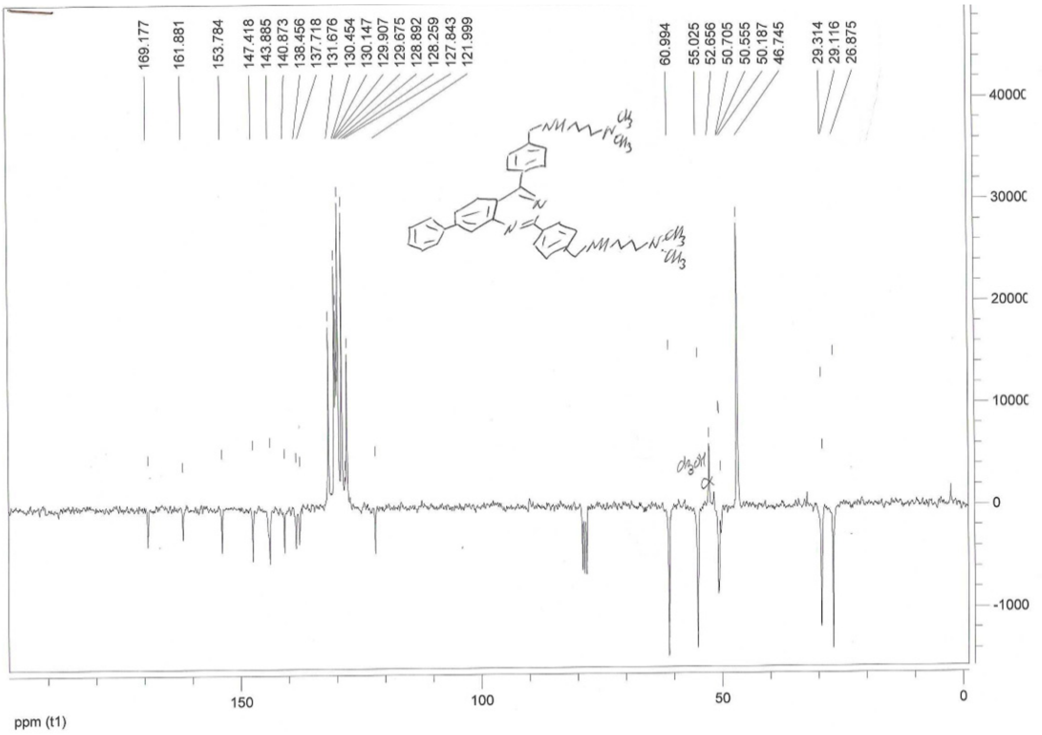

Figure S20. ^{13}C NMR spectrum of **12g**.

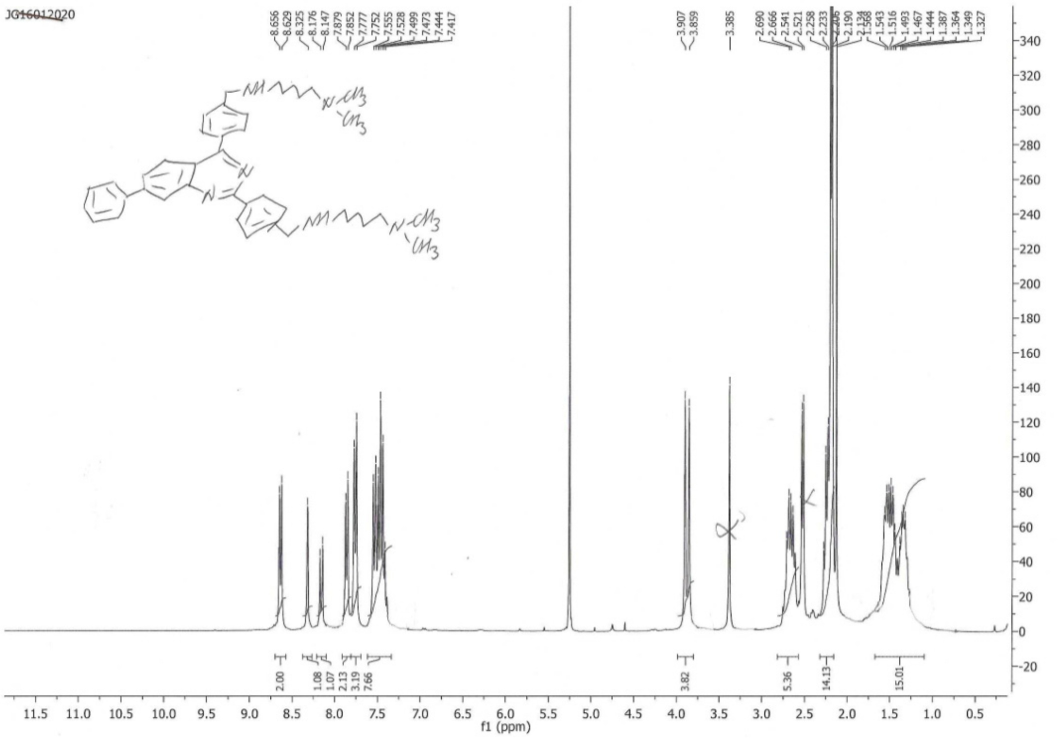

Figure S21. ^{1}H NMR spectrum of **12h**.

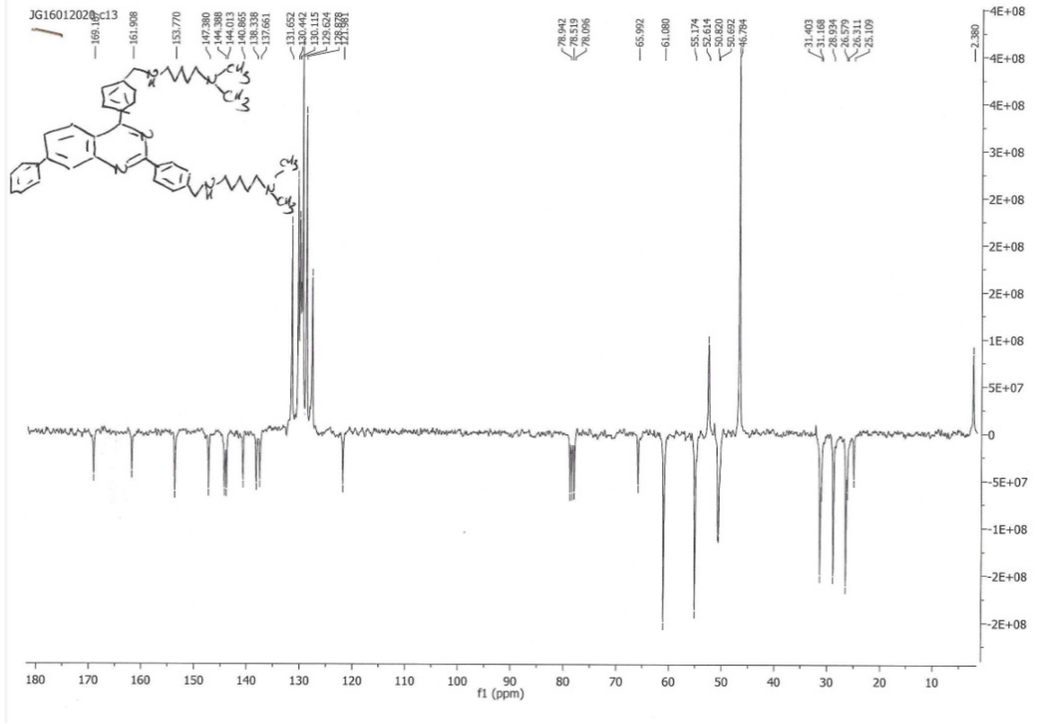

Figure S22. ^{13}C NMR spectrum of **12h**.

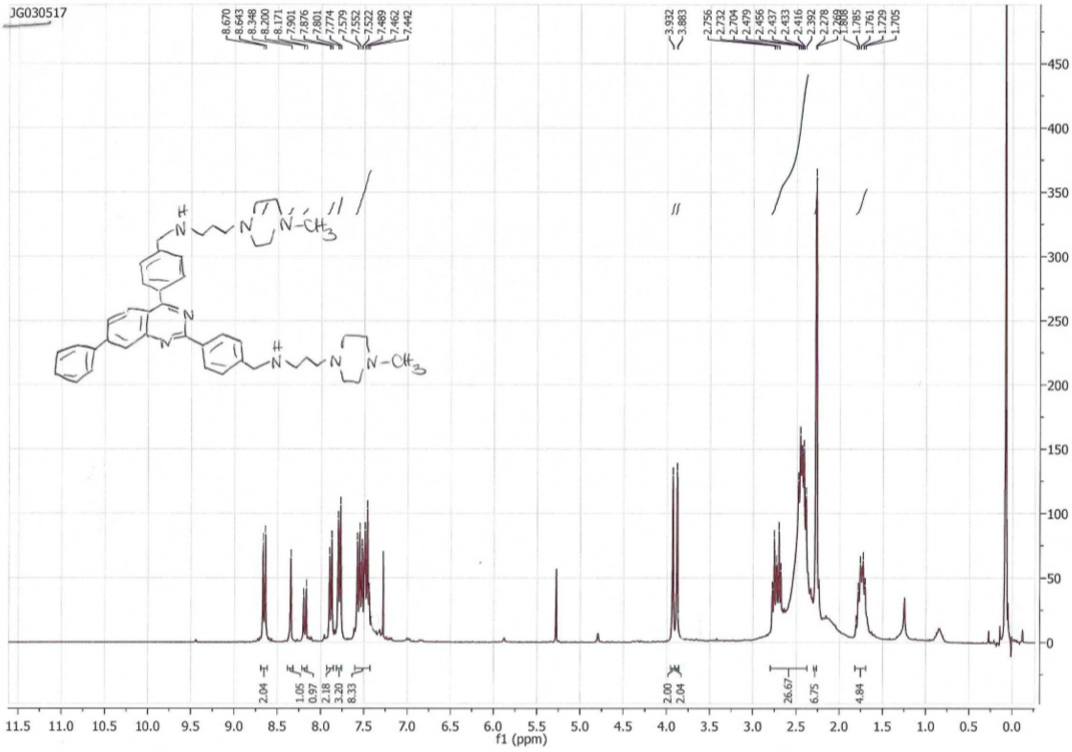

Figure S23. ^{1}H NMR spectrum of **12i**.

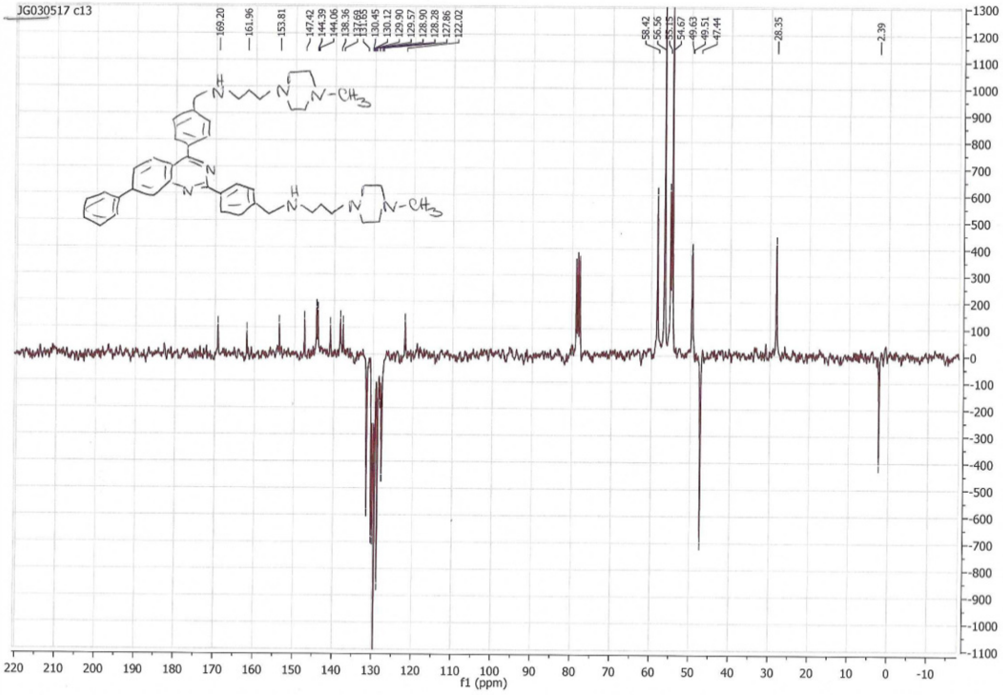

Figure S24. ^{13}C NMR spectrum of **12i**.

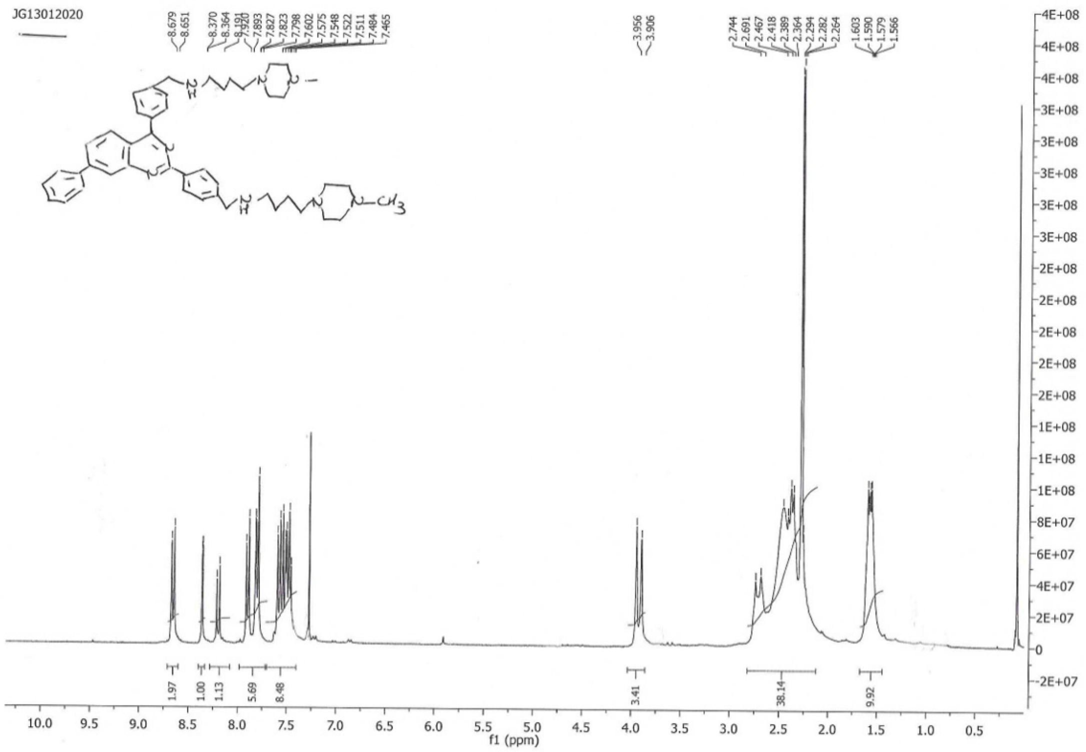

Figure S25. ^{1}H NMR spectrum of **12j**.

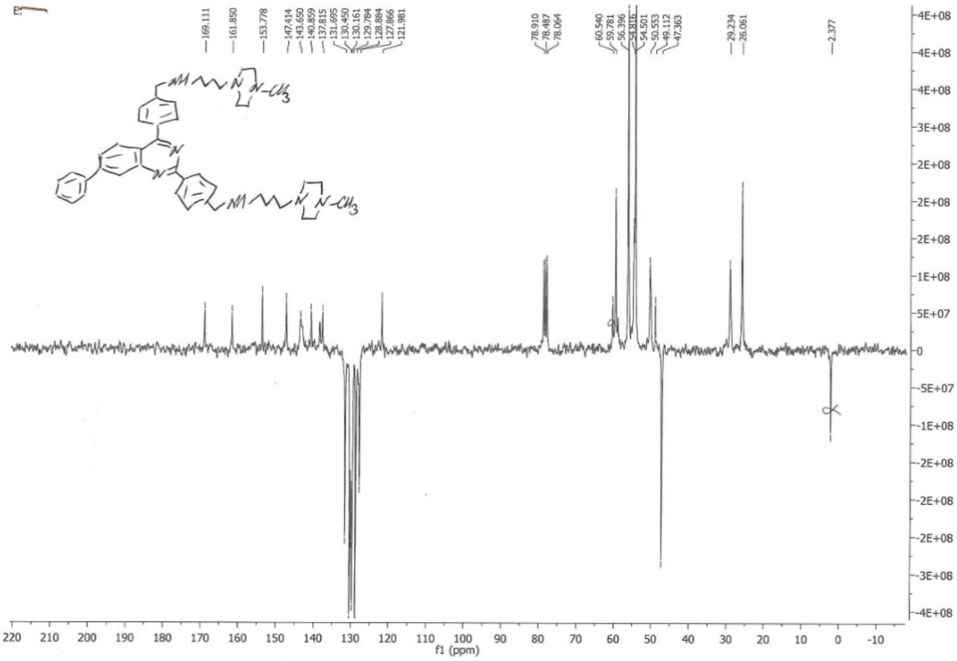

Figure S26. ^{13}C NMR spectrum of **12j**.

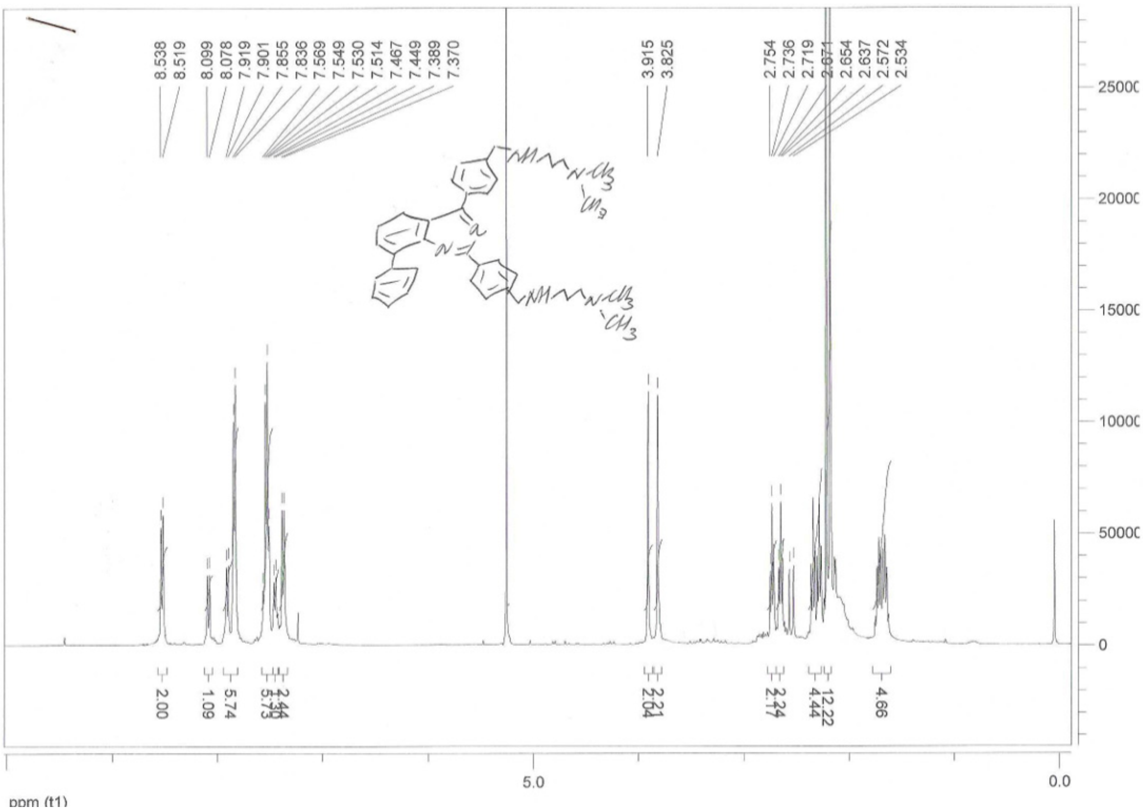

Figure S27. ^{1}H NMR spectrum of **12k**.

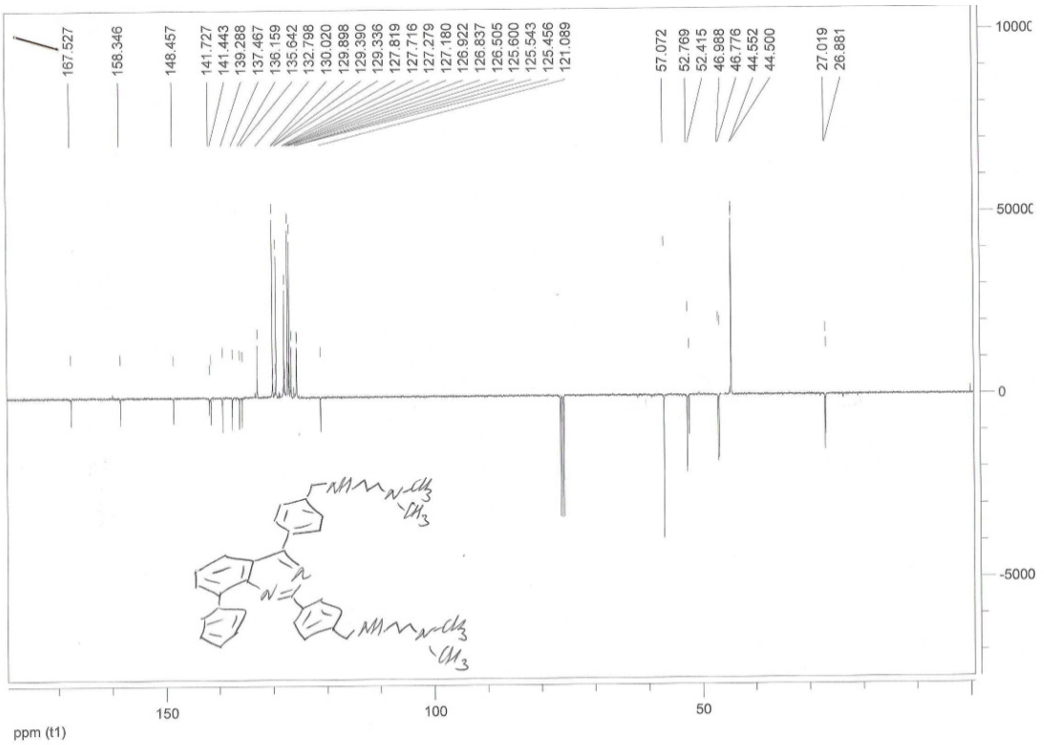

Figure S28. ^{13}C NMR spectrum of **12k**.

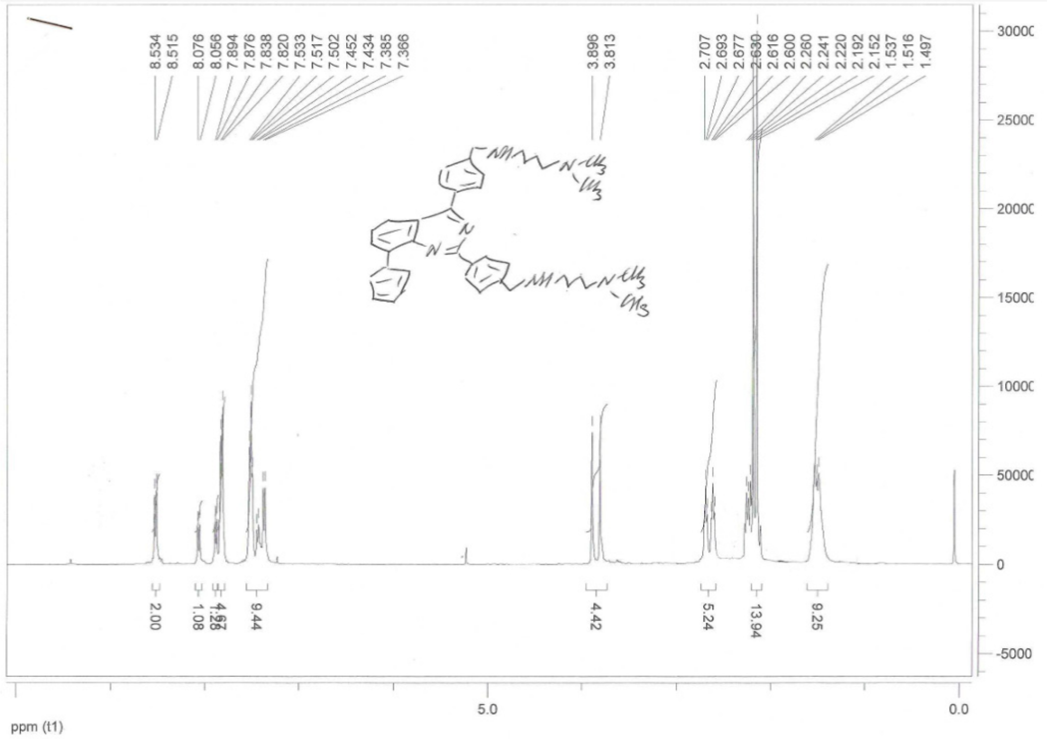

Figure S29. ^{1}H NMR spectrum of **12l**.

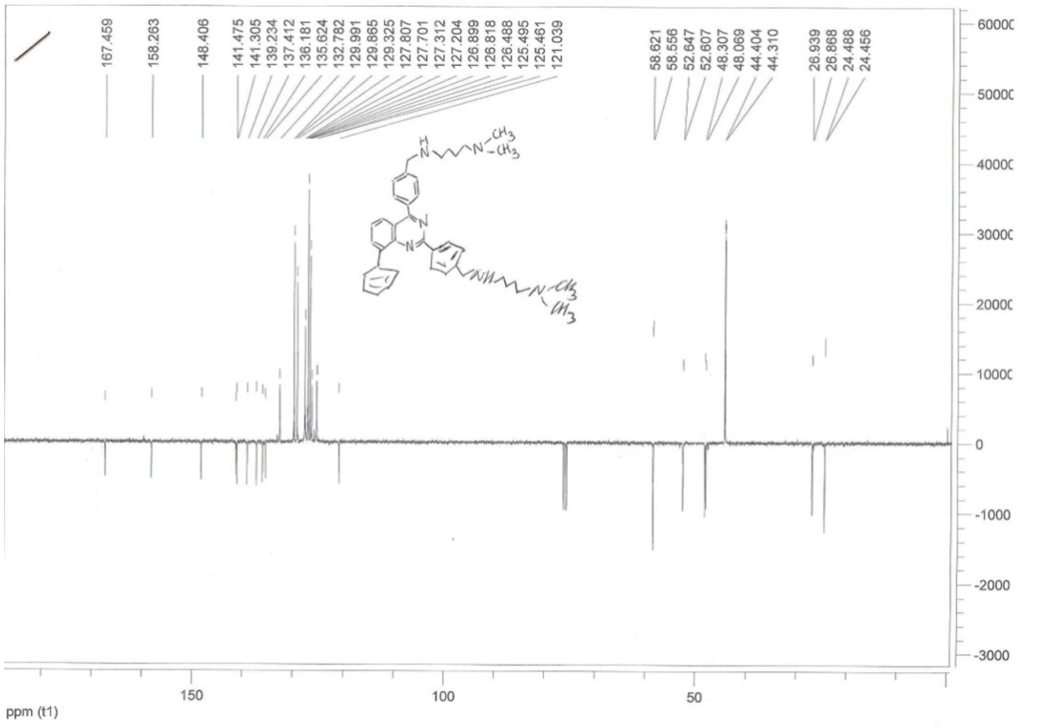

Figure S30. ^{13}C NMR spectrum of **12l**.

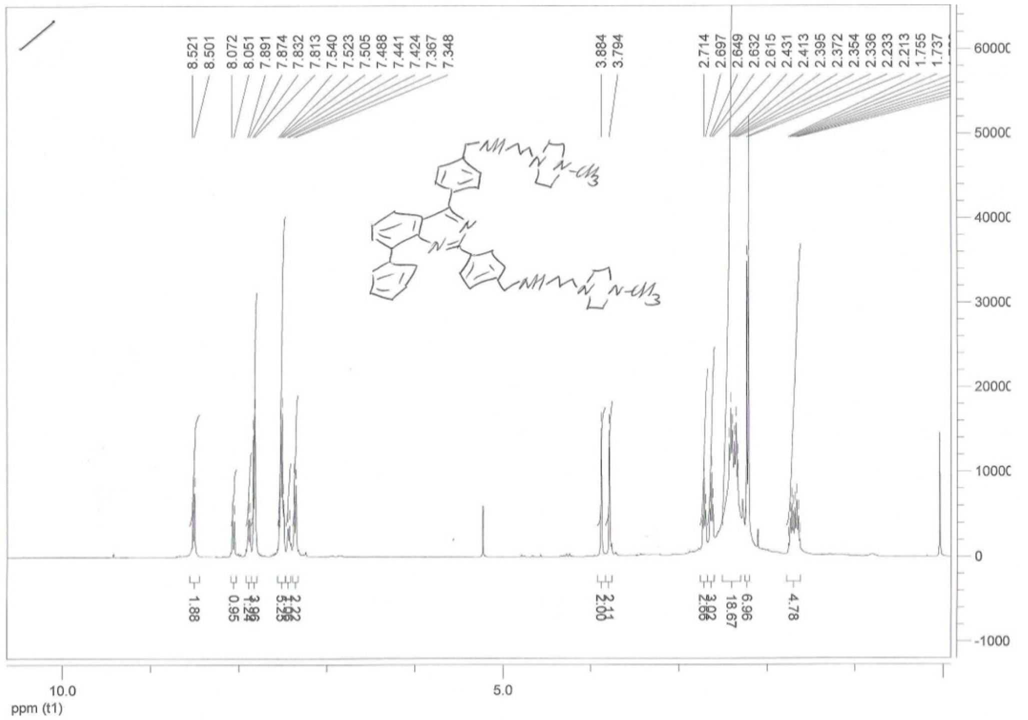

Figure S31. ^{1}H NMR spectrum of **12m**.

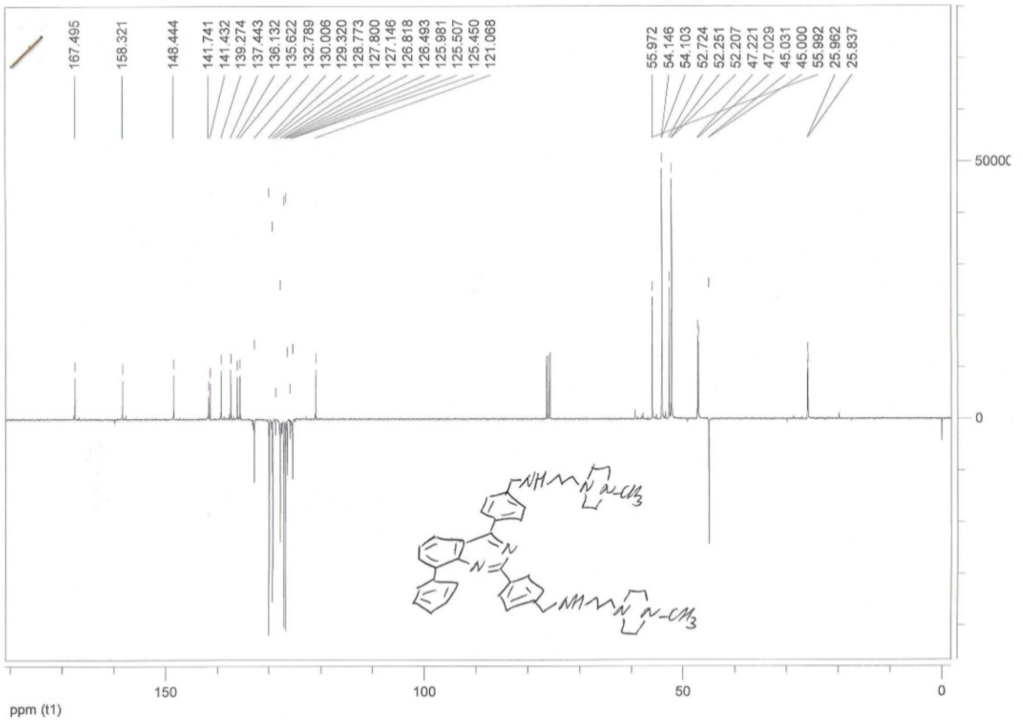

Figure S32. ^{13}C NMR spectrum of **12m**.

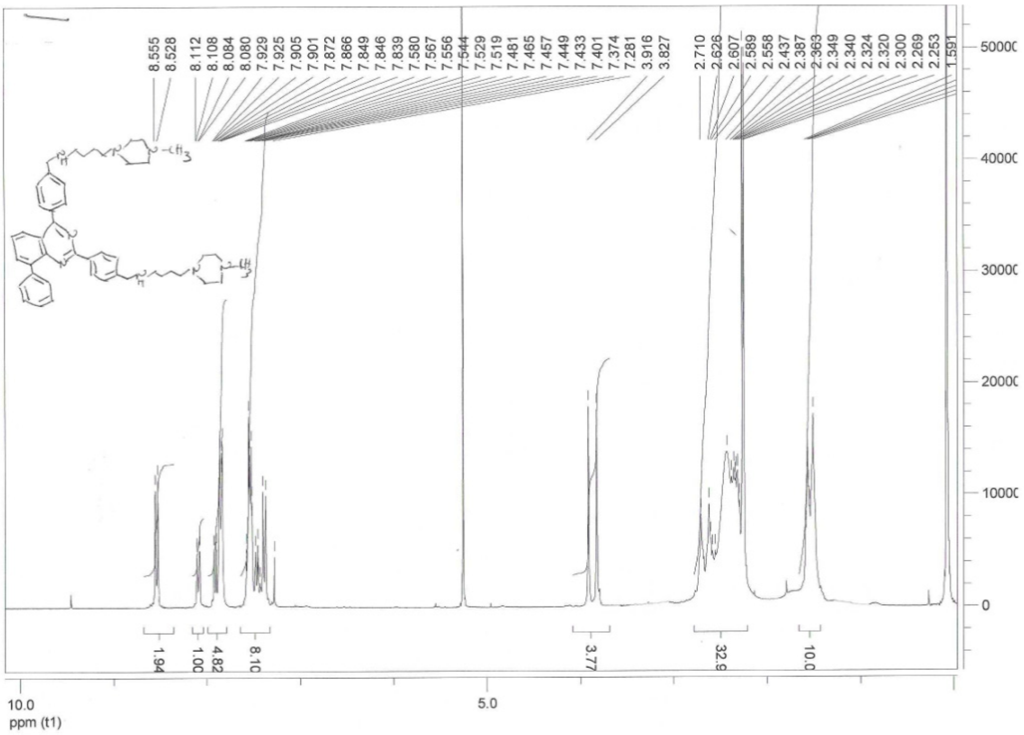

Figure S33. ^{1}H NMR spectrum of **12n**.

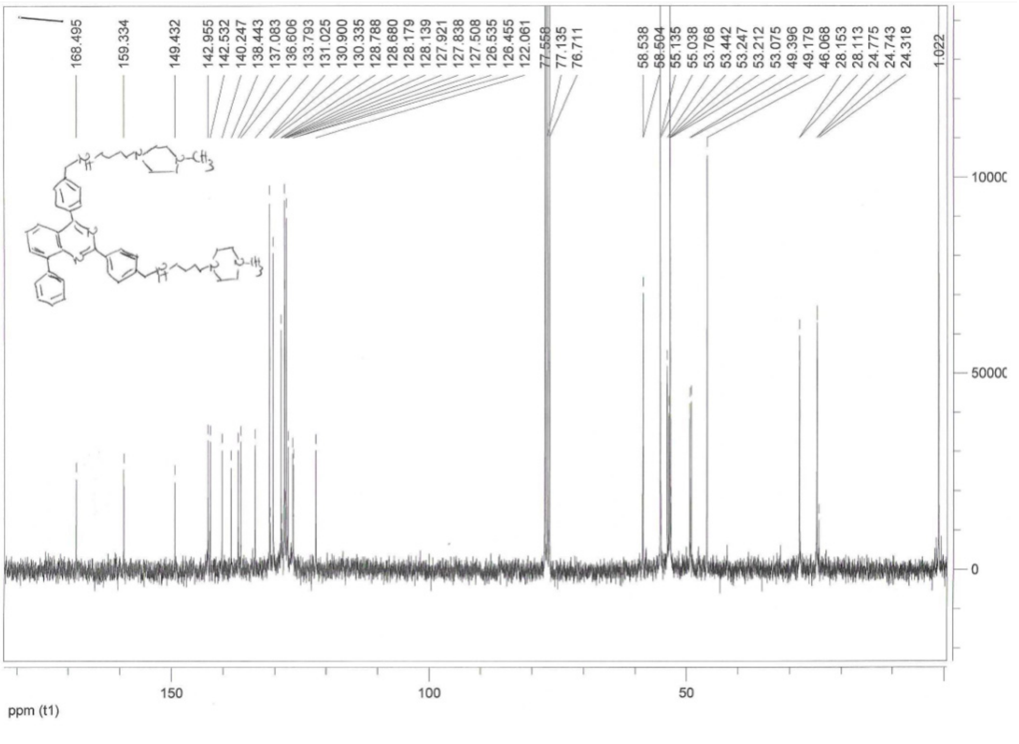

Figure S34. ^{13}C NMR spectrum of **12n**.

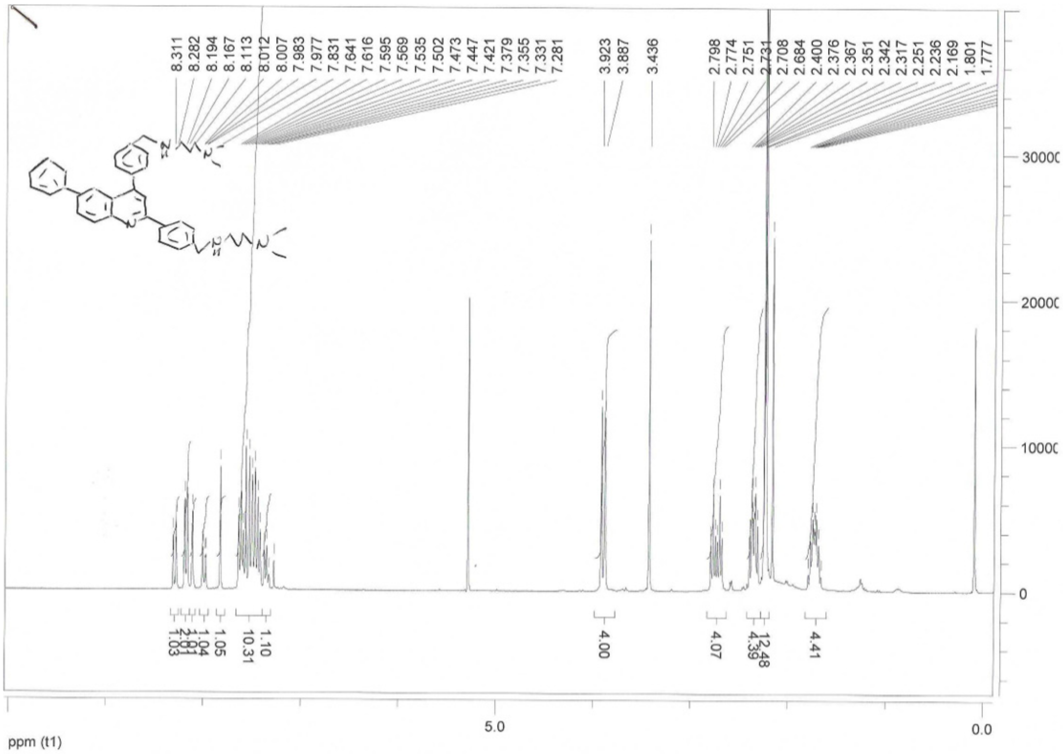

Figure S35. ^{1}H NMR spectrum of **13a**.

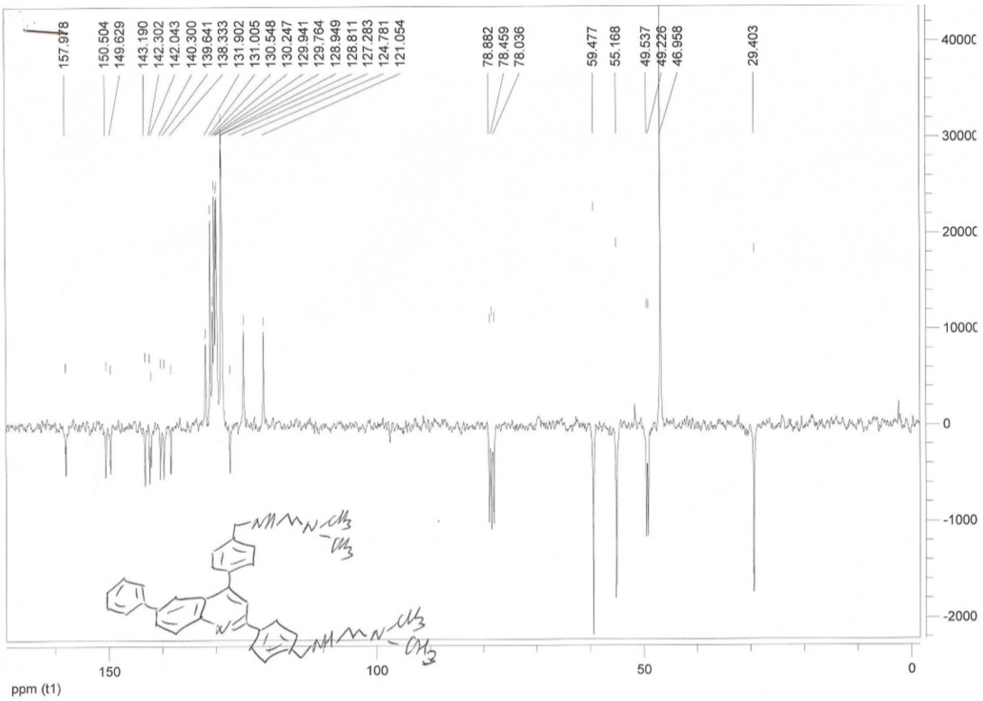

Figure S36. ^{13}C NMR spectrum of **13a**.

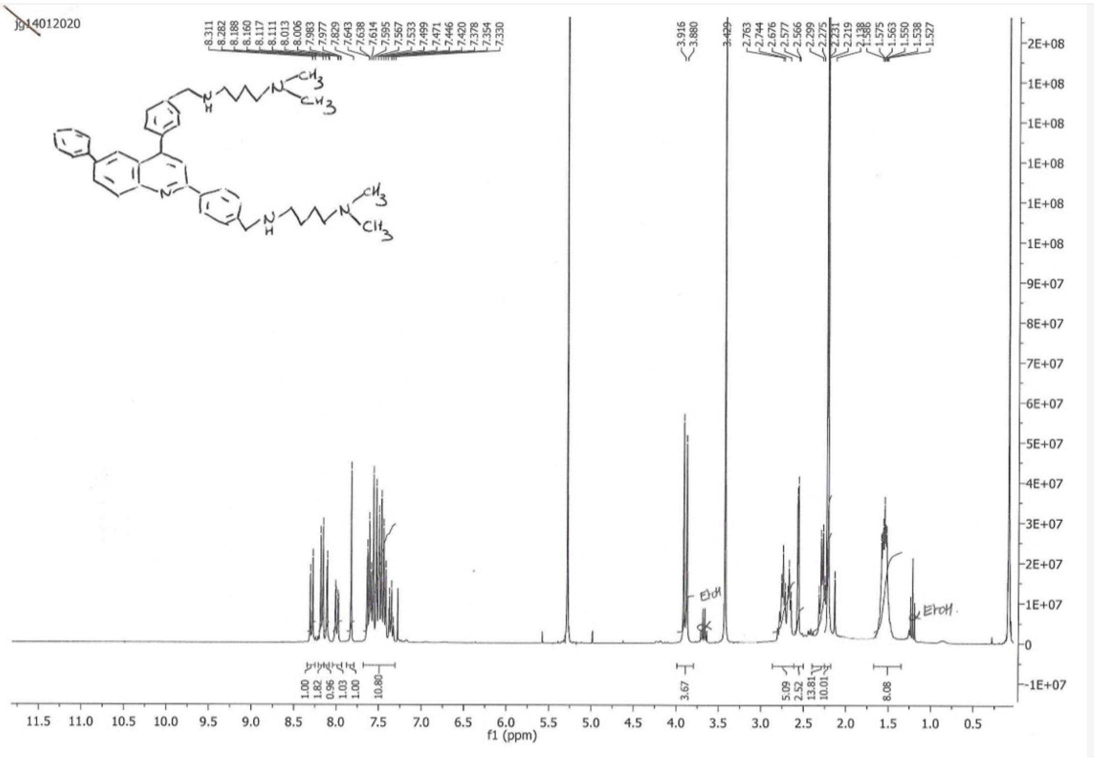

Figure S37. ^{1}H NMR spectrum of **13b**.

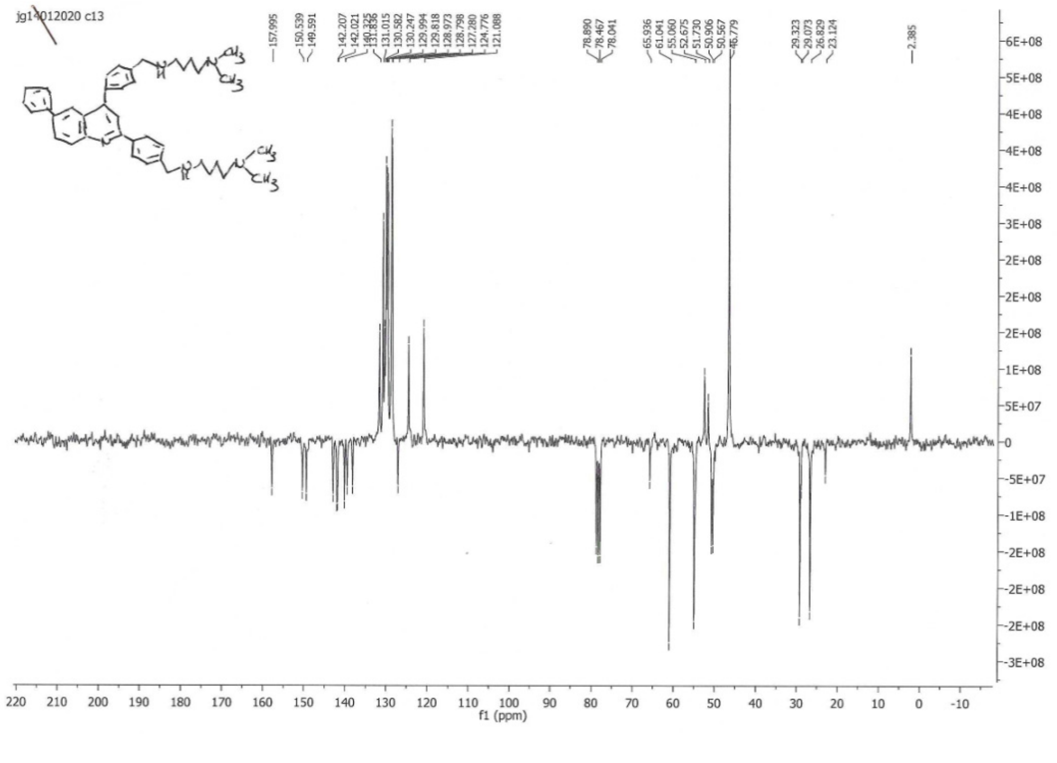

Figure S38. ^{13}C NMR spectrum of **13b**.

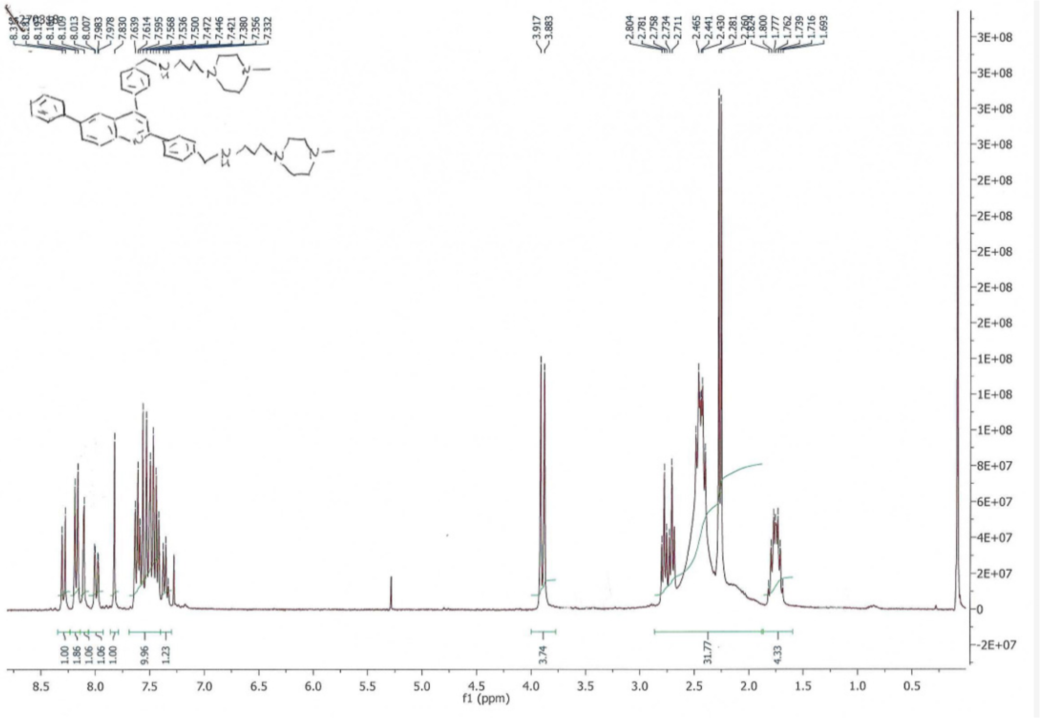

Figure S39. ^{1}H NMR spectrum of **13c**.

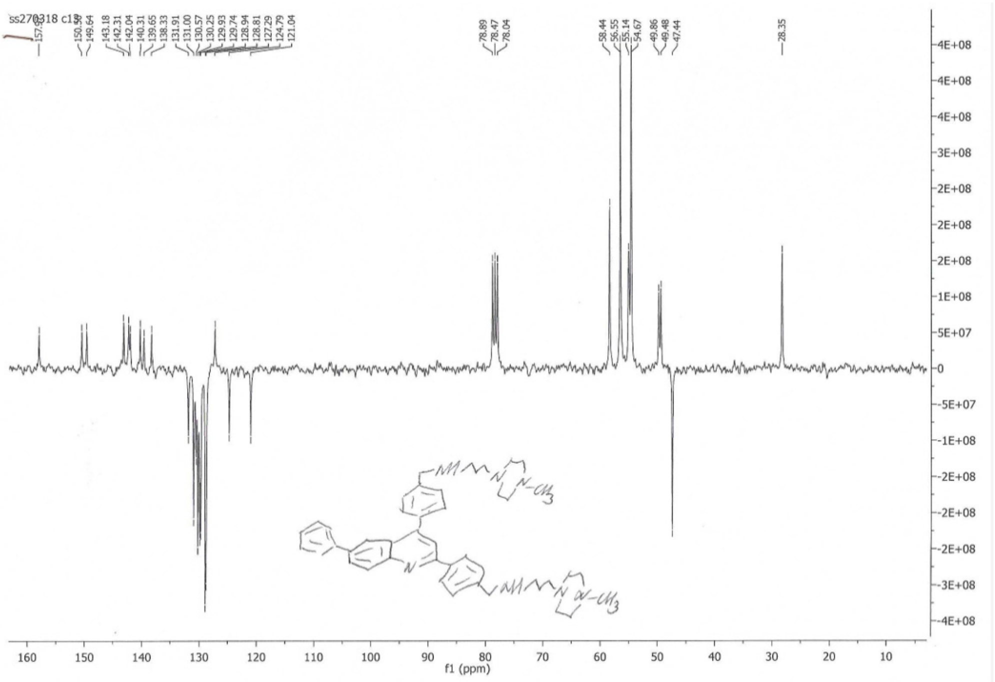

Figure S40. ^{13}C NMR spectrum of **13c**.

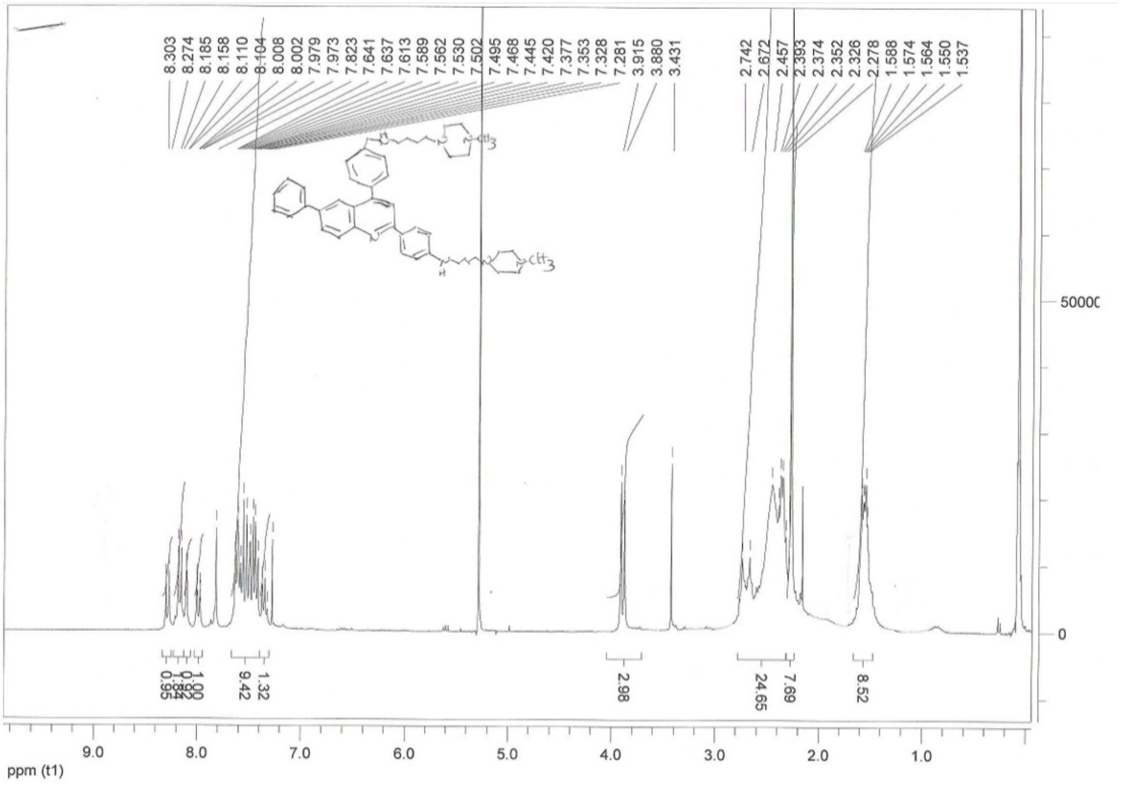

Figure S41. ^{1}H NMR spectrum of **13d**.

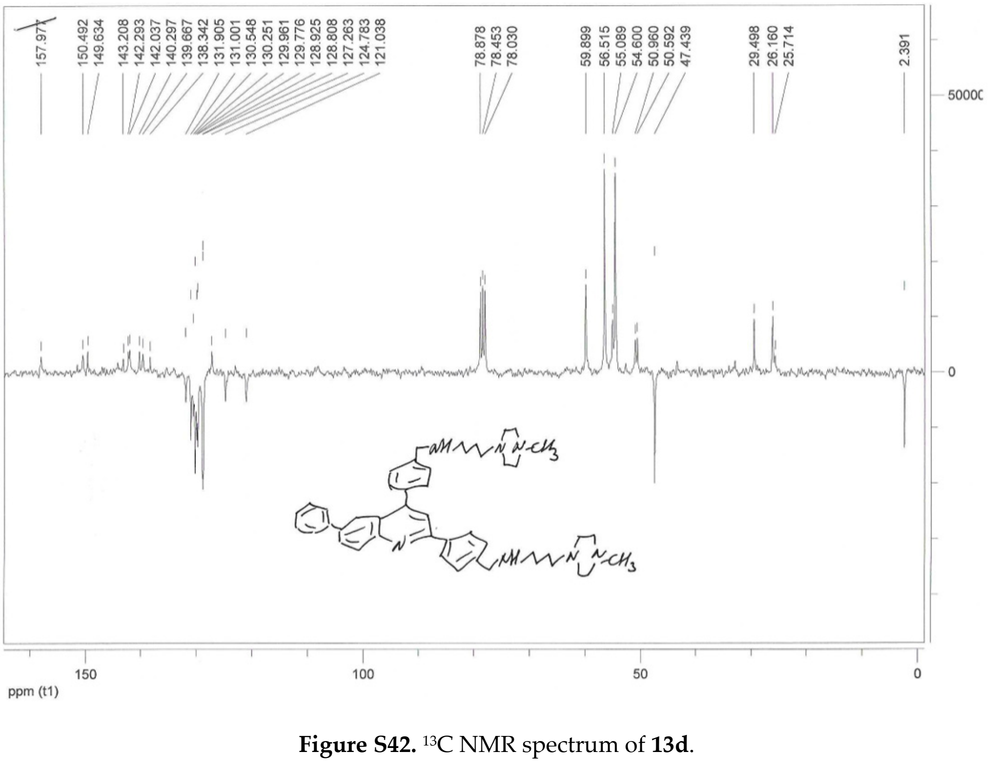

Figure S42. ^{13}C NMR spectrum of **13d**.

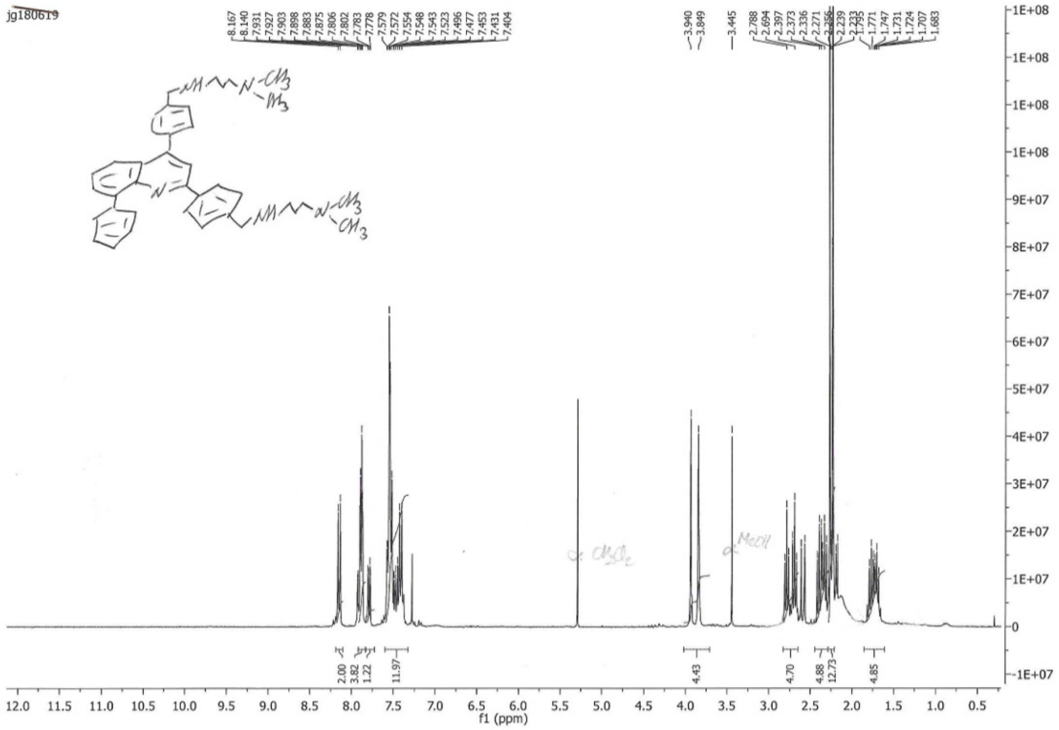

Figure S43. ^{1}H NMR spectrum of **13e**.

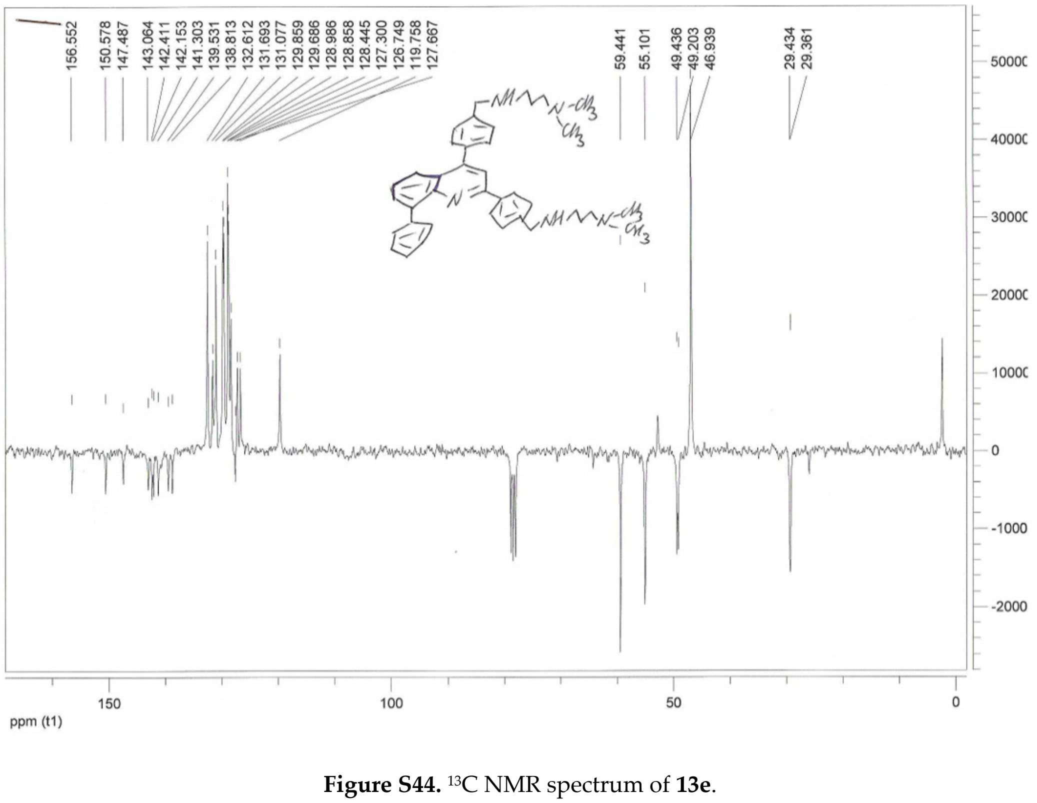

Figure S44. ^{13}C NMR spectrum of **13e**.

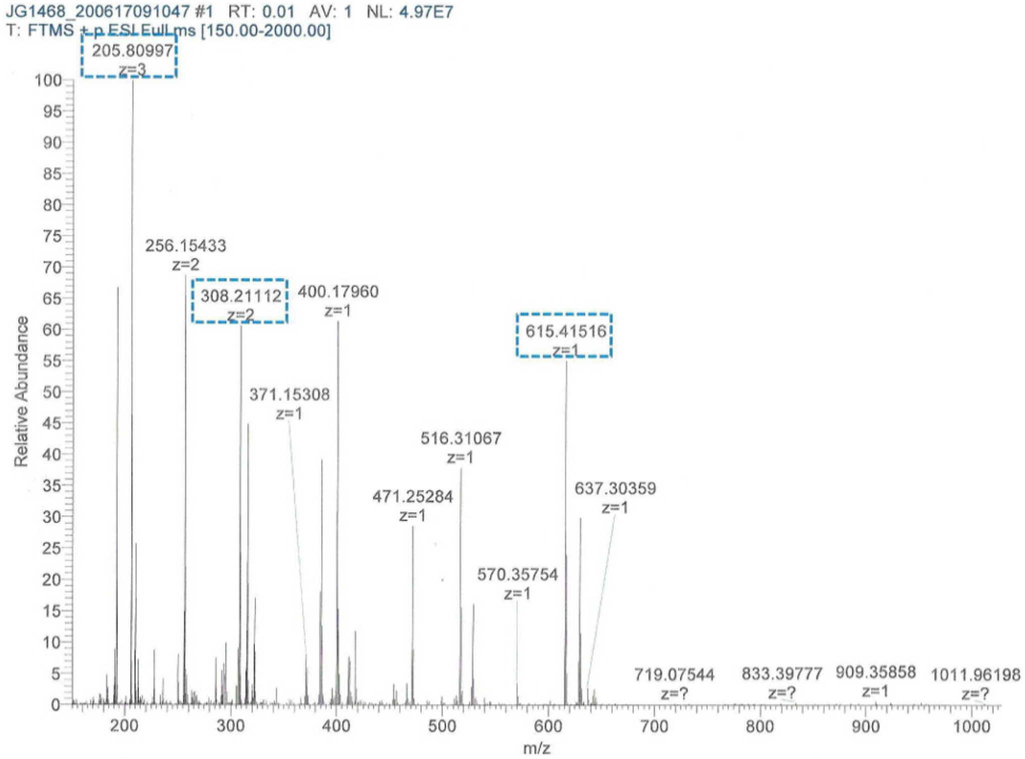

Figure S45. ESI-MS data for compound **12a**.

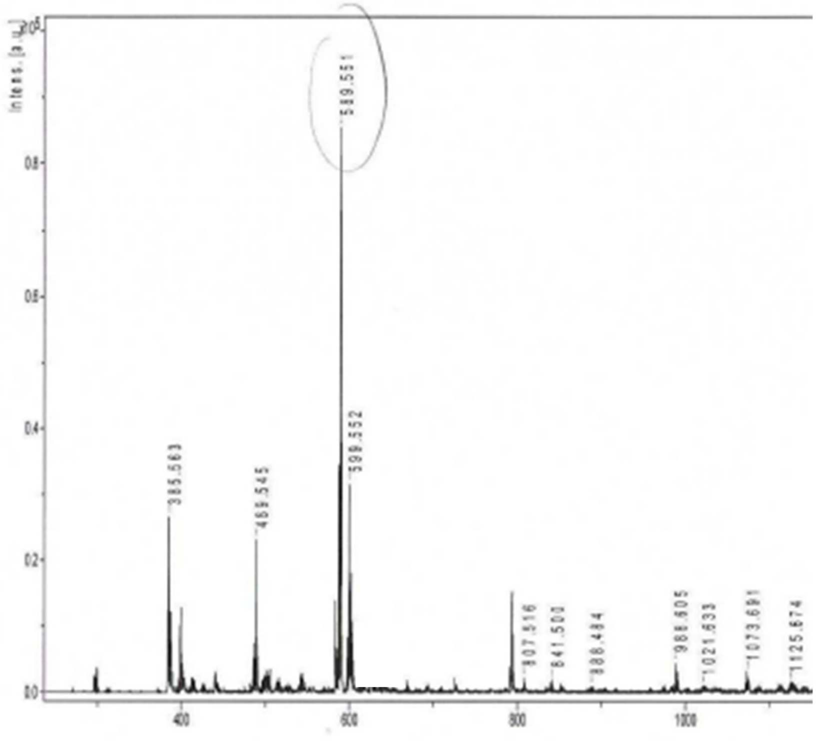

Figure S46. ESI-MS data for compound **12b**.

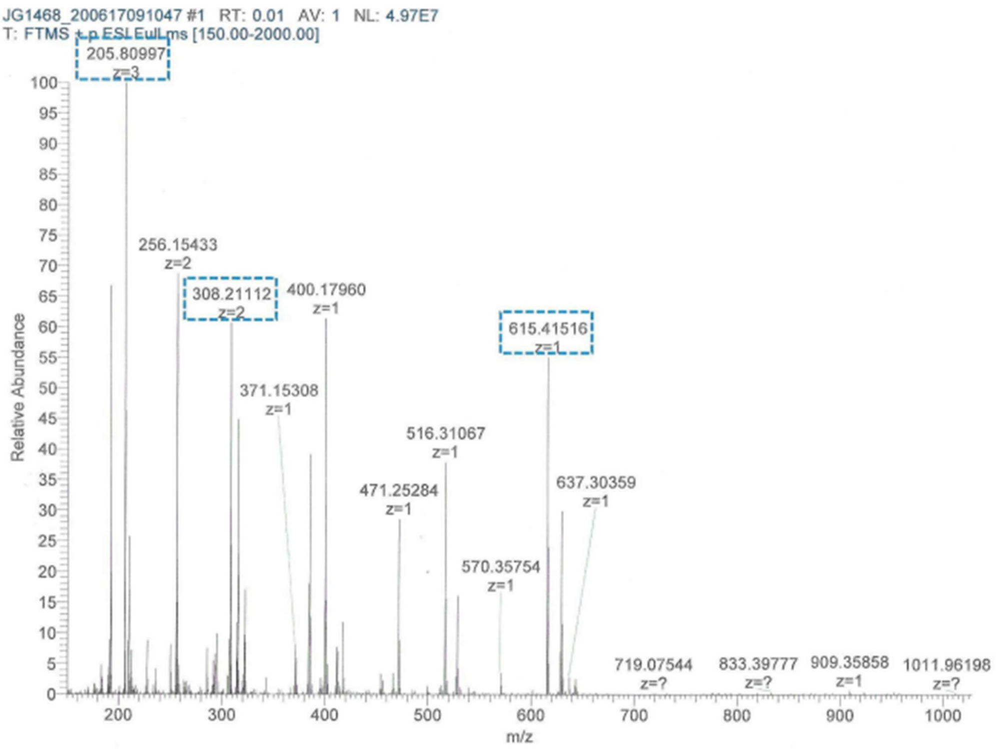

Figure S47. ESI-MS data for compound **12c**.

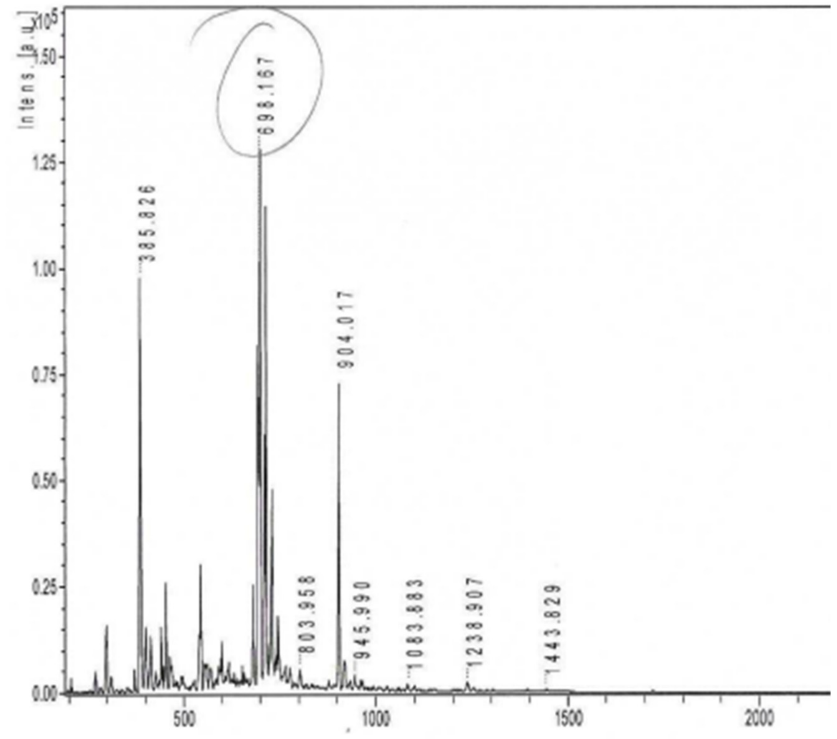

Figure S48. ESI-MS data for compound **12d**.

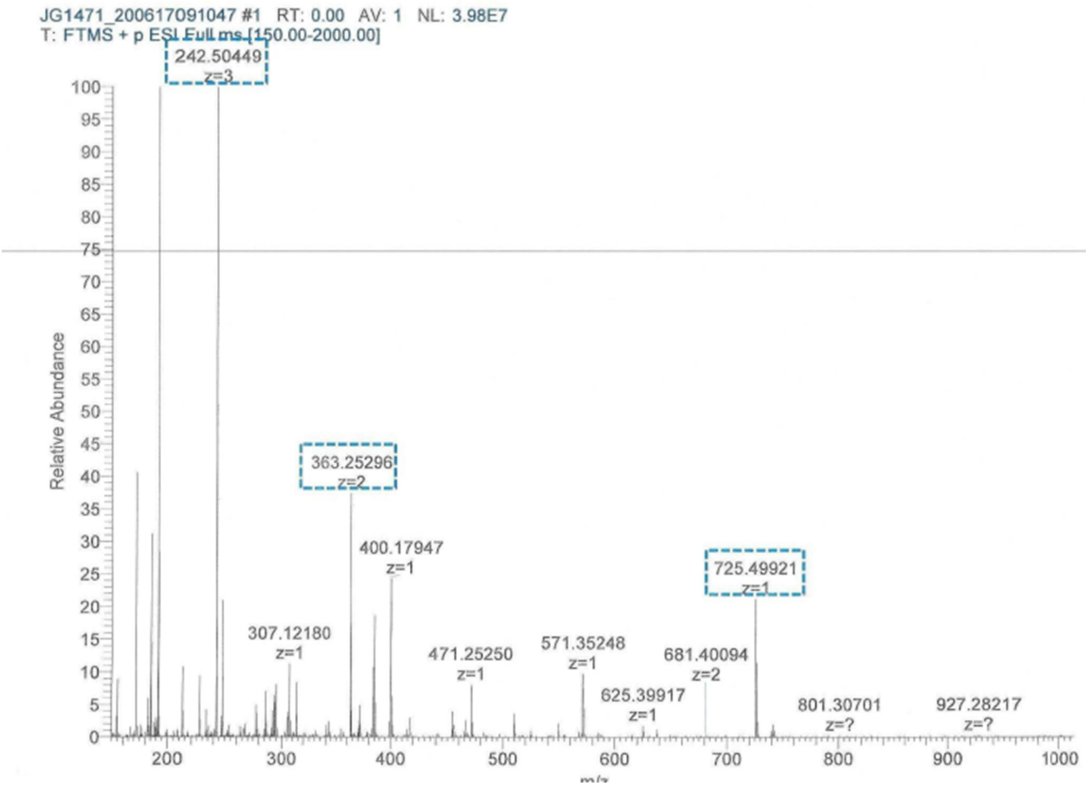

Figure S49. ESI-MS data for compound **12e**.

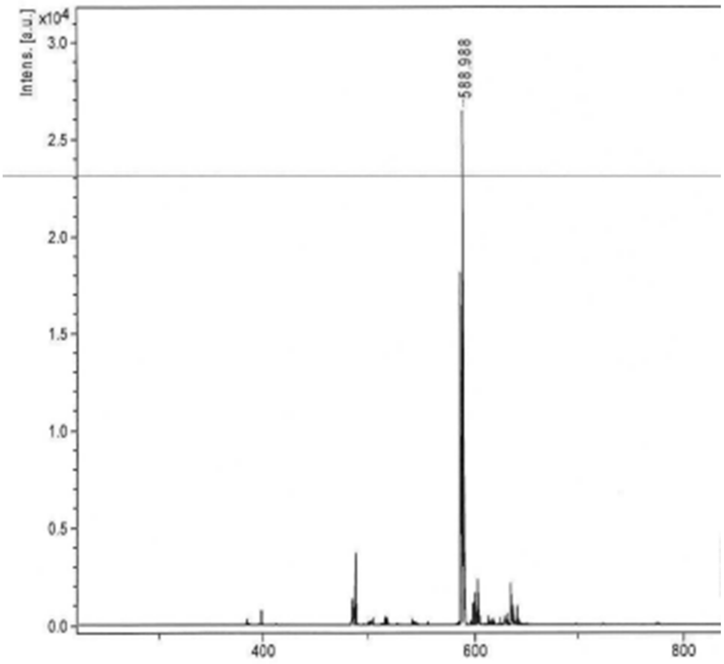

Figure S50. ESI-MS data for compound **12f**.

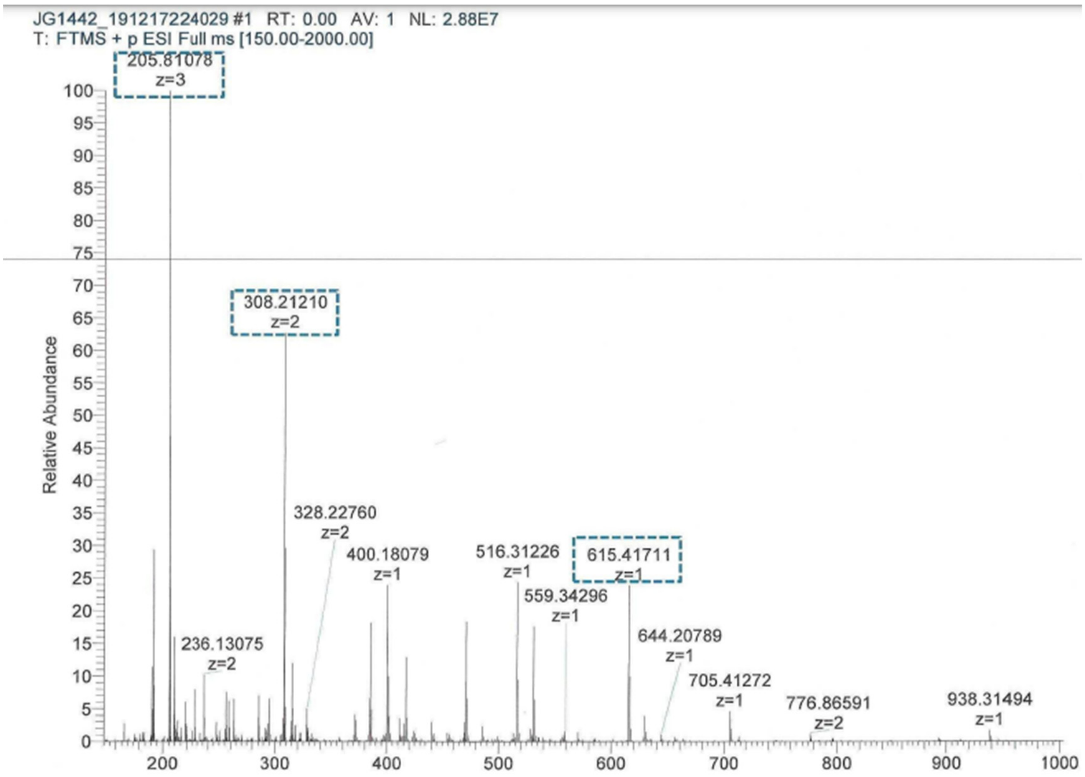

Figure S51. ESI-MS data for compound **12g**.

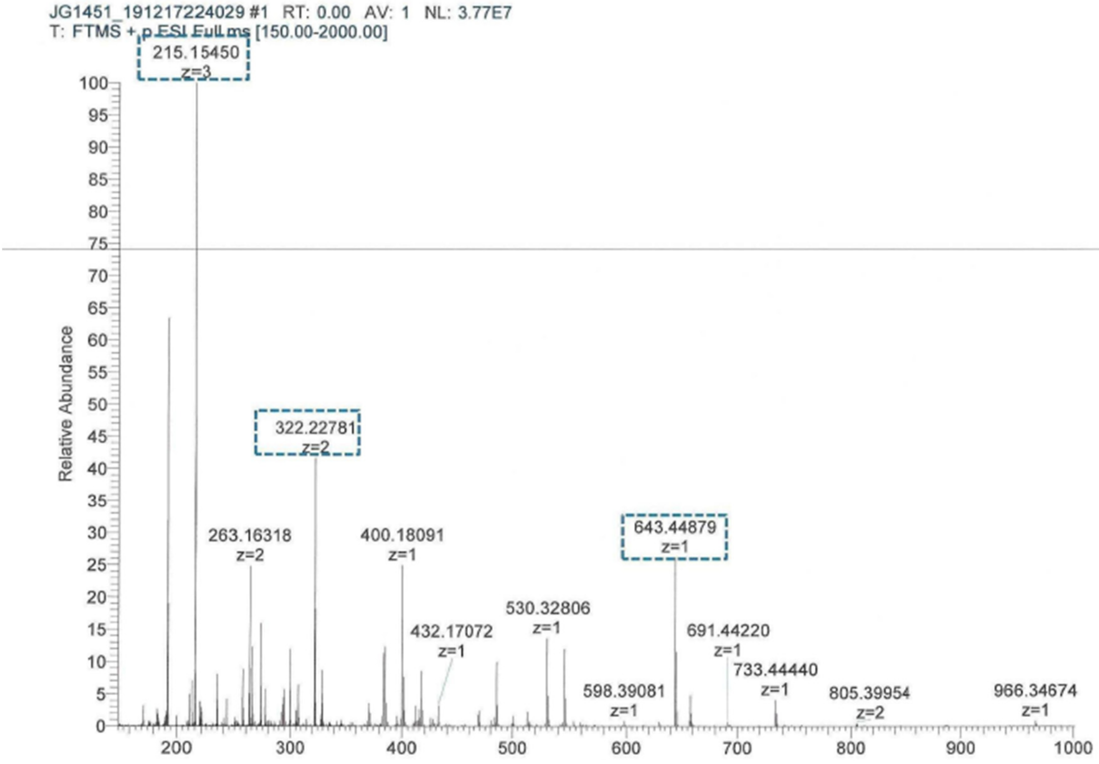

Figure S52. ESI-MS data for compound **12h**.

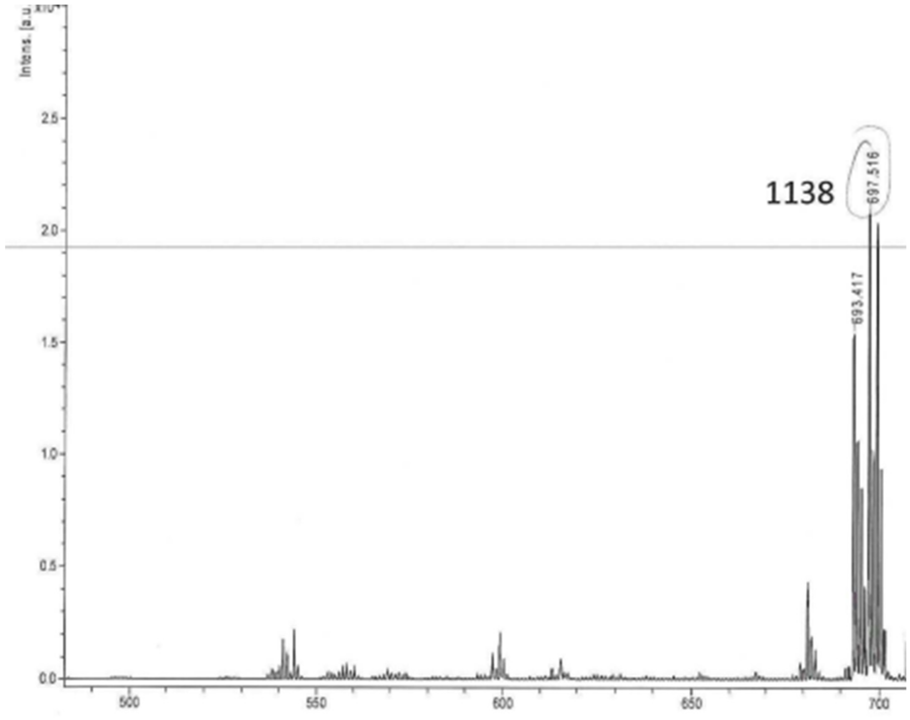

Figure S53. ESI-MS data for compound **12i**.

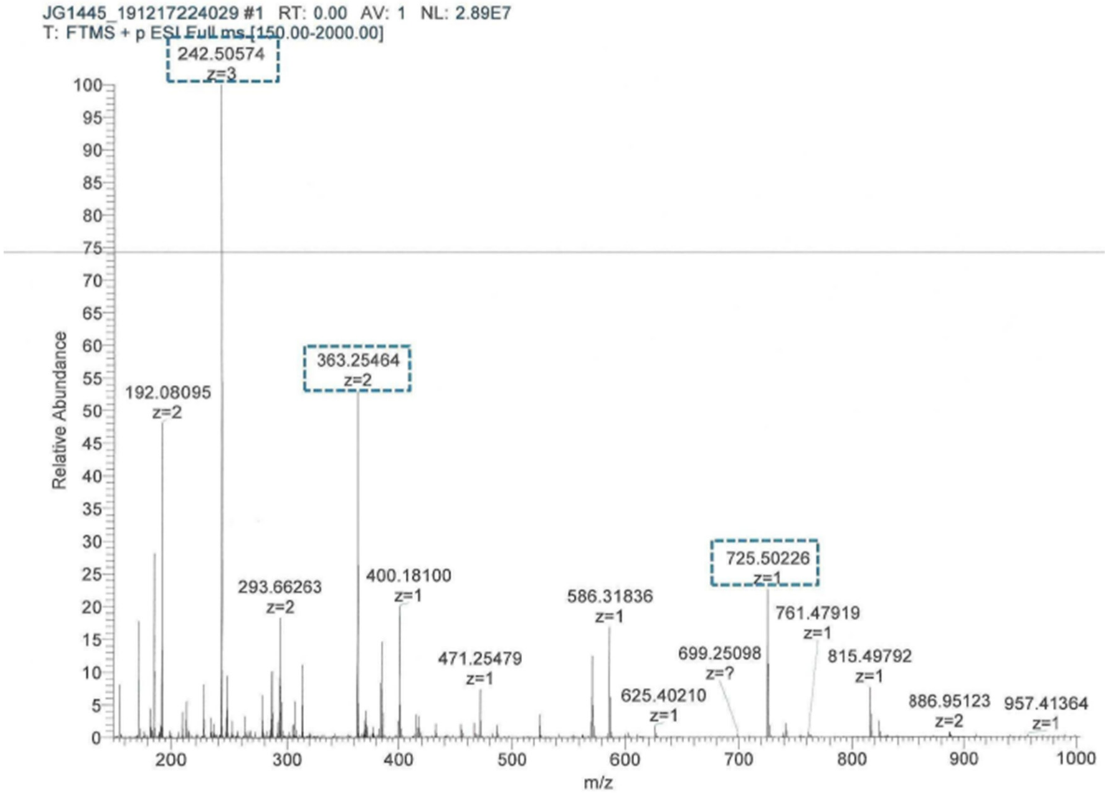

Figure S54. ESI-MS data for compound **12j**.

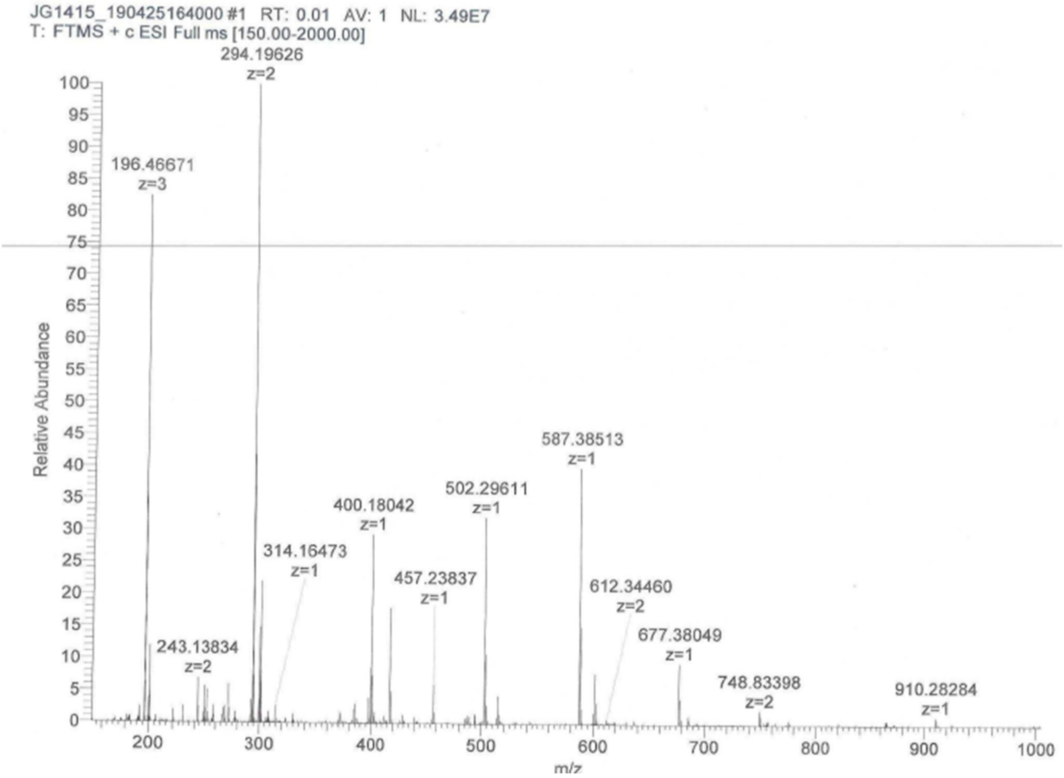

Figure S55. ESI-MS data for compound **12k**.

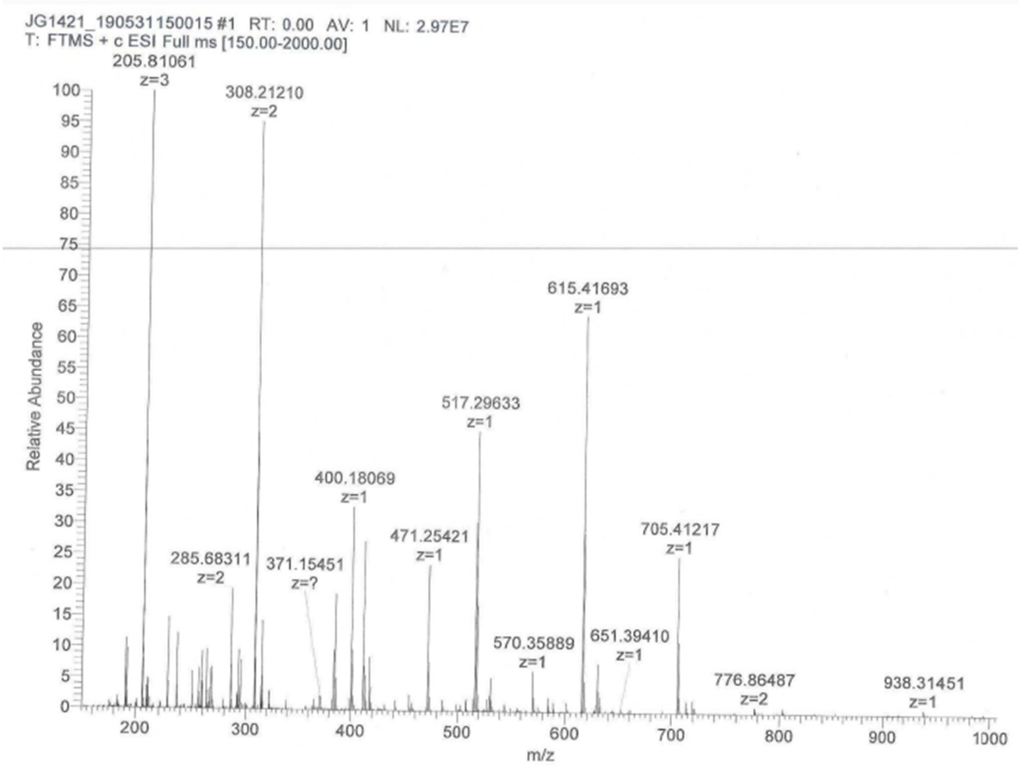

Figure S56. ESI-MS data for compound **12l**.

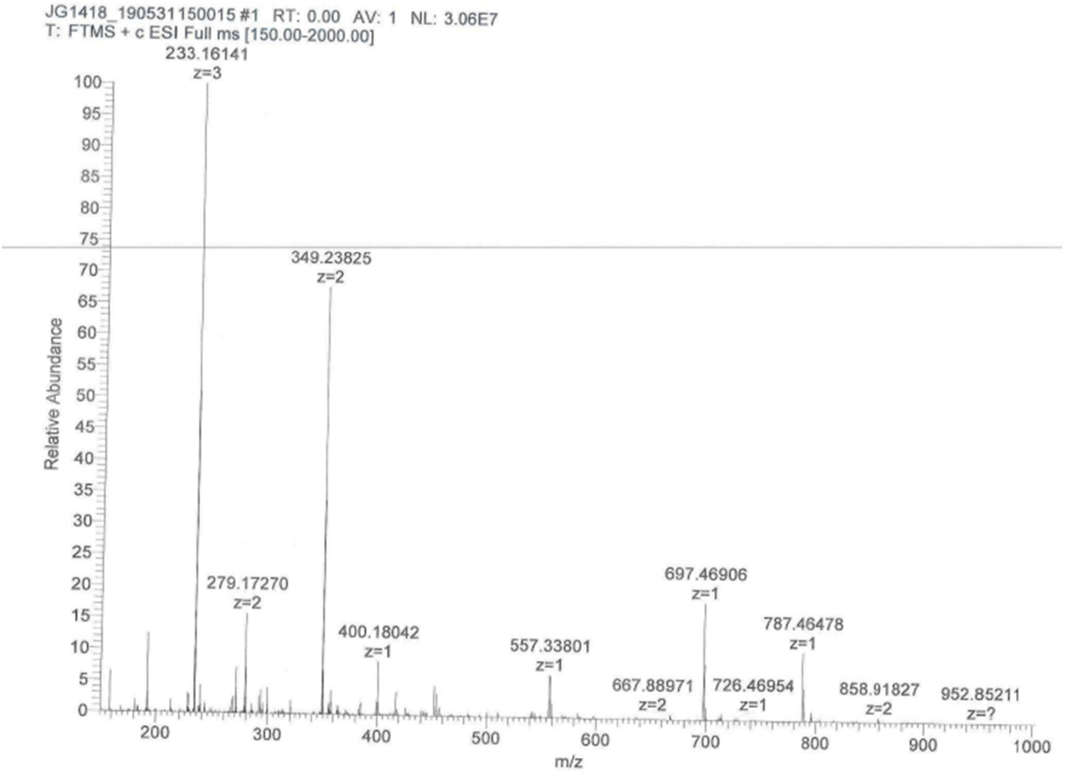

Figure S57. ESI-MS data for compound **12m**.

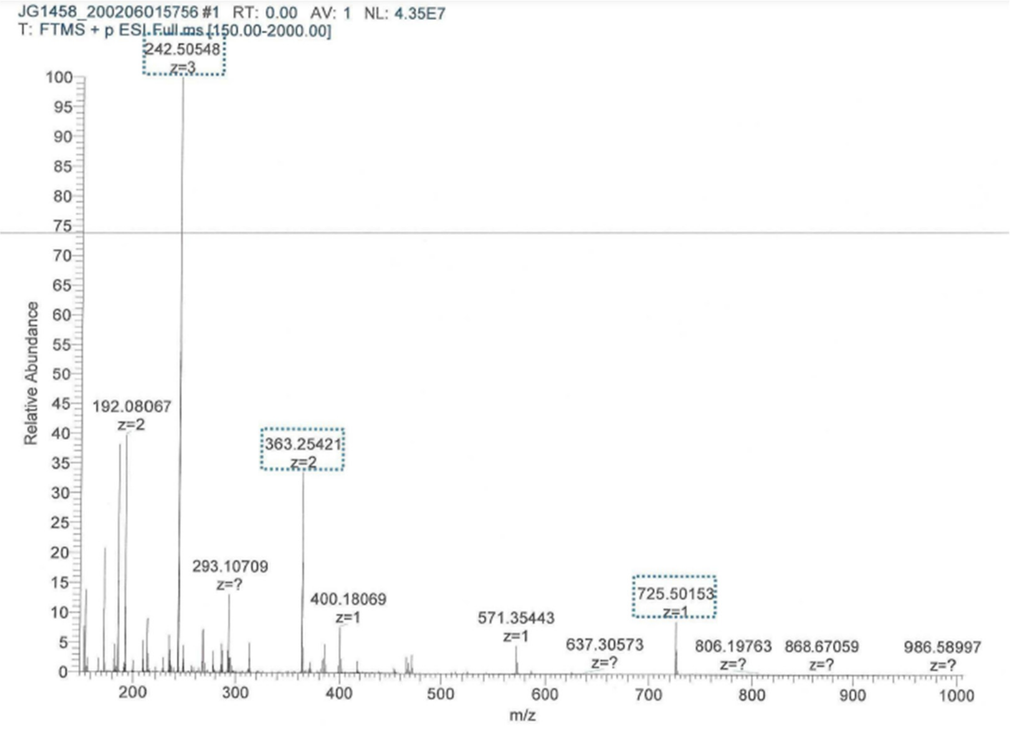

Figure S58. ESI-MS data for compound **12n**.

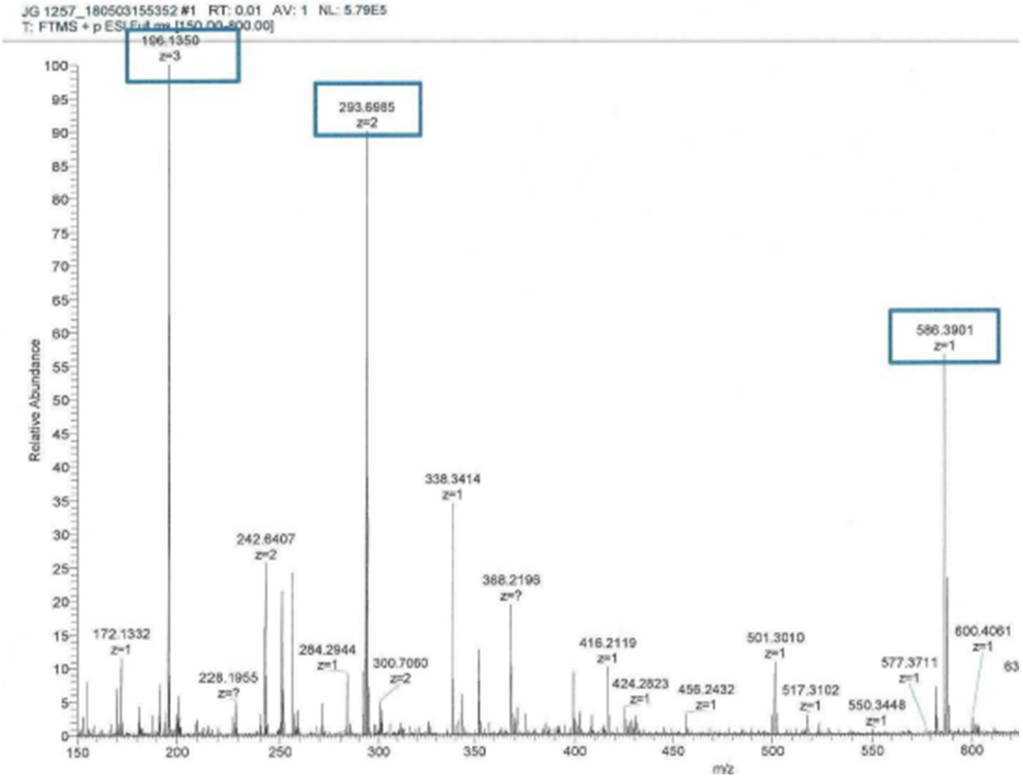

Figure S59. ESI-MS data for compound **13a**.

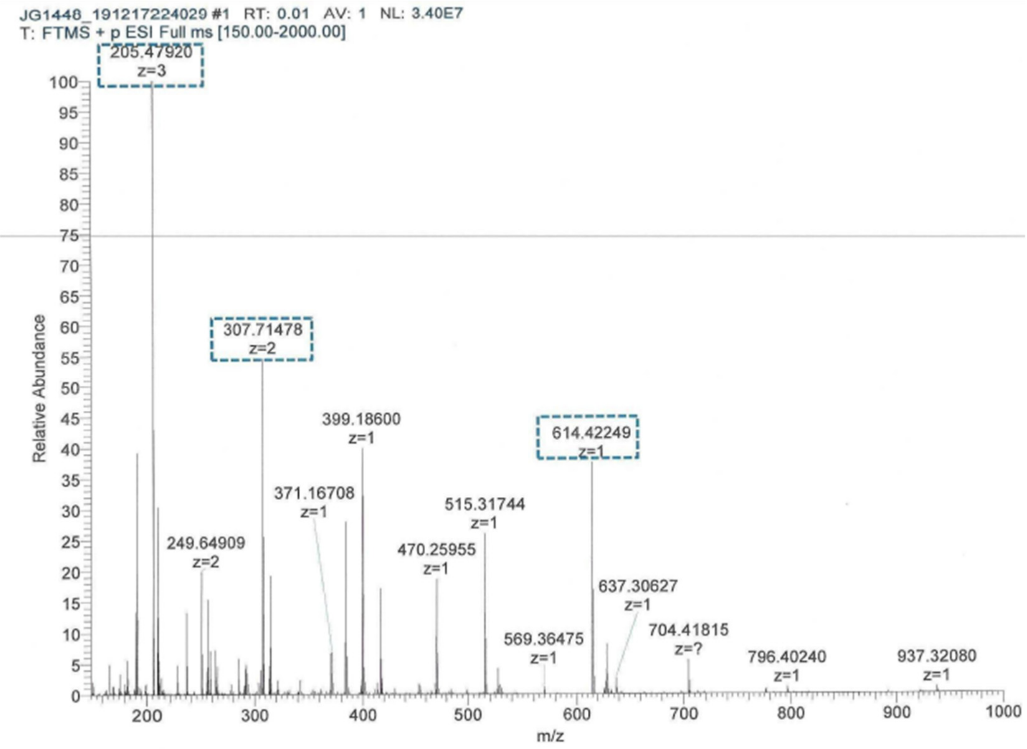

Figure S60. ESI-MS data for compound **13b**.

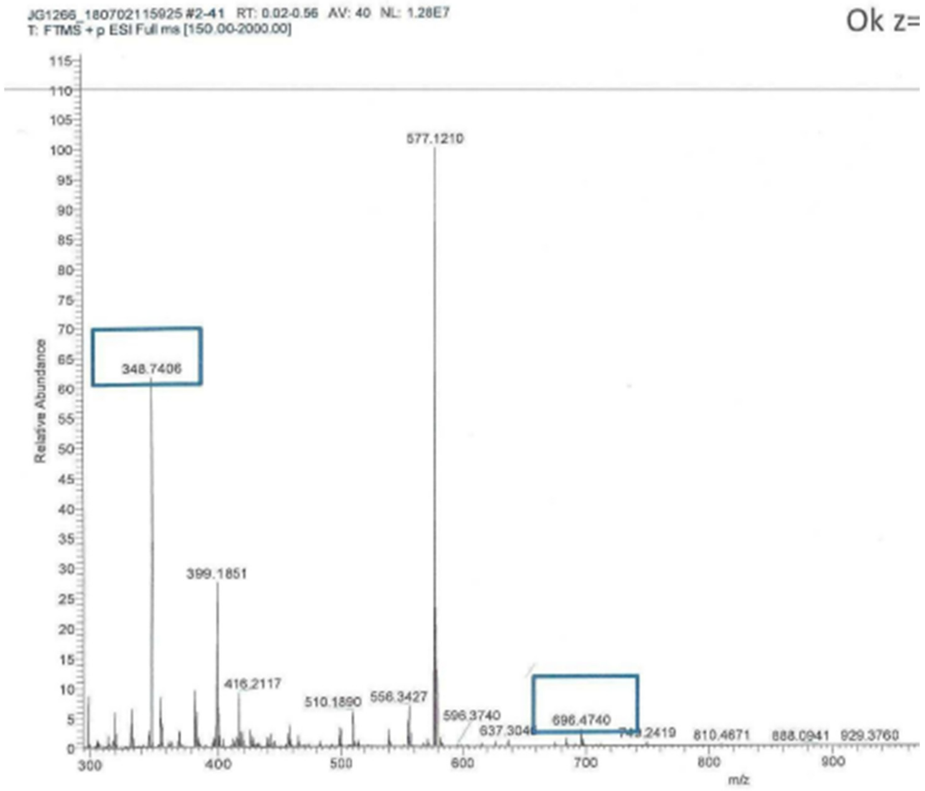

Figure S61. ESI-MS data for compound **13c**.

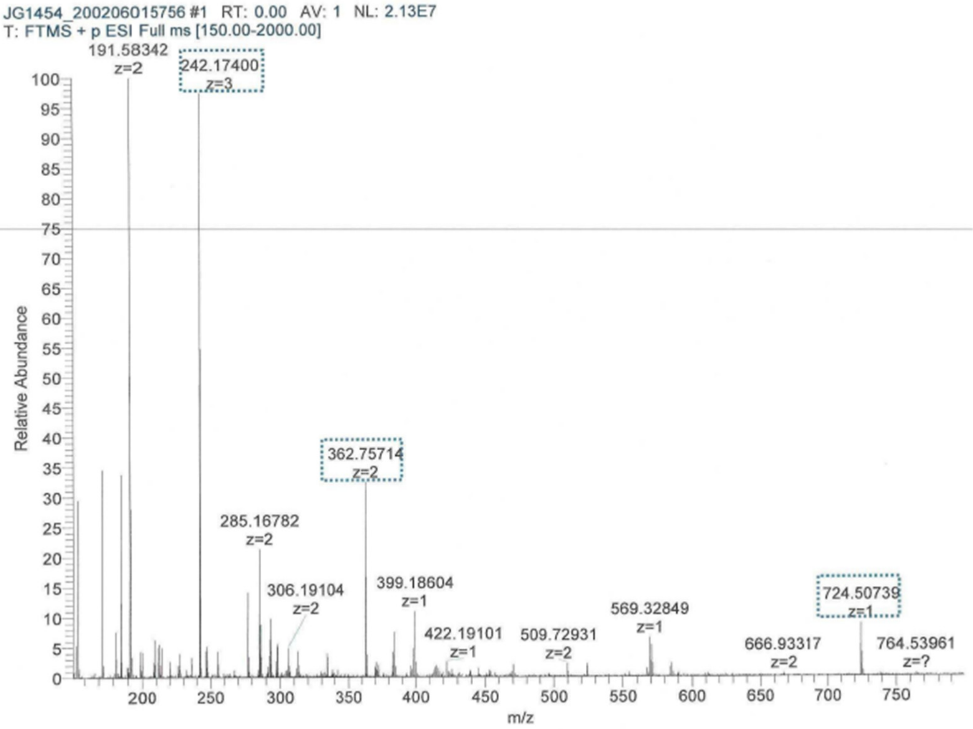

Figure S62. ESI-MS data for compound **13d**.

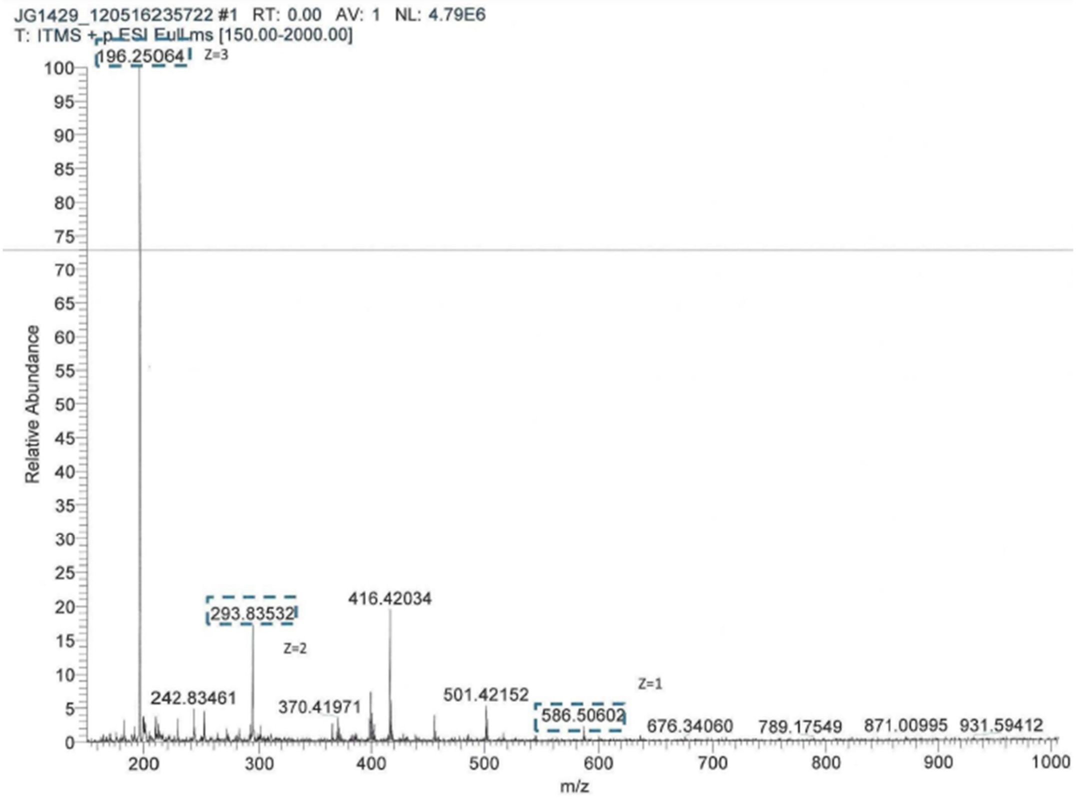

Figure S63. ESI-MS data for compound **13e**.
